# Monitoring Animal Populations With Cameras Using Open, Multistate, N‐Mixture Models

**DOI:** 10.1002/ece3.70583

**Published:** 2024-12-12

**Authors:** Alexej P. K. Sirén, Michael T. Hallworth, Jillian R. Kilborn, Chris A. Bernier, Nicholas L. Fortin, Katherina D. Geider, Riley K. Patry, Rachel M. Cliché, Leighlan S. Prout, Suzanne J. Gifford, Scott Wixsom, Toni Lyn Morelli, Tammy L. Wilson

**Affiliations:** ^1^ Department of Environmental Conservation University of Massachusetts Amherst Massachusetts USA; ^2^ Vermont Center for Ecostudies Norwich Vermont USA; ^3^ Vermont Department of Fish and Wildlife Rutland Vermont USA; ^4^ New Hampshire Fish and Game Department Concord New Hampshire USA; ^5^ Dartmouth College Woodlands Milan New Hampshire USA; ^6^ United States Fish and Wildlife Service, Silvio O. Conte National Wildlife Refuge Nulhegan Basin Division Brunswick Vermont USA; ^7^ United States Forest Service White Mountain National Forest Campton New Hampshire USA; ^8^ United States Forest Service Green Mountain National Forest Mendon Vermont USA; ^9^ U.S. Geological Survey Northeast Climate Adaptation Science Center Amherst Massachusetts USA; ^10^ U.S. Geological Survey Massachusetts Cooperative Fish and Wildlife Research Unit Amherst Massachusetts USA

**Keywords:** camera traps, hierarchical models, moose, multistate N‐mixture model, remote sensing, wildlife monitoring

## Abstract

Remote cameras have become a mainstream tool for studying wildlife populations. For species whose developmental stages or states are identifiable in photographs, there are opportunities for tracking population changes and estimating demographic rates. Recent developments in hierarchical models allow for the estimation of ecological states and rates over time for unmarked animals whose states are known. However, this powerful class of models has been underutilized because they are computationally intensive, and model outputs can be difficult to interpret. Here, we use simulation to show how camera data can be analyzed with multistate, Dail‐Madsen (hereafter multistate DM) models to estimate abundance, survival, and recruitment. We evaluated four commonly encountered scenarios arising from camera trap data (low and high abundance and 25% and 50% missing data) each with 18 different sample size combinations (camera sites = 40, 250; surveys = 4, 8, and 12; and years = 2, 5, 10) and evaluated the bias and precision of abundance, survival, and recruitment estimates. We also analyzed our empirical camera data on moose ( *Alces alces* ) with multistate DM models and compared inference with telemetry studies from the same time and region to assess the accuracy of camera studies to track moose populations. Most scenarios recovered the known parameters from our simulated data with higher accuracy and increased precision for scenarios with more sites, surveys, and/or years. Large amounts of missing data and fewer camera sites, especially at higher abundances, reduced accuracy, and precision of survival and recruitment. Our empirical analysis provided biologically realistic estimates of moose survival and recruitment and recovered the pattern of moose abundance across the region. Multistate DM models can be used for estimating demographic parameters from camera data when developmental states are clearly identifiable. We discuss several avenues for future research and caveats for using multistate DM models for large‐scale population monitoring.

## Graphical abstract

Our paper uses multistate Dail‐Madsen models to analyze camera data when multiple developmental states of unmarked animals are unambiguously identifiable. We demonstrate through simulation and empirical analyses that these models allow for the estimation of abundance, survival, recruitment, and changes in population size over time when animals are detected imperfectly.

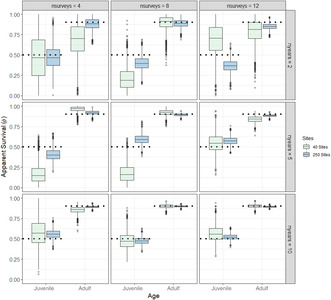

## 1 Introduction

The ability to track wildlife populations over space and time is an essential part of natural resource management and conservation (Murray and Sandercock 2020) and critical for addressing emerging and ongoing issues such as climate and habitat change (Mantyka‐Pringle, Martin, and Rhodes 2012). The development of remote camera technology since the 1990s has revolutionized our ability to monitor species over broad spatial and temporal scales (Burton et al. 2015; Steenweg et al. 2017). Remote cameras have been used for (1) estimating occurrence, abundance, and species richness (Burton et al. 2015), (2) evaluating activity and biotic interactions (Frey et al. 2017; Smith et al. 2020), and (3) collecting environmental data (Hofmeester et al. 2019; Sirén et al. 2018). More recently, camera data have been leveraged for estimating vital rates (e.g., recruitment) and tracking population dynamics (Chandler et al. 2018; Chitwood et al. 2017; Evans et al. 2024). However, the use of cameras for broad scale population monitoring is only a recent development (Steenweg et al. 2017), but it is one that could be explored for efficient single and multi‐species assessments.

Alongside growth in camera technology and applications for wildlife research, the advent of hierarchical models (HMs) for estimating ecological states and demographic rates when detection is imperfect emerged in the early 2000s (Royle and Dorazio 2008). In particular, the use of HMs for camera trap data is quite common for marked and unmarked populations (Gilbert et al. 2020). Most often, the detection history of a species captured on cameras is used for estimating patterns of species occurrence during one period (i.e., static occupancy models; Burton et al. 2015). However, there are HMs available for modeling changes in species occupancy (dynamic occupancy model; MacKenzie et al. 2003) or abundance (Dail‐Madsen model; Dail and Madsen 2011) over time that can also include multiple states or developmental stages (Mackenzie et al. 2009; Zipkin, Thorson, et al. 2014). The latter HMs are considered a powerful type of population model (Kéry and Royle 2020; Schaub and Kéry 2022) to estimate changes in population size and demographic rates and could be used for modeling data collected using cameras.

The development of the multistate, Dail‐Madsen model by Zipkin, Thorson, et al. (2014) – hereafter “multistate DM model”—provides a foundation for population monitoring that can be used with camera data of unmarked animals, provided that unique states are readily identifiable. The multistate DM model assumes a 4‐dimensional array where animals from each state (k) are counted at i sites, during j surveys, over t seasons or years. For this model, the initial abundance is estimated and transitions between states over time (either deterministic or stochastic) are modeled as a function of losses (apparent survival) and gains (apparent recruitment or immigration) (Zipkin, Thorson, et al. 2014). Covariates can be fit on the initial state as well as the transitions between states, and density‐dependent dynamics and environmental stochasticity can be integrated into the multistate DM modeling approach (Kéry and Royle 2020). However, besides Zipkin, Thorson, et al. (2014) and Kanno et al. (2015), few studies have examined the behavior of these models on simulated or empirical data. This is critical as different modeling approaches may result in divergent population trajectories that may or may not reflect real population dynamics. For example, N‐mixture models can overestimate population size due to unmodeled heterogeneity in detection or high variance in counts (Duarte, Adams, and Peterson 2018; Knape et al. 2018). Furthermore, if counts are sufficiently high then N‐mixture models might be computationally infeasible (Madsen and Royle 2023).

Many studies now regularly include > 150 camera sites that have been operating for at least 5 years (Rich et al. 2017; Sirén et al. 2021; Steenweg et al. 2019), providing unprecedented opportunities for modeling population dynamics across broad spatial extents (Steenweg et al. 2017). However, only a few studies have used camera data to estimate survival and recruitment (Chandler et al. 2018; Chitwood et al. 2017), and most research is focused on static occupancy and abundance (Burton et al. 2015). Although it is not always clear how to interpret abundance estimates from camera data of unmarked populations (Gilbert et al. 2020), conservative counts can be made for each site and survey (e.g., number of unique animals present by age class; Evans et al. 2024) and HMs (e.g., the multistate DM model) can be used to obtain site‐level estimates of abundance or relative abundance. Given that camera studies often employ a grid‐based design that approximates the spatial ecology of focal species (Hofmeester et al. 2021; Sirén et al. 2021; Steenweg et al. 2018), count data from these studies may be analyzed using DM (or multistate DM) models to estimate changes in abundance over time (Gilbert et al. 2020). However, there are numerous assumptions that can be evaluated to determine the accuracy of demographic parameters and population trajectories.

In this paper, we used both simulation and empirical data collected on moose ( *Alces alces* ) to illustrate how camera data of unmarked animals can be shaped into a multistate DM modeling framework to estimate abundance, apparent survival, recruitment, and changes in population size over time. We evaluated the bias and precision of parameter estimates from simulated data based on several scenarios that are often encountered using camera data (Burton et al. 2015; Kays et al. 2020). Specifically, we varied the sample sizes (number of cameras, years, and surveys/year) and initial abundances of two life stages (juvenile and adult) under low and high abundance scenarios, and used realistic demographic rates (apparent survival, recruitment) and a detection probability common for camera studies to determine how well the multistate DM model recovered known parameters. We also evaluated the impact of missing data—another normal attribute of camera studies that arises from several sources (e.g., dead batteries, staggered entry)—on the accuracy and precision of known parameters. We then estimated apparent survival and recruitment of moose from our empirical camera data with a multistate DM model and qualitatively compared results with telemetry studies from the same time period and region (Debow et al. 2021; Jones et al. 2017, 2019). We also introduce an R function for simulating data for multistate DM models (“multistate.DM”) as well as code for shaping and analyzing camera data of unmarked populations where multiple states are unambiguously observed. Lastly, we discuss sample size and design considerations for camera studies interested in modeling population dynamics and some potential caveats to consider for using count models.

## 2 Materials and Methods

### 2.1 Simulation Analysis

We amended the “simDM0” function from the *AHMbook* R package (Kéry, Royle, and Meredith 2022) to simulate multi‐season replicate count data for multiple states (in our case juvenile and adults), instead of a single state, assuming Poisson errors and imperfect detection (Sirén et al. 2024). We simulated low initial abundances of two developmental states (juvenile *λ* = 1; adult *λ* = 2) and set apparent survival (juvenile *φ* = 0.50; adult *φ* = 0.90) and recruitment (juvenile *γ* = 0.50; adult *γ* = 0.01) at different, yet biologically realistic (see studies in Gaillard et al. 2000; McCarthy, Citroen, and McCall 2008), levels for each developmental state. We varied the site sample sizes (i = 40, 250 cameras), survey lengths (j = 4, 8, 12 weeks), and primary survey periods or years (t = 2, 5, 10), yet kept site detection probability constant for both developmental states and at a level that is common for camera studies (*ρ* = 0.20; Steenweg et al. 2019). Here, we define sampling sites as independent camera locations that do not require a randomized design (e.g., placed alongside game trails; Gilbert et al. 2020). We also considered the influence of missing data on the ability of models (under the various scenarios) to recover known parameter estimates by randomly removing 25% and 50% of the data. Finally, we evaluated the ability of the models to recover population parameters when population sizes are higher. For this scenario, we used the same parameters as the previous simulations but set *λ* of juveniles and adults at 10 and 20, respectively. For each scenario, there were 18 combinations of site, surveys, and years (Table 1).

**TABLE 1:** Eighteen scenarios for evaluating the ability of multistate Dail‐Madsen (DM) models to recover initial abundances (low and high *λ*), apparent survival (*φ*), recruitment (*γ*), and detection probability (*ρ*) from simulated counts of juveniles and adults captured on camera traps with vary levels of effort (sites, years, and surveys) and missing data (25% and 50%). The unrecovered parameters (i.e., those that did not include the true simulated values within 95% credible intervals) are from simulations with low and high initial abundances (*λ*) and no missing data.

Sites (*n*)	Years (*j*)	Surveys (*k*)	Dimension (*n* × *j* × *k*)	Unrecovered (low *λ*)	Unrecovered (high *λ*)
40	2	4	320	Adult *γ*	Adult *γ*
250	2	4	2000	Juvenile *λ*	Juvenile *λ*, *ρ*; Adult *γ*
40	5	4	800	Juvenile *φ*, *γ*	Adult *γ*
250	5	4	5000	—	—
40	10	4	1600	Adult *λ*, *γ*, *ρ*	Adult *γ*
250	10	4	10,000	—	Adult *λ*, *ρ*
40	2	8	640	Adult *λ*, *γ*	Adult *γ*
250	2	8	4000	—	Adult *γ*
40	5	8	1600	Juvenile *φ*, Adult *γ*	Adult *γ*
250	5	8	10,000	Juvenile *λ*	—
40	10	8	3200	—	—
250	10	8	20,000	—	—
40	2	12	960	Adult *γ*	Adult *γ*
250	2	12	6000	—	Adult *γ*
40	5	12	2400	—	Adult *γ*
250	5	12	15,000	—	—
40	10	12	4800	—	Adult *γ*
250	10	12	30,000	—	—

We performed a Bayesian analysis using a modification of the multistate DM model developed by Zipkin, Thorson, et al. (2014) to estimate and recover parameters from the simulated data. Specifically, we modeled the abundance (N) for each developmental state (k_juveniles_ = 1; k_adults_ = 2) during the first time step (e.g., season, year) of sampling (t = 1) as $N_{k}{,}_{i}{,}_{1}∼\text{Poisson}\;\left(\lambda_{k}\right)$, where *λ*_*k*_ is the mean abundance for each state across all sites (i). We then modeled changes in abundance in subsequent time steps (t > 1) as a function of apparent survival (*ω*) and recruitment (*γ*) for juveniles (*ω*_1_, *γ*_1_) and adults (*ω*_2_, *γ*_2_)

$$S_{k=1}{,}_{i}{,}_{t}∼\text{Binomial}\;\left(N_{1}{,}_{i}{,}_{t-1},\omega_{1}\right)$$

$$S_{k=2}{,}_{i}{,}_{t}∼\text{Binomial}\;\left(N_{2}{,}_{i}{,}_{t-1},\omega_{2}\right)$$

$$G_{k=1}{,}_{i}{,}_{t}∼\text{Poisson}\;\left(\gamma_{1}N_{2}{,}_{i}{,}_{t-1}\right)$$

$$G_{k=2}{,}_{i}{,}_{t}∼\text{Poisson}\;\left(\gamma_{2}\right)$$

The population abundance in subsequent time steps (t > 1) for each developmental state (k) can then be calculated using the following equations:

$$N_{k=1}{,}_{i}{,}_{t}=G_{k=1}{,}_{i}{,}_{t}$$

$$N_{k=2}{,}_{i}{,}_{t}=S_{k=1}{,}_{i}{,}_{t}+S_{k=2}{,}_{i}{,}_{t}+G_{k=2}{,}_{i}{,}_{t}$$

Finally, we accommodate imperfect detection of individuals counted (n) from each developmental state (k) using the following likelihoods of the observation process, where *ρ*_1_ and *ρ*_2_ are the detection probabilities of juveniles and adults, respectively, at each site (i) during each sampling occasion (j) and time period (t):

$$n_{k=1}{,}_{i}{,}_{j}{,}_{t}∼\text{Binomial}\;\left(N_{k=1}{,}_{i}{,}_{t},\rho_{1}\right)$$

$$n_{k=2}{,}_{i}{,}_{j}{,}_{t}∼\text{Binomial}\;\left(N_{k=2}{,}_{i}{,}_{t},\rho_{2}\right)$$

We specified uninformed priors on model parameters and used Markov chain Monte Carlo (MCMC) methods with the *jagsUI* R package (Kellner 2021), which is a wrapper for JAGS software (Plummer 2017), to draw samples from the posterior distribution. We ran 3 MCMC chains of 110,000 iterations with an adaptive phase of 1000 iterations that was followed by an initial burn‐in phase of 10,000 iterations. We drew inference from the posterior distribution by saving every 100th iteration, resulting in 1000 samples per MCMC chain. The JAGS code for the simulation is provided online (Sirén et al. 2024). We inspected each model and parameter for convergence using trace plots and $\hat{R}$ statistics. The $\hat{R}$ is a test statistic that evaluates variance between and within MCMC chains, assuming convergence when $\hat{R}$ values are < 1.1 (Gelman and Rubin 1992). If models failed to converge, we increased the number of iterations and/or burn‐in phase, changed the thinning rate so that it resulted in 1000 samples per MCMC chain, and reran models.

We assessed the ability of models to recover known parameters (*θ*) of scenarios by calculating the mean, standard deviation, and the 2.75% and 97.5% quantiles of the relative absolute deviation (RAD), expressed as:

$$\mathrm{RAD}=\frac{\left|N_{i,k}-\theta_{k}\right|}{\theta_{k}}\times 100\%$$

where RAD is the percent difference between the known parameters and the posterior samples $N_{i}$ of each developmental state (k). We considered RADs with lower means and standard deviations (and shorter range between 2.75% and 97.5% quantiles) to be more accurate and precise, respectively (Berg, Erb, and Westphal 2024).

### 2.2 Empirical Analysis

Moose are a charismatic and culturally important ungulate found in the Holarctic temperate and boreal forests that have a strong influence on ecosystem dynamics (Franzmann and Schwartz 2007; Timmermann and Rodgers 2017; Wattles and DeStefano 2011). Many southern populations have declined across the Holarctic range over the past several decades likely due to climate change and negative biotic interactions (Debow et al. 2021; Jones et al. 2017; Murray et al. 2006; Ruprecht et al. 2016; Spong et al. 2020). Consequently, there has been increased focus on identifying the mechanisms behind the declines and developing efficient and robust approaches for monitoring populations (Hinton et al. 2022; McMahon et al. 2021; Moll et al. 2022).

However, it is not trivial to collect demographic information (abundance, survival, recruitment) at large spatial scales for effective population monitoring (Moll et al. 2022), especially for low‐density populations (Hinton et al. 2022). For example, estimating calf (0‐ to 12‐month‐old individuals) and adult survival rates—important parameters that influence moose population growth rates (Gaillard 2007) – has typically been done at local scales using intensive and costly telemetry studies (Ellingwood et al. 2020; Murray et al. 2006, 2012). Further, obtaining information on the abundance of different age classes and recruitment rates are critical ingredients for population modeling and management (e.g., setting harvest limits), yet are challenging and expensive to obtain (e.g., aerial surveys, Moll et al. 2022). A potential solution is to use annual counts of calf and adult moose from remote cameras (Moll et al. 2022), as both developmental states are identifiable in pictures (Found and Patterson 2020). These data may be analyzed with multistate DM models to obtain estimates of abundance and infer apparent survival and recruitment to track changes in moose populations over time.

We used count data of calf and adult moose from a large, long‐term camera trap network in the northeastern United States to evaluate the ability of multistate DM models to produce site‐level population estimates, apparent survival and recruitment, and calf: adult cow (female) ratios (Sirén et al. 2024). We compared outputs with two telemetry studies that were conducted simultaneously in the same region (Debow et al. 2021, 2022; Jones et al. 2017, 2019). Our network included 225 camera sites (established in non‐overlapping 2 × 2 km grids) that were operating from 2014 to 2019 and distributed over a large latitudinal (42.9°–45.3°N) and elevational (3–1451 m) gradient. Each site included a remote camera pointed at a snow stake positioned ~5 m away. We programmed cameras to take 1–3 pictures every 1–10 s when triggered, depending on the camera brand and model. Skunk essence and feathers were used as olfactory and visual attractants, respectively, and attached on the top of the stakes. We checked cameras three (range = 1–9) times each year to download data and to ensure attractants and cameras were working properly. For more information on the camera method, please see Sirén et al. (2018).

We fit a three‐state DM model with only two observable developmental states (calves and adult females [cows] but not yearlings) to model changes in moose population size over time. This model is useful for organisms whose developmental states advance in a deterministic manner and do not reproduce until the third year of life (see detailed description and model formulation in Zipkin, Thorson, et al. 2014). Although some moose yearlings can reproduce, it is rare in much of the southern part of its range in North America, and the majority of reproduction occurs from animals ≥ 3 years old (Debow et al. 2022; Jones et al. 2017; Ruprecht et al. 2016). Further, it can be difficult to differentiate between yearlings and adults during capture (Jones et al. 2017), and this would logically extend to differentiating between these developmental state from pictures. As such, we used the approach that Zipkin, Thorson, et al. (2014) applied for salamanders where there were two observable developmental states (juveniles and adults) and a three‐state formulation was adopted to account for delayed reproduction. This model includes the following parameters: initial abundance for 3 age classes (1st and 2nd years juveniles and adults), apparent survival for two developmental states (juveniles and adults), apparent recruitment of adults or reproductive rate, and the immigration rate of juveniles and adults (Zipkin, Thorson, et al. 2014). Lastly, we could not differentiate between male and female calves, which is common at this life stage (Jones et al. 2017), and so these sex classifications were pooled.

We fit a multistate DM model that included 6 years (2014–2019), where populations were open, with 4 month‐long survey occasions per year (May–August), where populations were assumed to be closed. To avoid double counting, we only considered the maximum number of calves and adult cows detected within pictures at a site during a monthly survey occasion. We specified uninformative and uniform priors on initial abundance and recruitment but constrained calf survival to 0.2 and 0.8 and detection probability for calves and adults to 0.1 and 0.9; the informed priors on these parameters were chosen to improve model convergence and reflect known survival rates for calf moose (Ellingwood et al. 2020) and expected detection probability of moose on cameras (Steenweg et al. 2019). We used the same MCMC settings as the simulation analysis, except that our adaptive phase was 10,000 iterations. The JAGS code for the empirical analysis is provided online (Sirén et al. 2024). As with the simulation analysis, we inspected convergence using trace plots and $\hat{R}$ statistics, assuming convergence when $\hat{R}$ values were < 1.1 (Gelman and Rubin 1992).

## 3 Results

### 3.1 Simulation Analysis

**FIGURE 1:**
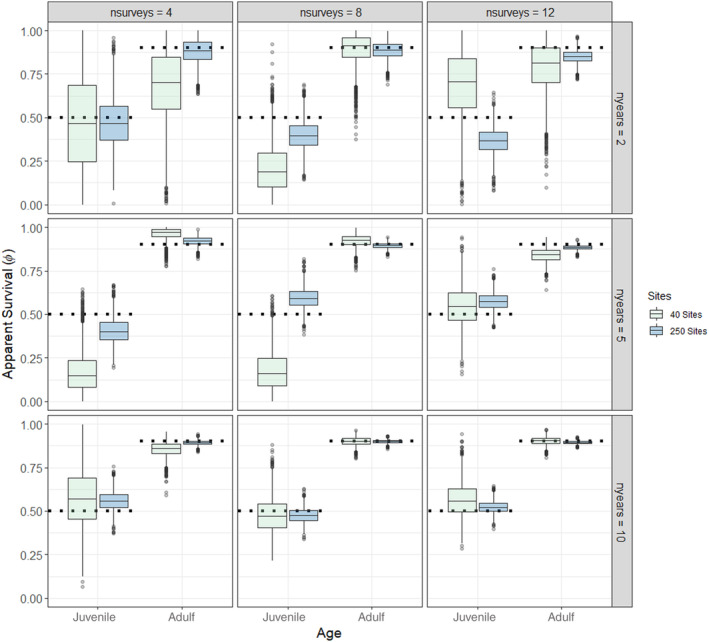
Boxplots of Monte Carlo Markov chain (MCMC) draws (*n* = 3000) of simulated apparent survival for juveniles and adults compared to known survival (horizontal dashed lines) under 18 different sampling scenarios assuming a low initial abundance with no missing data and equal detection probability for both age classes (*ρ* = 0.2). For each plot, the shaded box represents the upper and lower quartiles, and the horizontal solid line represents the median of the MCMC draws. The vertical lines represent 99% of the draws and the open circles represent outliers.

**FIGURE 2:**
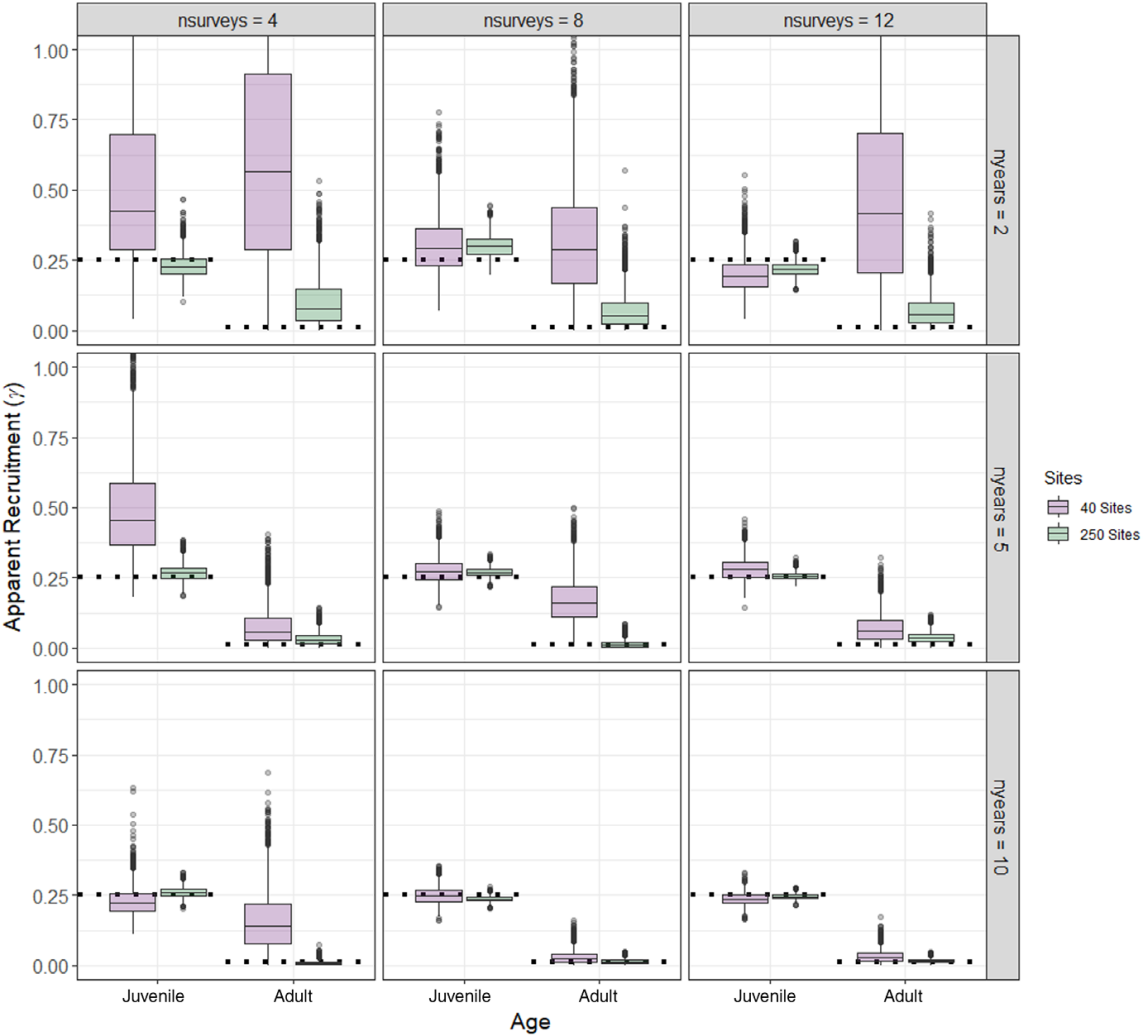
Boxplots of Monte Carlo Markov chain (MCMC) draws (*n* = 3000) of simulated apparent recruitment/immigration for juveniles and adults compared to known recruitment (horizontal dashed lines) under 18 different sampling scenarios assuming a low initial abundance with no missing data and equal detection probability for both age classes (*ρ* = 0.2). For each plot, the shaded box represents the upper and lower quartiles, and the horizontal solid line represents the median of the MCMC draws. The vertical lines represent 99% of the draws and the open circles represent outliers. Note that the *y*‐axis is limited between 0 and 1 to improve visual comparison across scenarios but the outliers of some boxplots extend considerably higher for some scenarios (e.g., the highest value for apparent recruitment was 45 under a scenario with 40 sites, 2 years, and four surveys).

**FIGURE 3:**
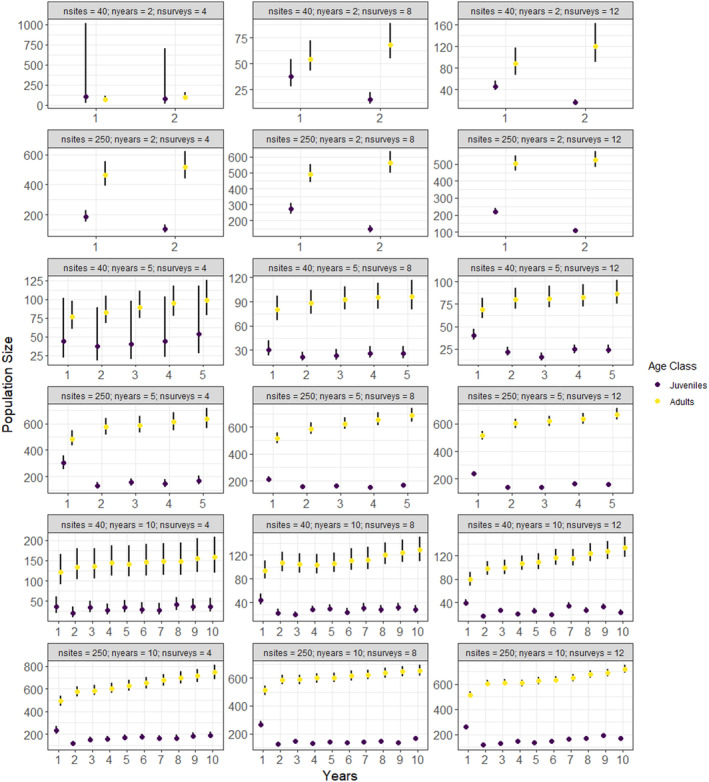
Simulated estimates of population size and 95% credible intervals for each age class under 18 different sampling scenarios assuming a low initial abundance with no missing data and equal detection probability for both age classes (*ρ* = 0.2). The circles indicated parameter estimates, and the lines indicate the 95% credible intervals.

The models recovered the true (simulated) values for initial abundance, apparent survival, recruitment, and detection probability for 92% (528 of 576) of the parameters under both low and high abundance scenarios and those with missing data (Table 1, Tables S1–S4). Convergence was achieved for all but 2 of the scenarios (Scenarios 4 and 16 where 50% of the data were missing, Table S3). As expected, the accuracy (as evaluated using mean RAD) and precision (as evaluated by standard deviation and 2.75% and 97.5% quantiles of RAD) increased with higher sample sizes (Figures 1, 2, 3, Table S5, Figures S1–S17); however, there were some biases in apparent survival and recruitment related to sample size and missing data that influenced annual population trajectories. We did not find any substantial differences in the ability of the models to recover parameters for low and high initial abundances, except that adult recruitment was less likely to be recovered at high abundances, especially when there were fewer sites (Table 1).

Overall, scenarios recovered the true values of starting abundance for each age class, except in scenarios with fewer sites or surveys where estimates were biased low (Table 1, Tables S1–S4). There was more variability in initial abundance for the scenarios with 40 sites, especially with fewer surveys per season, and when there were missing data (Figures S1–S4). RAD was high and variable (mean > 129%; standard deviation > 185%) for initial abundance at the lowest sample size scenarios, indicating inaccuracy and uncertainty (Table S5, Figure S18). Similarly, the true values of detection probability were recovered for most scenarios (Table 1, Tables S1–S4) and, similar to the pattern with other parameters, accuracy and precision increased with sample size (Table S5, Figures S5–S8, S19).

However, estimates of apparent survival and recruitment were more sensitive to changes in sample size, missing data, and abundance levels. Apparent survival of both age classes, but especially juveniles, was imprecise for scenarios that had low and high initial abundance, fewer sites, surveys, and years, and when data were missing (Figure 1, Figures S9–S11, S20). There were several low sample size scenarios where the model underpredicted survival, primarily juvenile survival, and was unable to recover the true estimates (Table 1, Tables S1–S4). Indeed, RAD was high and variable (mean > 60%; standard deviation > 21%) for apparent survival of juveniles at the lowest sample size scenarios, indicating inaccuracy and uncertainty (Table S5, Figure S20). Apparent recruitment was the most difficult parameter for the models to recover and was sensitive to the number of sites, surveys, and years (Figure 2, Figures S12–S14). For scenarios with the lowest sample sizes (e.g., 40 sites and 2 years), the median estimates of recruitment of juveniles and immigrating juvenile and adults were overestimated at low and high abundance levels (Figure 2, Figure S14), and this was most prevalent when 50% of the data were missing (Tables S3, Figure S13). RAD was extremely high and variable (mean > 1000%; standard deviation > 783%) for apparent recruitment of adults except for the highest sample size scenarios, indicating inaccuracy and uncertainty (Table S5, Figure S21). Juvenile recruitment was biased high for all scenarios when 50% of the data were missing (Figure S13) and the true values of adult and juvenile recruitment were overestimated and not recovered for many of the scenarios with 40 sites and fewer surveys and years (Table 1, Tables S1–S4). Finally, adult recruitment/immigration was overestimated for most high abundance scenarios when there were only 40 sites (Figure S4) and RAD even remained high and variable (mean = 331.94%; standard deviation = 383.46%) for some of the largest sample size scenarios (Table S5, Figure S20).

Sample size and missing data influenced apparent survival and recruitment which had a direct impact on population trajectories. Annual population size was stable for most scenarios except for when 50% of the data were missing (Figure 3, Figures S15–S17). Here, the overestimation of recruitment resulted in increasing population size for all scenarios (Figure S16). Unsurprisingly, the precision of annual population size increased with sample size (primarily the number of sites) due to the propagation of errors that arise from annual losses (survival) and gains (recruitment) (Figure 3, Figures S15–S17).

### 3.2 Empirical Analysis

We recorded 568 and 192 monthly detections of adult female and juvenile moose, respectively. Of these, we documented > 1 adult female and > 1 juvenile moose at a site during a monthly occasion 2 and 7 times, respectively. Due to a combination of issues (e.g., battery failure, camera malfunction) and the staggered entry of cameras into the study, 45% of the data were missing.

**FIGURE 4:**
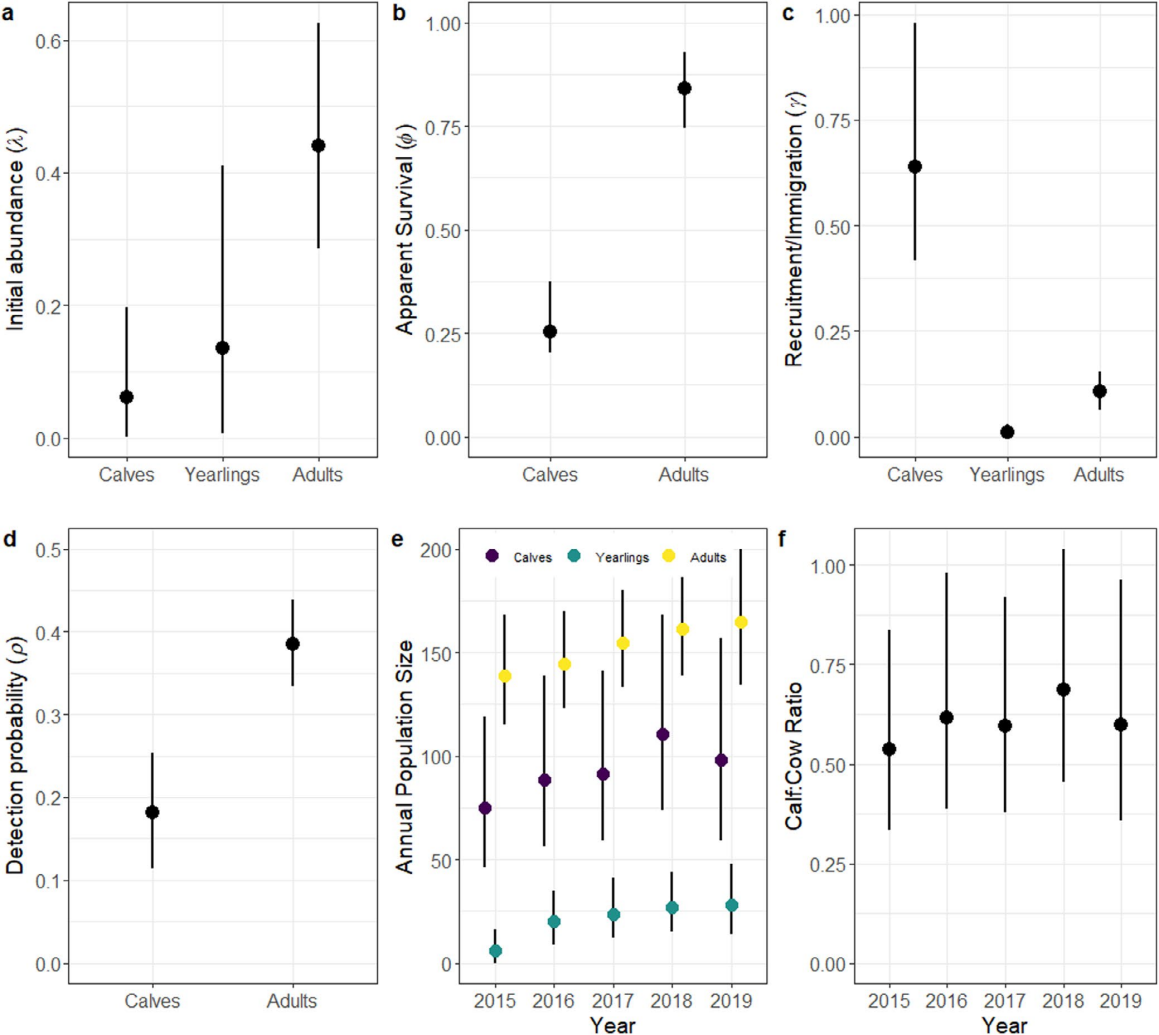
Posterior means and 95% credible intervals of (a) initial site abundance of moose ( *Alces alces* ) calves, yearlings, and adults, (b) apparent survival of moose calves and adults, (c) recruitment rate of juvenile moose and gains from immigration of yearling and adult moose, (d) detection probability of moose calves and adults, (e) population trajectories for moose calves, yearlings, and adults, and (f) calf: Adult cow ratios from 2015 to 2019 in Vermont and New Hampshire USA.

**FIGURE 5:**
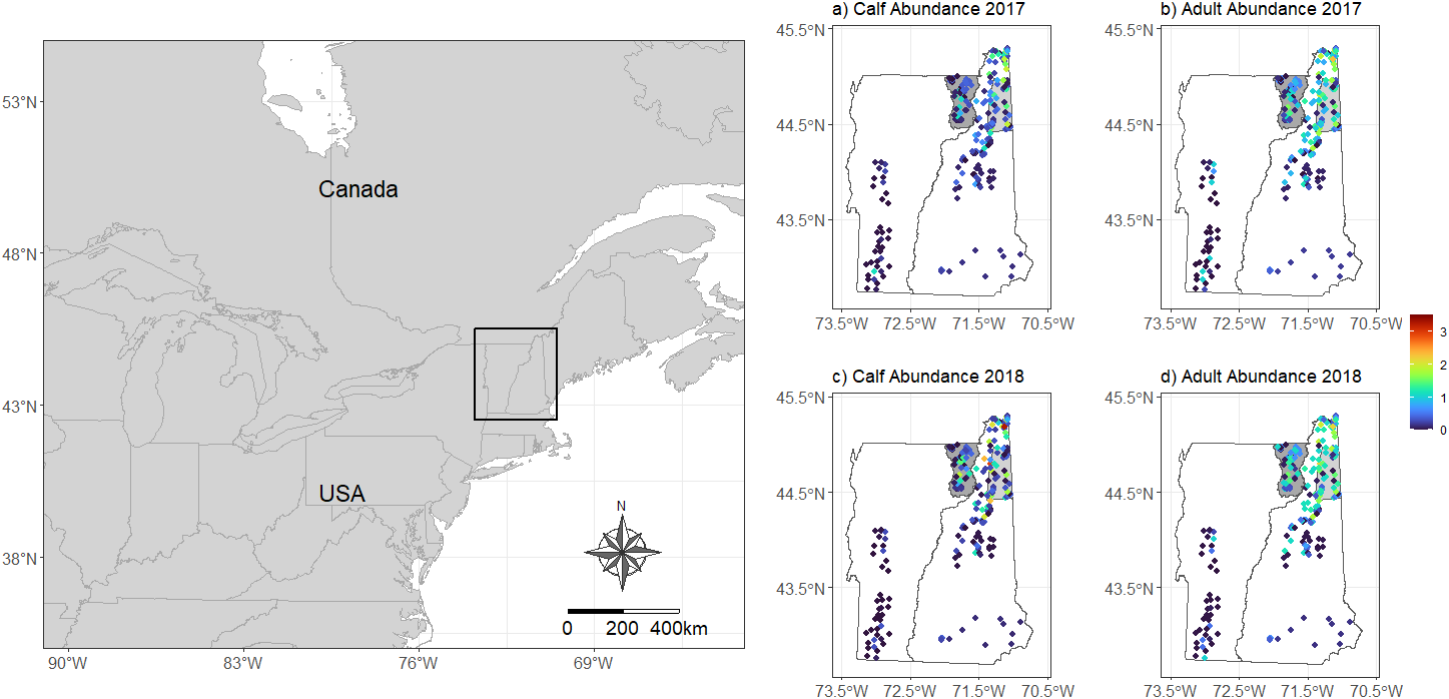
Posterior estimates of annual, site‐level abundance for moose ( *Alces alces* ) calves and adult cows from 2017 to 2018 in Vermont and New Hampshire USA. The dark and light gray regions of Vermont and New Hampshire, respectively, are study regions where moose were radio‐collared to estimate survival, reproduction, and habitat use from 2014 to 2019 (Debow et al. 2021, 2022; Ellingwood et al. 2020).

Most of the parameters for the multistate DM moose model converged (i.e., $\hat{R}$ values were < 1.1), except for a small proportion (3%; 436 of 13,903) of site by year parameters (N, S, G), and the MCMC chains mixed well with some evidence of truncation for juvenile survival (Figure S22). Initial site abundance was lower for calf and yearling moose compared to adults with higher uncertainty for the yearling moose (Table 2, Figure 4a). Apparent survival was higher for adults (*φ* = 0.85; 0.75–0.93 95% credible interval [CI]) than calves (*φ* = 0.25; 0.20–0.37 95% CI) (Table 2, Figure 4b). Calf recruitment was estimated at 0.64, yet variable (0.42–0.99 95% CI), whereas yearling and adult immigration were low and precisely estimated (Table 2, Figure 4c). Detection probability was lower for calves (*ρ* = 0.18; 0.11–0.25 95% CI) than adults (*ρ* = 0.38; 0.33–0.44 95% CI) with high precision for both age classes (Table 2, Figure 4d). The annual population size of each age class remained relatively stable during the 6 years of monitoring, with more variability for calves compared to yearling and adult moose (Figure 4e). Calf: adult cow ratios (calves per adult cow) remained stable over time averaging ~0.6 calves per adult cow (Figure 4f). Abundance followed a latitudinal gradient with the highest abundances for calves and adult cows at higher latitudes (Figure 5).

**TABLE 2:** Posterior means, standard deviations (SD), and lower and upper 95% credible intervals from a multistate Dail‐Madsen (DM) model used to estimate initial site abundance (*λ*), apparent survival (*φ*), apparent recruitment/immigration (*γ*), and detection probability (*ρ*) for juvenile (calves and yearlings) and adult moose ( *Alces alces* ) in Vermont and New Hampshire USA (2014–2019). Yearling abundance and immigration are latent or unobserved states that were estimated due to the delay of reproduction of moose until the third year of life.

Parameter	Mean	SD	Lower	Upper
Calf *λ*	0.063	0.057	0.002	0.21
Yearling *λ*	0.138	0.110	0.006	0.425
Adult *λ*	0.448	0.085	0.291	0.625
Calf *φ*	0.253	0.047	0.202	0.372
Adult *φ*	0.845	0.046	0.751	0.929
Calf *γ*	0.639	0.145	0.419	0.998
Yearling *γ*	0.009	0.008	0.000	0.028
Adult *γ*	0.104	0.023	0.062	0.152
Calf *ρ*	0.181	0.035	0.114	0.253
Adult *ρ*	0.383	0.027	0.331	0.44

## 4 Discussion

We highlight how multistate DM models can be used to estimate site‐level abundance, apparent survival and recruitment, and annual population size for unmarked juvenile and adult animals captured on cameras while accounting for low and imperfect detection. Similar to Zipkin, Thorson, et al. (2014), we found that sample size (sites, surveys, and years) affected the accuracy and precision of the estimates, and this was most noticeable for apparent survival and recruitment/immigration. Further, simulations with 50% missing data also impacted inference and population trajectories. Our simulation results and empirical study indicate that this modeling approach may provide an accurate and low‐cost alternative to labor intensive telemetry and mark‐recapture field studies. Although camera surveys are data intensive (Moll et al. 2022; Steenweg et al. 2017), which has its own set of challenges (e.g., Clarfeld et al. 2023), they can provide abundant data on species whose demographic characteristics (female vs. male; juvenile vs. adult) are identifiable in photos and can be implemented at much larger spatial scales than traditional sampling approaches.

The effects of low sample size and missing data reduced the ability of models to recover known parameters, especially for apparent survival and recruitment, and this was most pronounced with fewer sites, when 50% of the data were missing, and at high abundance levels. Previous research has demonstrated that bias is more common with low sample sizes (Bellier, Kéry, and Schaub 2016; Zipkin, Thorson, et al. 2014) and that N‐mixture models can perform poorly when populations are more abundant (Madsen and Royle 2023). Our simulations to understand the impact of missing data, a common property of camera data, underscore the importance of reducing the amount of missing data in their analyses (e.g., Tucker et al. 2021). This can be accomplished in several ways such as only including years that have higher sample sizes and adopting field protocols that reduce camera malfunctions, battery drainage, and theft.

Our simulations indicated that accuracy and precision improved for apparent survival and recruitment when there were ≥ 250 camera sites, 8 or more surveys per year, 5 or more years of data, and < 50% missing data. Accuracy improved for the low abundance scenarios with 40 sites when there were more surveys per year and more years of data. However, adult recruitment was highly overestimated for all the high abundance scenarios when there were only 40 sites, indicating that these sample sizes are insufficient for capturing recruitment processes for abundant populations. Further, although accuracy and precision improved at higher sample sizes, apparent recruitment of adults was still slightly biased high and variable. These trends were evident even when we considered different values for apparent survival and recruitment that we might expect for declining (e.g., setting juvenile and adult survival and at 0.25 and 0.75, respectively) or increasing (e.g., setting juvenile survival and recruitment to 0.75 and 0.5, respectively) populations. Consequently, a study design that maximizes the number of sites, surveys per year, and number of years may increase the accuracy and precision of parameter estimation and better capture the population dynamics of focal species when using multistate DM models. Researchers can also use informative priors to improve accuracy (as we did for calf survival and detection probability for our empirical analysis) if they have a solid understanding of the biology and detection process of their focal species. Further, the effects of priors can be evaluated (e.g., we chose a uniform distribution, but a beta distribution could have been used as a prior on juvenile survival) to evaluate their impacts on estimates and uncertainty (Northrup and Gerber 2018).

Our empirical analysis of calf and adult moose data from cameras using multistate DM models provided similar inference to independent data sources in the region. First, the latitudinal gradient in site abundance was consistent with statewide density estimates that indicate moose abundance and density is higher in northern than southern Vermont and New Hampshire due to a higher availability of early successional habitats (Wattles and DeStefano 2011). Our estimates of apparent survival were high for adult moose (*φ* = 0.85; 0.75–0.93 95% CI) and similar to survival estimates obtained from radio‐collared adult moose during the same period in Vermont (*φ* = 0.88; Debow et al. 2021) and New Hampshire (*φ* = 0.82; Ellingwood et al. 2020). Calf survival, was lower on average compared to adults (*φ* = 0.25; 0.20–0.37 95% CI), which is common for moose and other ungulates (Gaillard 2007), but also slightly lower than the average rate estimated in Vermont (*φ* = 0.49; Debow et al. 2021) and New Hampshire (*φ* = 0.39; Ellingwood et al. 2020). The lower estimates of calf survival were likely due to our sampling calendar (May–August) that included all observed neonate calves (0–4 months old), which is different than previous studies that focused on calves that were 9–12 months old (January–April) (Debow et al. 2021; Jones et al. 2019). Consequently, our estimates would be lower because we accounted for the survival of animals that lived through the neonate and winter periods. Our camera network also included a larger area compared to the local telemetry studies in both states (see Figure 5) and might reflect lower than average juvenile survival rates across the region. Alternatively, apparent juvenile survival rates were often underestimated during our simulations at sample sizes comparable to our empirical data (see Figure 1, Figures S9–S10), highlighting a potential bias that arises from lower sample sizes and/or missing data. Indeed, 45% of the data were missing from our empirical study, which could also explain the lower‐than‐expected apparent juvenile survival rates.

The apparent recruitment rate of calves (*γ* = 0.64; 0.42–0.99 95% CI) was higher yet similar to that estimated in Vermont (*γ* = 0.57; Debow et al. 2022) and New Hampshire (*γ* = 0.56; Jones et al. 2017), highlighting that cameras were tracking trends in moose productivity across the region. The higher and variable recruitment rates could be attributed to missing data (45%), which is close to the 50% level that resulted in biased and high estimates for the simulation study (Figure S16). Our estimates of calf: adult cow ratios were also comparable to those measured in Colorado using intensive ground observations of telemetered moose (Bergman et al. 2020). Contrastingly, the number of new yearlings and adults immigrating into the population was low compared to calf recruitment. This finding is logical as calf survival was low, indicating fewer animals would be available to disperse to new territories than those born into a population (Debow et al. 2022; Jones et al. 2017). Further, studies in New Hampshire found that dispersal distances were relatively short (Dunfey‐Ball 2017), and similar to moose in other parts of their range (e.g., Sweden; Cederlund and Sand 1992), indicating that moose often remained in their natal regions as adults. Also, the higher immigration rates for adults versus yearlings could be interpreted as a delayed establishment of adult home ranges that often occurs after 2 years of age (Dunfey‐Ball 2017). However, we grouped yearlings with adults during the labeling process as it is difficult to differentiate between these life states from pictures. Consequently, it is unclear if yearlings or adults contributed to higher immigration rates. Combined, these results provide support for using cameras to monitor moose populations across broader spatial extents—a feat that is prohibitive using labor intensive and costly telemetry studies (Moll et al. 2022).

Our simulation approach can be amended to explore a variety of scenarios to understand the ability of multistate DMs to recover population parameters from camera studies. However, there are several considerations worth noting. First, the interpretation of abundance using site‐structured survey designs is not straightforward and inference can be limited at the site scale, making prediction uncertain (Gilbert et al. 2020). The use of stratified random designs that account for multiscale resource selection and the unique spatial ecology of focal species may provide a solution to this problem, so we plan to explore this in future analyses. Also, the aggregation of data can lead to double‐counting of individuals and so further improvements of the multistate DM could consider including terms on the count likelihood that account for false positives (e.g., Clement, Royle, and Mixan 2022). We avoided this problem by only considering the maximum number of calves and adult cows detected within pictures at a site during a monthly survey occasion, similar to Evans et al. (2024). Next, our simulation and empirical analyses did not consider any exogenous (e.g., climate) or endogenous (e.g., density‐dependence) covariates on initial abundance, apparent survival and recruitment, and detection probability. Relatedly, hyperparameters can be used to obtain subregion (e.g., cameras within a specific wildlife management district) estimates of demographic parameters/rates. These are all possible extensions that can be evaluated to understand the drivers of population dynamics and growth rates (e.g., Kanno et al. 2015). However, fitting covariates greatly increases the processing time for DMs (Kéry and Royle 2020), so more exploration may determine how to accommodate model complexity and processing time.

Future work could evaluate thresholds related to data missingness and bias and explore the relationship between the overestimation of adult recruitment and abundant populations. Our simulation study highlighted that estimates of apparent juvenile survival and recruitment are biased low and high, respectively, for camera studies with 50% missing data, regardless of sample sizes. We found support for this trend with 45% of the data missing from our empirical evaluation of moose population dynamics; however, the effects were not as exaggerated as the simulations. Therefore, there may be a threshold within the 25%–50% range that could be explored, considering that missing data is such a common feature of camera studies. Lastly, it is unclear why adult recruitment was overestimated for the simulations of abundant populations. Our choice of initial site abundance may be unrealistically high (juvenile *λ* = 10, adult *λ* = 20), so more exploration of this problem, including lower yet still relatively high site abundances, could be useful to understand this issue. To facilitate future studies, we have provided code for simulating and analyzing multistate DM data (see Sirén et al. 2024). Future work could also evaluate more demographic states. We did not include adult males (bulls) in our empirical analysis as we were interested in comparing estimates of productivity with the telemetry studies. However, we analyzed a dataset that included counts of adult bulls and cows and found negligible differences between the results of this study. These data are included online (Sirén et al. 2024) and could be analyzed using an adaptation of a four‐state DM model (see Zipkin, Sillett, et al. 2014) that also accounts for the latent yearling stage.

There are many large‐scale camera networks across the globe that have been operating for at least a decade (Steenweg et al. 2017). These camera networks provide ample opportunities to explore model complexity and continued development of multistate DMs (see Section 4 in previous paragraphs) and improve sampling and analytical approaches for estimating abundance from site‐structured data. Further, multistate count data from cameras can also be combined with other data sources (e.g., harvest data, and aerial counts) and analyzed with statistical population reconstruction (Severud et al. 2022) and integrated population models (IPMs; Schaub and Kéry 2022). The multistate DM can integrate information from other data sources (e.g., we used an informative prior on juvenile moose survival that was based on local telemetry studies) and also serve as a foundation for an IPM (Schaub and Kéry 2022). Our study highlights that camera networks can be used independently (using multistate DMs) or as a unifying data source for IPMs to aid in the monitoring and management of animals at broad spatial scales.

## Author Contributions

**Alexej P. K. Sirén:** conceptualization (lead), data curation (lead), formal analysis (lead), funding acquisition (equal), investigation (lead), methodology (lead), project administration (equal), resources (equal), software (equal), supervision (lead), validation (equal), visualization (equal), writing – original draft (lead), writing – review and editing (lead). **Michael T. Hallworth:** formal analysis (supporting), investigation (supporting), methodology (supporting), software (supporting), writing – original draft (supporting), writing – review and editing (supporting). **Jillian R. Kilborn:** funding acquisition (equal), investigation (supporting), project administration (equal), resources (equal), writing – review and editing (supporting). **Chris A. Bernier:** data curation (supporting), funding acquisition (supporting), investigation (supporting), project administration (supporting), resources (supporting), supervision (supporting), writing – review and editing (supporting). **Nicholas L. Fortin:** data curation (supporting), project administration (supporting), resources (supporting), writing – review and editing (supporting). **Katherina D. Geider:** data curation (supporting), funding acquisition (supporting), project administration (supporting), resources (supporting), writing – review and editing (supporting). **Riley K. Patry:** funding acquisition (supporting), project administration (supporting), resources (supporting), writing – review and editing (supporting). **Rachel M. Cliché:** data curation (supporting), funding acquisition (supporting), project administration (supporting), resources (supporting), writing – review and editing (supporting). **Leighlan S. Prout:** funding acquisition (supporting), investigation (supporting), project administration (supporting), resources (supporting), writing – review and editing (supporting). **Suzanne J. Gifford:** funding acquisition (supporting), project administration (supporting), resources (supporting), writing – review and editing (supporting). **Scott Wixsom:** funding acquisition (supporting), project administration (supporting), resources (supporting), supervision (supporting), writing – review and editing (supporting). **Toni Lyn Morelli:** funding acquisition (supporting), project administration (supporting), resources (supporting), supervision (supporting), writing – original draft (supporting), writing – review and editing (supporting). **Tammy L. Wilson:** formal analysis (supporting), funding acquisition (equal), methodology (supporting), project administration (supporting), resources (supporting), writing – original draft (supporting), writing – review and editing (supporting).

## Conflicts of Interest

The authors declare no conflicts of interest.

## Supporting information


Appendix S1.

## Data Availability

Data and code included in the manuscript are publicly available in Dryad under the following link: Sirén et al. 2024 (https://doi.org/10.5061/dryad.tqjq2bw76).
