# Supplementary material for: Monitoring Animal Populations With Cameras Using Open, Multistate, N‐Mixture Models
Source: Ecol Evol. 2024 Dec 12;14(12):e70583. doi: 10.1002/ece3.70583 (PMC11638134; doi:10.1002/ece3.70583)
Supplement: Supplementary file 1 — Appendix S1. [file ECE3-14-e70583-s001.pdf]

Supporting Information for: Monitoring animal populations with cameras using open, multistate, N-mixture models

Alexej P.K. Sirén[1*], Michael T. Hallworth[2], Jillian R. Kilborn[3,4], Chris A. Bernier[3], Nicholas L. Fortin [3], Katherina D. Geider[3], Riley K. Patry[5], Rachel M. Cliché[6], Leighlan S. Prout[7], Suzanne J. Gifford[8], Scott Wixsom[8], Toni Lyn Morelli[9,1], Tammy L. Wilson[10,1]

[1] Department of Environmental Conservation, University of Massachusetts, Amherst, MA, USA
[2] Vermont Center for Ecostudies, Norwich, VT, USA
[3] Vermont Department of Fish and Wildlife, Rutland, VT, USA
[4] New Hampshire Fish and Game Department, Concord, NH, USA
[5] Dartmouth College Woodlands, Milan, NH, USA
[6] United States Fish and Wildlife Service, Silvio O. Conte National Wildlife Refuge, Nulhegan Basin Division, Brunswick, VT, USA
[7] United States Forest Service, White Mountain National Forest, Campton, NH, USA
[8] United States Forest Service, Green Mountain National Forest, Mendon, VT, USA
[9] U.S. Geological Survey, Northeast Climate Adaptation Science Center, Amherst, MA, USA
[10] U.S. Geological Survey, Massachusetts Cooperative Fish and Wildlife Research Unit, Amherst, MA, USA

* Corresponding Author: Alexej P.K. Sirén, Earth Systems Research Center, 8 College Road, University of New Hampshire, Durham NH 03824; alexej.siren@unh.edu

Any use of trade, firm, or product names is for descriptive purposes only and does not imply endorsement by the U.S. Government.

Table S1. Simulated mean, lower, and upper 95% credible intervals (CI) of parameters (initial abundance = λ, apparent survival = φ, recruitment = γ, detection probability = ρ) from 2 states (juvenile and adult) estimated using a multistate Dail-Madsen (DM) model under 18 different scenarios, assuming low initial abundance and detection probability and no missing data. The ability of each model to recover the known or true parameters (Truth) was determined by assessing if the true value was within the CIs (Yes/No).

Model Scenario	State	Parameter	Truth	Mean	Lower CI	Upper CI	Within CI
nsites = 40 nyears = 2 nsurveys = 4	Juvenile	λ	1.00	2.73	0.54	25.00	Yes
nsites = 40 nyears = 2 nsurveys = 4	Adult	λ	2.00	1.73	1.05	2.93	Yes
nsites = 40 nyears = 2 nsurveys = 4	Juvenile	φ	0.50	0.47	0.01	0.96	Yes
nsites = 40 nyears = 2 nsurveys = 4	Adult	φ	0.90	0.68	0.21	0.98	Yes
nsites = 40 nyears = 2 nsurveys = 4	Juvenile	γ	0.25	1.23	0.14	10.68	Yes
nsites = 40 nyears = 2 nsurveys = 4	Adult	γ	0.01	0.66	0.03	1.84	No
nsites = 40 nyears = 2 nsurveys = 4	Juvenile	ρ	0.20	0.15	0.01	0.30	Yes
nsites = 40 nyears = 2 nsurveys = 4	Adult	ρ	0.20	0.20	0.11	0.29	Yes
nsites = 250 nyears = 2 nsurveys = 4	Juvenile	λ	1.00	0.74	0.57	0.96	No
nsites = 250 nyears = 2 nsurveys = 4	Adult	λ	2.00	1.86	1.53	2.28	Yes
nsites = 250 nyears = 2 nsurveys = 4	Juvenile	φ	0.50	0.47	0.20	0.76	Yes
nsites = 250 nyears = 2 nsurveys = 4	Adult	φ	0.90	0.88	0.73	0.99	Yes
nsites = 250 nyears = 2 nsurveys = 4	Juvenile	γ	0.25	0.23	0.16	0.32	Yes
nsites = 250 nyears = 2 nsurveys = 4	Adult	γ	0.01	0.10	0.00	0.31	Yes
nsites = 250 nyears = 2 nsurveys = 4	Juvenile	ρ	0.20	0.23	0.18	0.28	Yes
nsites = 250 nyears = 2 nsurveys = 4	Adult	ρ	0.20	0.20	0.17	0.24	Yes
nsites = 40 nyears = 5 nsurveys = 4	Juvenile	λ	1.00	1.12	0.49	2.64	Yes
nsites = 40 nyears = 5 nsurveys = 4	Adult	λ	2.00	1.93	1.35	2.63	Yes
nsites = 40 nyears = 5 nsurveys = 4	Juvenile	φ	0.50	0.17	0.01	0.44	No
nsites = 40 nyears = 5 nsurveys = 4	Adult	φ	0.90	0.96	0.87	1.00	Yes
nsites = 40 nyears = 5 nsurveys = 4	Juvenile	γ	0.25	0.52	0.26	1.18	No
nsites = 40 nyears = 5 nsurveys = 4	Adult	γ	0.01	0.08	0.00	0.25	Yes
nsites = 40 nyears = 5 nsurveys = 4	Juvenile	ρ	0.20	0.14	0.05	0.22	Yes
nsites = 40 nyears = 5 nsurveys = 4	Adult	ρ	0.20	0.22	0.17	0.27	Yes
nsites = 250 nyears = 5 nsurveys = 4	Juvenile	λ	1.00	1.22	0.99	1.50	Yes
nsites = 250 nyears = 5 nsurveys = 4	Adult	λ	2.00	1.95	1.68	2.24	Yes
nsites = 250 nyears = 5 nsurveys = 4	Juvenile	φ	0.50	0.40	0.27	0.56	Yes
nsites = 250 nyears = 5 nsurveys = 4	Adult	φ	0.90	0.92	0.87	0.96	Yes

nsites = 250 nyears = 5 nsurveys = 4	Juvenile	γ	0.25	0.27	0.22	0.33	Yes
nsites = 250 nyears = 5 nsurveys = 4	Adult	γ	0.01	0.03	0.00	0.08	Yes
nsites = 250 nyears = 5 nsurveys = 4	Juvenile	ρ	0.20	0.19	0.16	0.23	Yes
nsites = 250 nyears = 5 nsurveys = 4	Adult	ρ	0.20	0.21	0.18	0.23	Yes
nsites = 40 nyears = 10 nsurveys = 4	Juvenile	λ	1.00	0.93	0.48	1.66	Yes
nsites = 40 nyears = 10 nsurveys = 4	Adult	λ	2.00	3.06	2.11	4.29	No
nsites = 40 nyears = 10 nsurveys = 4	Juvenile	φ	0.50	0.57	0.27	0.90	Yes
nsites = 40 nyears = 10 nsurveys = 4	Adult	φ	0.90	0.86	0.76	0.92	Yes
nsites = 40 nyears = 10 nsurveys = 4	Juvenile	γ	0.25	0.23	0.15	0.34	Yes
nsites = 40 nyears = 10 nsurveys = 4	Adult	γ	0.01	0.16	0.01	0.40	No
nsites = 40 nyears = 10 nsurveys = 4	Juvenile	ρ	0.20	0.16	0.10	0.22	Yes
nsites = 40 nyears = 10 nsurveys = 4	Adult	ρ	0.20	0.14	0.11	0.18	No
nsites = 250 nyears = 10 nsurveys = 4	Juvenile	λ	1.00	0.94	0.76	1.14	Yes
nsites = 250 nyears = 10 nsurveys = 4	Adult	λ	2.00	1.98	1.74	2.22	Yes
nsites = 250 nyears = 10 nsurveys = 4	Juvenile	φ	0.50	0.56	0.45	0.67	Yes
nsites = 250 nyears = 10 nsurveys = 4	Adult	φ	0.90	0.89	0.86	0.92	Yes
nsites = 250 nyears = 10 nsurveys = 4	Juvenile	γ	0.25	0.26	0.23	0.30	Yes
nsites = 250 nyears = 10 nsurveys = 4	Adult	γ	0.01	0.01	0.00	0.03	Yes
nsites = 250 nyears = 10 nsurveys = 4	Juvenile	ρ	0.20	0.19	0.17	0.21	Yes
nsites = 250 nyears = 10 nsurveys = 4	Adult	ρ	0.20	0.20	0.18	0.21	Yes
nsites = 40 nyears = 2 nsurveys = 8	Juvenile	λ	1.00	0.97	0.60	1.49	Yes
nsites = 40 nyears = 2 nsurveys = 8	Adult	λ	2.00	1.37	0.92	1.94	No
nsites = 40 nyears = 2 nsurveys = 8	Juvenile	φ	0.50	0.21	0.01	0.52	Yes
nsites = 40 nyears = 2 nsurveys = 8	Adult	φ	0.90	0.89	0.68	1.00	Yes
nsites = 40 nyears = 2 nsurveys = 8	Juvenile	γ	0.25	0.30	0.14	0.54	Yes
nsites = 40 nyears = 2 nsurveys = 8	Adult	γ	0.01	0.32	0.02	0.78	No
nsites = 40 nyears = 2 nsurveys = 8	Juvenile	ρ	0.20	0.20	0.14	0.27	Yes
nsites = 40 nyears = 2 nsurveys = 8	Adult	ρ	0.20	0.25	0.19	0.31	Yes
nsites = 250 nyears = 2 nsurveys = 8	Juvenile	λ	1.00	1.10	0.92	1.31	Yes
nsites = 250 nyears = 2 nsurveys = 8	Adult	λ	2.00	1.97	1.71	2.27	Yes
nsites = 250 nyears = 2 nsurveys = 8	Juvenile	φ	0.50	0.40	0.24	0.57	Yes
nsites = 250 nyears = 2 nsurveys = 8	Adult	φ	0.90	0.89	0.79	0.97	Yes
nsites = 250 nyears = 2 nsurveys = 8	Juvenile	γ	0.25	0.30	0.23	0.38	Yes
nsites = 250 nyears = 2 nsurveys = 8	Adult	γ	0.01	0.07	0.00	0.23	Yes
nsites = 250 nyears = 2 nsurveys = 8	Juvenile	ρ	0.20	0.19	0.16	0.21	Yes
nsites = 250 nyears = 2 nsurveys = 8	Adult	ρ	0.20	0.21	0.18	0.23	Yes
nsites = 40 nyears = 5 nsurveys = 8	Juvenile	λ	1.00	0.78	0.47	1.15	Yes
nsites = 40 nyears = 5 nsurveys = 8	Adult	λ	2.00	2.04	1.49	2.68	Yes
nsites = 40 nyears = 5 nsurveys = 8	Juvenile	φ	0.50	0.18	0.01	0.43	No
nsites = 40 nyears = 5 nsurveys = 8	Adult	φ	0.90	0.92	0.84	0.98	Yes
nsites = 40 nyears = 5 nsurveys = 8	Juvenile	γ	0.25	0.27	0.19	0.38	Yes

nsites = 40 nyears = 5 nsurveys = 8	Adult	γ	0.01	0.17	0.04	0.34	No
nsites = 40 nyears = 5 nsurveys = 8	Juvenile	ρ	0.20	0.21	0.16	0.25	Yes
nsites = 40 nyears = 5 nsurveys = 8	Adult	ρ	0.20	0.19	0.16	0.23	Yes
nsites = 250 nyears = 5 nsurveys = 8	Juvenile	λ	1.00	0.85	0.71	0.99	No
nsites = 250 nyears = 5 nsurveys = 8	Adult	λ	2.00	2.07	1.84	2.31	Yes
nsites = 250 nyears = 5 nsurveys = 8	Juvenile	φ	0.50	0.59	0.48	0.71	Yes
nsites = 250 nyears = 5 nsurveys = 8	Adult	φ	0.90	0.90	0.86	0.93	Yes
nsites = 250 nyears = 5 nsurveys = 8	Juvenile	γ	0.25	0.27	0.24	0.30	Yes
nsites = 250 nyears = 5 nsurveys = 8	Adult	γ	0.01	0.01	0.00	0.05	Yes
nsites = 250 nyears = 5 nsurveys = 8	Juvenile	ρ	0.20	0.21	0.19	0.23	Yes
nsites = 250 nyears = 5 nsurveys = 8	Adult	ρ	0.20	0.20	0.19	0.22	Yes
nsites = 40 nyears = 10 nsurveys = 8	Juvenile	λ	1.00	1.13	0.76	1.56	Yes
nsites = 40 nyears = 10 nsurveys = 8	Adult	λ	2.00	2.36	1.81	3.03	Yes
nsites = 40 nyears = 10 nsurveys = 8	Juvenile	φ	0.50	0.48	0.30	0.68	Yes
nsites = 40 nyears = 10 nsurveys = 8	Adult	φ	0.90	0.90	0.85	0.94	Yes
nsites = 40 nyears = 10 nsurveys = 8	Juvenile	γ	0.25	0.25	0.19	0.31	Yes
nsites = 40 nyears = 10 nsurveys = 8	Adult	γ	0.01	0.03	0.00	0.09	Yes
nsites = 40 nyears = 10 nsurveys = 8	Juvenile	ρ	0.20	0.20	0.17	0.23	Yes
nsites = 40 nyears = 10 nsurveys = 8	Adult	ρ	0.20	0.19	0.16	0.22	Yes
nsites = 250 nyears = 10 nsurveys = 8	Juvenile	λ	1.00	1.08	0.92	1.25	Yes
nsites = 250 nyears = 10 nsurveys = 8	Adult	λ	2.00	2.05	1.83	2.28	Yes
nsites = 250 nyears = 10 nsurveys = 8	Juvenile	φ	0.50	0.47	0.40	0.56	Yes
nsites = 250 nyears = 10 nsurveys = 8	Adult	φ	0.90	0.90	0.88	0.92	Yes
nsites = 250 nyears = 10 nsurveys = 8	Juvenile	γ	0.25	0.24	0.22	0.26	Yes
nsites = 250 nyears = 10 nsurveys = 8	Adult	γ	0.01	0.01	0.00	0.03	Yes
nsites = 250 nyears = 10 nsurveys = 8	Juvenile	ρ	0.20	0.20	0.19	0.21	Yes
nsites = 250 nyears = 10 nsurveys = 8	Adult	ρ	0.20	0.19	0.18	0.20	Yes
nsites = 40 nyears = 2 nsurveys = 12	Juvenile	λ	1.00	1.17	0.81	1.59	Yes
nsites = 40 nyears = 2 nsurveys = 12	Adult	λ	2.00	2.22	1.53	3.12	Yes
nsites = 40 nyears = 2 nsurveys = 12	Juvenile	φ	0.50	0.69	0.25	0.98	Yes
nsites = 40 nyears = 2 nsurveys = 12	Adult	φ	0.90	0.79	0.46	0.99	Yes
nsites = 40 nyears = 2 nsurveys = 12	Juvenile	γ	0.25	0.20	0.10	0.35	Yes
nsites = 40 nyears = 2 nsurveys = 12	Adult	γ	0.01	0.51	0.03	1.59	No
nsites = 40 nyears = 2 nsurveys = 12	Juvenile	ρ	0.20	0.23	0.18	0.28	Yes
nsites = 40 nyears = 2 nsurveys = 12	Adult	ρ	0.20	0.16	0.11	0.20	Yes
nsites = 250 nyears = 2 nsurveys = 12	Juvenile	λ	1.00	0.88	0.75	1.02	Yes
nsites = 250 nyears = 2 nsurveys = 12	Adult	λ	2.00	2.01	1.78	2.28	Yes
nsites = 250 nyears = 2 nsurveys = 12	Juvenile	φ	0.50	0.37	0.21	0.52	Yes
nsites = 250 nyears = 2 nsurveys = 12	Adult	φ	0.90	0.85	0.77	0.92	Yes
nsites = 250 nyears = 2 nsurveys = 12	Juvenile	γ	0.25	0.22	0.17	0.27	Yes
nsites = 250 nyears = 2 nsurveys = 12	Adult	γ	0.01	0.07	0.00	0.20	Yes

nsites = 250 nyears = 2 nsurveys = 12	Juvenile	ρ	0.20	0.20	0.18	0.22	Yes
nsites = 250 nyears = 2 nsurveys = 12	Adult	ρ	0.20	0.20	0.18	0.22	Yes
nsites = 40 nyears = 5 nsurveys = 12	Juvenile	λ	1.00	1.03	0.72	1.40	Yes
nsites = 40 nyears = 5 nsurveys = 12	Adult	λ	2.00	1.75	1.28	2.30	Yes
nsites = 40 nyears = 5 nsurveys = 12	Juvenile	φ	0.50	0.55	0.33	0.79	Yes
nsites = 40 nyears = 5 nsurveys = 12	Adult	φ	0.90	0.84	0.76	0.91	Yes
nsites = 40 nyears = 5 nsurveys = 12	Juvenile	γ	0.25	0.28	0.21	0.37	Yes
nsites = 40 nyears = 5 nsurveys = 12	Adult	γ	0.01	0.07	0.00	0.19	Yes
nsites = 40 nyears = 5 nsurveys = 12	Juvenile	ρ	0.20	0.20	0.16	0.23	Yes
nsites = 40 nyears = 5 nsurveys = 12	Adult	ρ	0.20	0.20	0.17	0.22	Yes
nsites = 250 nyears = 5 nsurveys = 12	Juvenile	λ	1.00	0.95	0.81	1.09	Yes
nsites = 250 nyears = 5 nsurveys = 12	Adult	λ	2.00	2.06	1.86	2.29	Yes
nsites = 250 nyears = 5 nsurveys = 12	Juvenile	φ	0.50	0.57	0.48	0.67	Yes
nsites = 250 nyears = 5 nsurveys = 12	Adult	φ	0.90	0.88	0.86	0.91	Yes
nsites = 250 nyears = 5 nsurveys = 12	Juvenile	γ	0.25	0.26	0.23	0.28	Yes
nsites = 250 nyears = 5 nsurveys = 12	Adult	γ	0.01	0.04	0.01	0.08	Yes
nsites = 250 nyears = 5 nsurveys = 12	Juvenile	ρ	0.20	0.20	0.19	0.21	Yes
nsites = 250 nyears = 5 nsurveys = 12	Adult	ρ	0.20	0.20	0.19	0.21	Yes
nsites = 40 nyears = 10 nsurveys = 12	Juvenile	λ	1.00	1.00	0.68	1.37	Yes
nsites = 40 nyears = 10 nsurveys = 12	Adult	λ	2.00	2.01	1.51	2.57	Yes
nsites = 40 nyears = 10 nsurveys = 12	Juvenile	φ	0.50	0.56	0.39	0.76	Yes
nsites = 40 nyears = 10 nsurveys = 12	Adult	φ	0.90	0.90	0.86	0.94	Yes
nsites = 40 nyears = 10 nsurveys = 12	Juvenile	γ	0.25	0.24	0.20	0.28	Yes
nsites = 40 nyears = 10 nsurveys = 12	Adult	γ	0.01	0.03	0.00	0.08	Yes
nsites = 40 nyears = 10 nsurveys = 12	Juvenile	ρ	0.20	0.21	0.19	0.23	Yes
nsites = 40 nyears = 10 nsurveys = 12	Adult	ρ	0.20	0.18	0.16	0.20	Yes
nsites = 250 nyears = 10 nsurveys = 12	Juvenile	λ	1.00	1.04	0.92	1.19	Yes
nsites = 250 nyears = 10 nsurveys = 12	Adult	λ	2.00	2.06	1.86	2.27	Yes
nsites = 250 nyears = 10 nsurveys = 12	Juvenile	φ	0.50	0.52	0.45	0.59	Yes
nsites = 250 nyears = 10 nsurveys = 12	Adult	φ	0.90	0.89	0.88	0.91	Yes
nsites = 250 nyears = 10 nsurveys = 12	Juvenile	γ	0.25	0.24	0.23	0.26	Yes
nsites = 250 nyears = 10 nsurveys = 12	Adult	γ	0.01	0.02	0.00	0.03	Yes
nsites = 250 nyears = 10 nsurveys = 12	Juvenile	ρ	0.20	0.20	0.19	0.21	Yes
nsites = 250 nyears = 10 nsurveys = 12	Adult	ρ	0.20	0.19	0.19	0.20	Yes

Table S2. Simulated mean, lower, and upper 95% credible intervals (CI) of parameters (initial abundance = λ, apparent survival = φ, recruitment = γ, detection probability = ρ) from 2 states (juvenile and adult) estimated using a multistate Dail-Madsen (DM) model under 18 different scenarios, assuming low initial abundance and detection probability and 25% missing data. The ability of each model to recover the known or true parameters (Truth) was determined by assessing if the true value was within the CIs (Yes/No).

Model Scenario	State	Parameter	Truth	Mean	Lower CI	Upper CI	Within CI
nsites = 40 nyears = 2 nsurveys = 4	Juvenile	λ	1.00	3.26	0.60	19.75	Yes
nsites = 40 nyears = 2 nsurveys = 4	Adult	λ	2.00	4.87	1.78	13.90	Yes
nsites = 40 nyears = 2 nsurveys = 4	Juvenile	φ	0.50	0.33	0.01	0.92	Yes
nsites = 40 nyears = 2 nsurveys = 4	Adult	φ	0.90	0.66	0.14	0.98	Yes
nsites = 40 nyears = 2 nsurveys = 4	Juvenile	γ	0.25	0.49	0.04	3.04	Yes
nsites = 40 nyears = 2 nsurveys = 4	Adult	γ	0.01	1.61	0.04	7.16	No
nsites = 40 nyears = 2 nsurveys = 4	Juvenile	ρ	0.20	0.14	0.01	0.33	Yes
nsites = 40 nyears = 2 nsurveys = 4	Adult	ρ	0.20	0.10	0.03	0.20	Yes
nsites = 250 nyears = 2 nsurveys = 4	Juvenile	λ	1.00	1.65	1.06	2.88	No
nsites = 250 nyears = 2 nsurveys = 4	Adult	λ	2.00	1.80	1.43	2.31	Yes
nsites = 250 nyears = 2 nsurveys = 4	Juvenile	φ	0.50	0.27	0.05	0.53	Yes
nsites = 250 nyears = 2 nsurveys = 4	Adult	φ	0.90	0.71	0.48	0.92	Yes
nsites = 250 nyears = 2 nsurveys = 4	Juvenile	γ	0.25	0.45	0.26	0.81	No
nsites = 250 nyears = 2 nsurveys = 4	Adult	γ	0.01	0.37	0.02	0.91	No
nsites = 250 nyears = 2 nsurveys = 4	Juvenile	ρ	0.20	0.14	0.08	0.21	Yes
nsites = 250 nyears = 2 nsurveys = 4	Adult	ρ	0.20	0.23	0.18	0.28	Yes
nsites = 40 nyears = 5 nsurveys = 4	Juvenile	λ	1.00	1.67	0.83	3.56	Yes
nsites = 40 nyears = 5 nsurveys = 4	Adult	λ	2.00	1.68	1.16	2.38	Yes
nsites = 40 nyears = 5 nsurveys = 4	Juvenile	φ	0.50	0.21	0.03	0.46	No
nsites = 40 nyears = 5 nsurveys = 4	Adult	φ	0.90	0.88	0.75	0.97	Yes
nsites = 40 nyears = 5 nsurveys = 4	Juvenile	γ	0.25	0.57	0.29	1.23	No
nsites = 40 nyears = 5 nsurveys = 4	Adult	γ	0.01	0.10	0.00	0.29	Yes
nsites = 40 nyears = 5 nsurveys = 4	Juvenile	ρ	0.20	0.18	0.08	0.29	Yes
nsites = 40 nyears = 5 nsurveys = 4	Adult	ρ	0.20	0.26	0.20	0.33	Yes
nsites = 250 nyears = 5 nsurveys = 4	Juvenile	λ	1.00	0.97	0.73	1.28	Yes
nsites = 250 nyears = 5 nsurveys = 4	Adult	λ	2.00	2.30	1.56	10.36	Yes
nsites = 250 nyears = 5 nsurveys = 4	Juvenile	φ	0.50	0.38	0.21	0.93	Yes
nsites = 250 nyears = 5 nsurveys = 4	Adult	φ	0.90	0.94	0.89	1.00	Yes

nsites = 250 nyears = 5 nsurveys = 4	Juvenile	γ	0.25	0.27	0.06	0.36	Yes
nsites = 250 nyears = 5 nsurveys = 4	Adult	γ	0.01	0.05	0.00	0.13	Yes
nsites = 250 nyears = 5 nsurveys = 4	Juvenile	ρ	0.20	0.18	0.14	0.23	Yes
nsites = 250 nyears = 5 nsurveys = 4	Adult	ρ	0.20	0.21	0.04	0.24	Yes
nsites = 40 nyears = 10 nsurveys = 4	Juvenile	λ	1.00	1.62	0.80	3.17	Yes
nsites = 40 nyears = 10 nsurveys = 4	Adult	λ	2.00	2.18	1.48	2.98	Yes
nsites = 40 nyears = 10 nsurveys = 4	Juvenile	φ	0.50	0.57	0.26	0.88	Yes
nsites = 40 nyears = 10 nsurveys = 4	Adult	φ	0.90	0.80	0.66	0.89	No
nsites = 40 nyears = 10 nsurveys = 4	Juvenile	γ	0.25	0.38	0.22	0.70	Yes
nsites = 40 nyears = 10 nsurveys = 4	Adult	γ	0.01	0.04	0.00	0.13	Yes
nsites = 40 nyears = 10 nsurveys = 4	Juvenile	ρ	0.20	0.13	0.06	0.20	No
nsites = 40 nyears = 10 nsurveys = 4	Adult	ρ	0.20	0.18	0.13	0.22	Yes
nsites = 250 nyears = 10 nsurveys = 4	Juvenile	λ	1.00	1.07	0.86	1.33	Yes
nsites = 250 nyears = 10 nsurveys = 4	Adult	λ	2.00	1.80	1.56	2.07	Yes
nsites = 250 nyears = 10 nsurveys = 4	Juvenile	φ	0.50	0.53	0.43	0.65	Yes
nsites = 250 nyears = 10 nsurveys = 4	Adult	φ	0.90	0.88	0.85	0.90	Yes
nsites = 250 nyears = 10 nsurveys = 4	Juvenile	γ	0.25	0.27	0.23	0.33	Yes
nsites = 250 nyears = 10 nsurveys = 4	Adult	γ	0.01	0.02	0.00	0.05	Yes
nsites = 250 nyears = 10 nsurveys = 4	Juvenile	ρ	0.20	0.19	0.16	0.22	Yes
nsites = 250 nyears = 10 nsurveys = 4	Adult	ρ	0.20	0.22	0.20	0.23	Yes
nsites = 40 nyears = 2 nsurveys = 8	Juvenile	λ	1.00	0.91	0.57	1.41	Yes
nsites = 40 nyears = 2 nsurveys = 8	Adult	λ	2.00	2.27	1.51	3.32	Yes
nsites = 40 nyears = 2 nsurveys = 8	Juvenile	φ	0.50	0.41	0.04	0.90	Yes
nsites = 40 nyears = 2 nsurveys = 8	Adult	φ	0.90	0.85	0.61	0.99	Yes
nsites = 40 nyears = 2 nsurveys = 8	Juvenile	γ	0.25	0.23	0.11	0.41	Yes
nsites = 40 nyears = 2 nsurveys = 8	Adult	γ	0.01	0.16	0.00	0.62	Yes
nsites = 40 nyears = 2 nsurveys = 8	Juvenile	ρ	0.20	0.26	0.18	0.35	Yes
nsites = 40 nyears = 2 nsurveys = 8	Adult	ρ	0.20	0.18	0.12	0.24	Yes
nsites = 250 nyears = 2 nsurveys = 8	Juvenile	λ	1.00	1.17	0.95	1.43	Yes
nsites = 250 nyears = 2 nsurveys = 8	Adult	λ	2.00	2.22	1.88	2.61	Yes
nsites = 250 nyears = 2 nsurveys = 8	Juvenile	φ	0.50	0.49	0.27	0.72	Yes
nsites = 250 nyears = 2 nsurveys = 8	Adult	φ	0.90	0.87	0.75	0.98	Yes
nsites = 250 nyears = 2 nsurveys = 8	Juvenile	γ	0.25	0.24	0.18	0.31	Yes
nsites = 250 nyears = 2 nsurveys = 8	Adult	γ	0.01	0.11	0.00	0.33	Yes
nsites = 250 nyears = 2 nsurveys = 8	Juvenile	ρ	0.20	0.19	0.16	0.22	Yes
nsites = 250 nyears = 2 nsurveys = 8	Adult	ρ	0.20	0.19	0.17	0.22	Yes
nsites = 40 nyears = 5 nsurveys = 8	Juvenile	λ	1.00	0.98	0.61	1.48	Yes
nsites = 40 nyears = 5 nsurveys = 8	Adult	λ	2.00	2.26	1.64	3.08	Yes
nsites = 40 nyears = 5 nsurveys = 8	Juvenile	φ	0.50	0.31	0.06	0.61	Yes
nsites = 40 nyears = 5 nsurveys = 8	Adult	φ	0.90	0.89	0.78	0.97	Yes
nsites = 40 nyears = 5 nsurveys = 8	Juvenile	γ	0.25	0.24	0.16	0.35	Yes

nsites = 40 nyears = 5 nsurveys = 8	Adult	γ	0.01	0.21	0.03	0.47	No
nsites = 40 nyears = 5 nsurveys = 8	Juvenile	ρ	0.20	0.20	0.14	0.25	Yes
nsites = 40 nyears = 5 nsurveys = 8	Adult	ρ	0.20	0.20	0.15	0.24	Yes
nsites = 250 nyears = 5 nsurveys = 8	Juvenile	λ	1.00	1.06	0.88	1.26	Yes
nsites = 250 nyears = 5 nsurveys = 8	Adult	λ	2.00	3.11	1.83	16.11	Yes
nsites = 250 nyears = 5 nsurveys = 8	Juvenile	φ	0.50	0.41	0.26	0.96	Yes
nsites = 250 nyears = 5 nsurveys = 8	Adult	φ	0.90	0.92	0.88	1.00	Yes
nsites = 250 nyears = 5 nsurveys = 8	Juvenile	γ	0.25	0.28	0.04	0.35	Yes
nsites = 250 nyears = 5 nsurveys = 8	Adult	γ	0.01	0.02	0.00	0.07	Yes
nsites = 250 nyears = 5 nsurveys = 8	Juvenile	ρ	0.20	0.18	0.16	0.20	Yes
nsites = 250 nyears = 5 nsurveys = 8	Adult	ρ	0.20	0.18	0.03	0.22	Yes
nsites = 40 nyears = 10 nsurveys = 8	Juvenile	λ	1.00	0.97	0.61	1.45	Yes
nsites = 40 nyears = 10 nsurveys = 8	Adult	λ	2.00	1.91	1.38	2.54	Yes
nsites = 40 nyears = 10 nsurveys = 8	Juvenile	φ	0.50	0.39	0.19	0.64	Yes
nsites = 40 nyears = 10 nsurveys = 8	Adult	φ	0.90	0.93	0.87	0.98	Yes
nsites = 40 nyears = 10 nsurveys = 8	Juvenile	γ	0.25	0.27	0.20	0.35	Yes
nsites = 40 nyears = 10 nsurveys = 8	Adult	γ	0.01	0.04	0.00	0.11	Yes
nsites = 40 nyears = 10 nsurveys = 8	Juvenile	ρ	0.20	0.19	0.15	0.23	Yes
nsites = 40 nyears = 10 nsurveys = 8	Adult	ρ	0.20	0.18	0.15	0.21	Yes
nsites = 250 nyears = 10 nsurveys = 8	Juvenile	λ	1.00	1.07	0.91	1.25	Yes
nsites = 250 nyears = 10 nsurveys = 8	Adult	λ	2.00	2.16	1.93	2.41	Yes
nsites = 250 nyears = 10 nsurveys = 8	Juvenile	φ	0.50	0.49	0.41	0.58	Yes
nsites = 250 nyears = 10 nsurveys = 8	Adult	φ	0.90	0.89	0.87	0.91	Yes
nsites = 250 nyears = 10 nsurveys = 8	Juvenile	γ	0.25	0.25	0.23	0.28	Yes
nsites = 250 nyears = 10 nsurveys = 8	Adult	γ	0.01	0.01	0.00	0.03	Yes
nsites = 250 nyears = 10 nsurveys = 8	Juvenile	ρ	0.20	0.20	0.18	0.21	Yes
nsites = 250 nyears = 10 nsurveys = 8	Adult	ρ	0.20	0.20	0.19	0.21	Yes
nsites = 40 nyears = 2 nsurveys = 12	Juvenile	λ	1.00	1.20	0.74	1.89	Yes
nsites = 40 nyears = 2 nsurveys = 12	Adult	λ	2.00	1.99	1.42	2.72	Yes
nsites = 40 nyears = 2 nsurveys = 12	Juvenile	φ	0.50	0.30	0.03	0.66	Yes
nsites = 40 nyears = 2 nsurveys = 12	Adult	φ	0.90	0.80	0.54	0.98	Yes
nsites = 40 nyears = 2 nsurveys = 12	Juvenile	γ	0.25	0.31	0.15	0.56	Yes
nsites = 40 nyears = 2 nsurveys = 12	Adult	γ	0.01	0.35	0.01	1.05	No
nsites = 40 nyears = 2 nsurveys = 12	Juvenile	ρ	0.20	0.14	0.09	0.19	No
nsites = 40 nyears = 2 nsurveys = 12	Adult	ρ	0.20	0.22	0.16	0.26	Yes
nsites = 250 nyears = 2 nsurveys = 12	Juvenile	λ	1.00	0.95	0.81	1.13	Yes
nsites = 250 nyears = 2 nsurveys = 12	Adult	λ	2.00	2.18	1.90	2.51	Yes
nsites = 250 nyears = 2 nsurveys = 12	Juvenile	φ	0.50	0.39	0.22	0.57	Yes
nsites = 250 nyears = 2 nsurveys = 12	Adult	φ	0.90	0.91	0.81	0.98	Yes
nsites = 250 nyears = 2 nsurveys = 12	Juvenile	γ	0.25	0.21	0.16	0.27	Yes
nsites = 250 nyears = 2 nsurveys = 12	Adult	γ	0.01	0.11	0.00	0.33	Yes

nsites = 250 nyears = 2 nsurveys = 12	Juvenile	ρ	0.20	0.20	0.18	0.22	Yes
nsites = 250 nyears = 2 nsurveys = 12	Adult	ρ	0.20	0.18	0.16	0.20	Yes
nsites = 40 nyears = 5 nsurveys = 12	Juvenile	λ	1.00	1.32	0.91	1.84	Yes
nsites = 40 nyears = 5 nsurveys = 12	Adult	λ	2.00	2.43	1.81	3.17	Yes
nsites = 40 nyears = 5 nsurveys = 12	Juvenile	φ	0.50	0.43	0.23	0.66	Yes
nsites = 40 nyears = 5 nsurveys = 12	Adult	φ	0.90	0.86	0.78	0.93	Yes
nsites = 40 nyears = 5 nsurveys = 12	Juvenile	γ	0.25	0.23	0.16	0.31	Yes
nsites = 40 nyears = 5 nsurveys = 12	Adult	γ	0.01	0.05	0.00	0.17	Yes
nsites = 40 nyears = 5 nsurveys = 12	Juvenile	ρ	0.20	0.18	0.15	0.22	Yes
nsites = 40 nyears = 5 nsurveys = 12	Adult	ρ	0.20	0.18	0.15	0.21	Yes
nsites = 250 nyears = 5 nsurveys = 12	Juvenile	λ	1.00	1.00	0.86	1.15	Yes
nsites = 250 nyears = 5 nsurveys = 12	Adult	λ	2.00	2.02	1.81	2.26	Yes
nsites = 250 nyears = 5 nsurveys = 12	Juvenile	φ	0.50	0.63	0.53	0.73	No
nsites = 250 nyears = 5 nsurveys = 12	Adult	φ	0.90	0.88	0.85	0.91	Yes
nsites = 250 nyears = 5 nsurveys = 12	Juvenile	γ	0.25	0.22	0.20	0.25	No
nsites = 250 nyears = 5 nsurveys = 12	Adult	γ	0.01	0.02	0.00	0.06	Yes
nsites = 250 nyears = 5 nsurveys = 12	Juvenile	ρ	0.20	0.21	0.19	0.23	Yes
nsites = 250 nyears = 5 nsurveys = 12	Adult	ρ	0.20	0.19	0.18	0.20	Yes
nsites = 40 nyears = 10 nsurveys = 12	Juvenile	λ	1.00	1.04	0.70	1.45	Yes
nsites = 40 nyears = 10 nsurveys = 12	Adult	λ	2.00	2.14	1.63	2.75	Yes
nsites = 40 nyears = 10 nsurveys = 12	Juvenile	φ	0.50	0.37	0.21	0.54	Yes
nsites = 40 nyears = 10 nsurveys = 12	Adult	φ	0.90	0.86	0.81	0.91	Yes
nsites = 40 nyears = 10 nsurveys = 12	Juvenile	γ	0.25	0.25	0.20	0.30	Yes
nsites = 40 nyears = 10 nsurveys = 12	Adult	γ	0.01	0.06	0.01	0.14	Yes
nsites = 40 nyears = 10 nsurveys = 12	Juvenile	ρ	0.20	0.19	0.16	0.22	Yes
nsites = 40 nyears = 10 nsurveys = 12	Adult	ρ	0.20	0.20	0.18	0.23	Yes
nsites = 250 nyears = 10 nsurveys = 12	Juvenile	λ	1.00	1.03	0.89	1.19	Yes
nsites = 250 nyears = 10 nsurveys = 12	Adult	λ	2.00	1.93	1.73	2.13	Yes
nsites = 250 nyears = 10 nsurveys = 12	Juvenile	φ	0.50	0.50	0.44	0.57	Yes
nsites = 250 nyears = 10 nsurveys = 12	Adult	φ	0.90	0.91	0.89	0.92	Yes
nsites = 250 nyears = 10 nsurveys = 12	Juvenile	γ	0.25	0.25	0.23	0.27	Yes
nsites = 250 nyears = 10 nsurveys = 12	Adult	γ	0.01	0.02	0.00	0.03	Yes
nsites = 250 nyears = 10 nsurveys = 12	Juvenile	ρ	0.20	0.21	0.20	0.22	Yes
nsites = 250 nyears = 10 nsurveys = 12	Adult	ρ	0.20	0.21	0.20	0.22	Yes

Table S3. Simulated mean, lower, and upper 95% credible intervals (CI) of parameters (initial abundance = λ, apparent survival = φ, recruitment = γ, detection probability = ρ) from 2 states (juvenile and adult) estimated using a multistate Dail-Madsen (DM)s model under 18 different scenarios, assuming low initial abundance and detection probability and 50% missing data. The ability of each model to recover the known or true parameters (Truth) was determined by assessing if the true value was within the CIs (Yes/No). Note, that convergence was not achieved with some parameters for scenarios 4 and 16.

Model Scenario	State	Parameter	Truth	Mean	Lower CI	Upper CI	Within CI
nsites = 40 nyears = 2 nsurveys = 4	Juvenile	λ	1.00	2.56	0.47	19.98	Yes
nsites = 40 nyears = 2 nsurveys = 4	Adult	λ	2.00	1.69	0.86	3.49	Yes
nsites = 40 nyears = 2 nsurveys = 4	Juvenile	φ	0.50	0.24	0.01	0.76	Yes
nsites = 40 nyears = 2 nsurveys = 4	Adult	φ	0.90	0.43	0.04	0.92	Yes
nsites = 40 nyears = 2 nsurveys = 4	Juvenile	γ	0.25	1.62	0.21	11.87	Yes
nsites = 40 nyears = 2 nsurveys = 4	Adult	γ	0.01	1.03	0.12	2.64	No
nsites = 40 nyears = 2 nsurveys = 4	Juvenile	ρ	0.20	0.22	0.01	0.42	Yes
nsites = 40 nyears = 2 nsurveys = 4	Adult	ρ	0.20	0.22	0.09	0.35	Yes
nsites = 250 nyears = 2 nsurveys = 4	Juvenile	λ	1.00	1.08	0.72	1.76	Yes
nsites = 250 nyears = 2 nsurveys = 4	Adult	λ	2.00	2.00	1.52	2.64	Yes
nsites = 250 nyears = 2 nsurveys = 4	Juvenile	φ	0.50	0.17	0.01	0.49	No
nsites = 250 nyears = 2 nsurveys = 4	Adult	φ	0.90	0.85	0.62	0.99	Yes
nsites = 250 nyears = 2 nsurveys = 4	Juvenile	γ	0.25	0.62	0.36	1.08	Yes
nsites = 250 nyears = 2 nsurveys = 4	Adult	γ	0.01	0.47	0.07	1.01	No
nsites = 250 nyears = 2 nsurveys = 4	Juvenile	ρ	0.20	0.18	0.10	0.25	Yes
nsites = 250 nyears = 2 nsurveys = 4	Adult	ρ	0.20	0.19	0.14	0.24	Yes
nsites = 40 nyears = 5 nsurveys = 4	Juvenile	λ	1.00	1.46	0.69	3.10	Yes
nsites = 40 nyears = 5 nsurveys = 4	Adult	λ	2.00	1.81	1.17	2.68	Yes
nsites = 40 nyears = 5 nsurveys = 4	Juvenile	φ	0.50	0.40	0.14	0.77	Yes
nsites = 40 nyears = 5 nsurveys = 4	Adult	φ	0.90	0.77	0.54	0.94	Yes
nsites = 40 nyears = 5 nsurveys = 4	Juvenile	γ	0.25	0.79	0.40	1.59	Yes
nsites = 40 nyears = 5 nsurveys = 4	Adult	γ	0.01	0.14	0.00	0.43	Yes
nsites = 40 nyears = 5 nsurveys = 4	Juvenile	ρ	0.20	0.17	0.07	0.27	Yes
nsites = 40 nyears = 5 nsurveys = 4	Adult	ρ	0.20	0.23	0.17	0.30	Yes
nsites = 250 nyears = 5 nsurveys = 4	Juvenile	λ	1.00	0.94	0.72	1.21	Yes
nsites = 250 nyears = 5 nsurveys = 4	Adult	λ	2.00	2.66	1.80	10.77	Yes
nsites = 250 nyears = 5 nsurveys = 4	Juvenile	φ	0.50	0.50	0.33	0.98	Yes
nsites = 250 nyears = 5 nsurveys = 4	Adult	φ	0.90	0.96	0.89	1.00	Yes
nsites = 250 nyears = 5 nsurveys = 4	Juvenile	γ	0.25	0.41	0.11	0.54	Yes
nsites = 250 nyears = 5 nsurveys = 4	Adult	γ	0.01	0.03	0.00	0.09	Yes
nsites = 250 nyears = 5 nsurveys = 4	Juvenile	ρ	0.20	0.22	0.18	0.27	Yes
nsites = 250 nyears = 5 nsurveys = 4	Adult	ρ	0.20	0.20	0.04	0.23	Yes

nsites = 40 nyears = 10 nsurveys = 4	Juvenile	λ	1.00	1.10	0.60	1.93	Yes
nsites = 40 nyears = 10 nsurveys = 4	Adult	λ	2.00	1.66	1.11	2.38	Yes
nsites = 40 nyears = 10 nsurveys = 4	Juvenile	φ	0.50	0.49	0.26	0.78	Yes
nsites = 40 nyears = 10 nsurveys = 4	Adult	φ	0.90	0.89	0.78	0.96	Yes
nsites = 40 nyears = 10 nsurveys = 4	Juvenile	γ	0.25	0.65	0.43	1.04	Yes
nsites = 40 nyears = 10 nsurveys = 4	Adult	γ	0.01	0.06	0.00	0.19	Yes
nsites = 40 nyears = 10 nsurveys = 4	Juvenile	ρ	0.20	0.19	0.12	0.27	Yes
nsites = 40 nyears = 10 nsurveys = 4	Adult	ρ	0.20	0.21	0.16	0.26	Yes
nsites = 250 nyears = 10 nsurveys = 4	Juvenile	λ	1.00	1.07	0.85	1.32	Yes
nsites = 250 nyears = 10 nsurveys = 4	Adult	λ	2.00	2.04	1.77	2.33	Yes
nsites = 250 nyears = 10 nsurveys = 4	Juvenile	φ	0.50	0.62	0.49	0.76	Yes
nsites = 250 nyears = 10 nsurveys = 4	Adult	φ	0.90	0.85	0.80	0.90	No
nsites = 250 nyears = 10 nsurveys = 4	Juvenile	γ	0.25	0.48	0.41	0.57	Yes
nsites = 250 nyears = 10 nsurveys = 4	Adult	γ	0.01	0.02	0.01	0.05	Yes
nsites = 250 nyears = 10 nsurveys = 4	Juvenile	ρ	0.20	0.19	0.17	0.22	Yes
nsites = 250 nyears = 10 nsurveys = 4	Adult	ρ	0.20	0.19	0.17	0.21	Yes
nsites = 40 nyears = 2 nsurveys = 8	Juvenile	λ	1.00	2.28	0.93	6.95	Yes
nsites = 40 nyears = 2 nsurveys = 8	Adult	λ	2.00	1.89	1.18	2.97	Yes
nsites = 40 nyears = 2 nsurveys = 8	Juvenile	φ	0.50	0.40	0.02	0.92	Yes
nsites = 40 nyears = 2 nsurveys = 8	Adult	φ	0.90	0.73	0.23	0.99	Yes
nsites = 40 nyears = 2 nsurveys = 8	Juvenile	γ	0.25	0.95	0.30	3.30	Yes
nsites = 40 nyears = 2 nsurveys = 8	Adult	γ	0.01	1.07	0.08	2.71	No
nsites = 40 nyears = 2 nsurveys = 8	Juvenile	ρ	0.20	0.13	0.03	0.23	Yes
nsites = 40 nyears = 2 nsurveys = 8	Adult	ρ	0.20	0.18	0.11	0.26	Yes
nsites = 250 nyears = 2 nsurveys = 8	Juvenile	λ	1.00	1.15	0.93	1.44	Yes
nsites = 250 nyears = 2 nsurveys = 8	Adult	λ	2.00	1.72	1.46	2.01	Yes
nsites = 250 nyears = 2 nsurveys = 8	Juvenile	φ	0.50	0.55	0.35	0.77	Yes
nsites = 250 nyears = 2 nsurveys = 8	Adult	φ	0.90	0.87	0.73	0.98	Yes
nsites = 250 nyears = 2 nsurveys = 8	Juvenile	γ	0.25	0.54	0.40	0.71	Yes
nsites = 250 nyears = 2 nsurveys = 8	Adult	γ	0.01	0.10	0.00	0.30	Yes
nsites = 250 nyears = 2 nsurveys = 8	Juvenile	ρ	0.20	0.18	0.15	0.22	Yes
nsites = 250 nyears = 2 nsurveys = 8	Adult	ρ	0.20	0.21	0.18	0.24	Yes
nsites = 40 nyears = 5 nsurveys = 8	Juvenile	λ	1.00	1.15	0.73	1.69	Yes
nsites = 40 nyears = 5 nsurveys = 8	Adult	λ	2.00	2.20	1.57	2.97	Yes
nsites = 40 nyears = 5 nsurveys = 8	Juvenile	φ	0.50	0.34	0.14	0.63	Yes
nsites = 40 nyears = 5 nsurveys = 8	Adult	φ	0.90	0.90	0.75	0.99	Yes
nsites = 40 nyears = 5 nsurveys = 8	Juvenile	γ	0.25	0.50	0.35	0.72	Yes
nsites = 40 nyears = 5 nsurveys = 8	Adult	γ	0.01	0.08	0.00	0.27	Yes
nsites = 40 nyears = 5 nsurveys = 8	Juvenile	ρ	0.20	0.21	0.15	0.26	Yes
nsites = 40 nyears = 5 nsurveys = 8	Adult	ρ	0.20	0.21	0.17	0.26	Yes
nsites = 250 nyears = 5 nsurveys = 8	Juvenile	λ	1.00	1.00	0.84	1.20	Yes
nsites = 250 nyears = 5 nsurveys = 8	Adult	λ	2.00	2.47	1.66	9.59	Yes
nsites = 250 nyears = 5 nsurveys = 8	Juvenile	φ	0.50	0.57	0.43	0.99	Yes
nsites = 250 nyears = 5 nsurveys = 8	Adult	φ	0.90	0.91	0.86	1.00	Yes
nsites = 250 nyears = 5 nsurveys = 8	Juvenile	γ	0.25	0.46	0.12	0.57	Yes
nsites = 250 nyears = 5 nsurveys = 8	Adult	γ	0.01	0.02	0.00	0.06	Yes
nsites = 250 nyears = 5 nsurveys = 8	Juvenile	ρ	0.20	0.21	0.18	0.23	Yes
nsites = 250 nyears = 5 nsurveys = 8	Adult	ρ	0.20	0.20	0.05	0.23	Yes
nsites = 40 nyears = 10 nsurveys = 8	Juvenile	λ	1.00	1.03	0.66	1.54	Yes

nsites = 40 nyears = 10 nsurveys = 8	Adult	λ	2.00	2.09	1.51	2.78	Yes
nsites = 40 nyears = 10 nsurveys = 8	Juvenile	φ	0.50	0.45	0.28	0.69	Yes
nsites = 40 nyears = 10 nsurveys = 8	Adult	φ	0.90	0.93	0.83	0.99	Yes
nsites = 40 nyears = 10 nsurveys = 8	Juvenile	γ	0.25	0.55	0.42	0.73	Yes
nsites = 40 nyears = 10 nsurveys = 8	Adult	γ	0.01	0.06	0.01	0.15	Yes
nsites = 40 nyears = 10 nsurveys = 8	Juvenile	ρ	0.20	0.18	0.15	0.22	Yes
nsites = 40 nyears = 10 nsurveys = 8	Adult	ρ	0.20	0.18	0.14	0.21	Yes
nsites = 250 nyears = 10 nsurveys = 8	Juvenile	λ	1.00	0.86	0.72	1.01	Yes
nsites = 250 nyears = 10 nsurveys = 8	Adult	λ	2.00	1.96	1.74	2.20	Yes
nsites = 250 nyears = 10 nsurveys = 8	Juvenile	φ	0.50	0.58	0.50	0.66	Yes
nsites = 250 nyears = 10 nsurveys = 8	Adult	φ	0.90	0.88	0.85	0.91	Yes
nsites = 250 nyears = 10 nsurveys = 8	Juvenile	γ	0.25	0.46	0.42	0.51	Yes
nsites = 250 nyears = 10 nsurveys = 8	Adult	γ	0.01	0.02	0.00	0.05	Yes
nsites = 250 nyears = 10 nsurveys = 8	Juvenile	ρ	0.20	0.22	0.21	0.24	No
nsites = 250 nyears = 10 nsurveys = 8	Adult	ρ	0.20	0.20	0.19	0.22	Yes
nsites = 40 nyears = 2 nsurveys = 12	Juvenile	λ	1.00	1.02	0.67	1.48	Yes
nsites = 40 nyears = 2 nsurveys = 12	Adult	λ	2.00	1.97	1.41	2.66	Yes
nsites = 40 nyears = 2 nsurveys = 12	Juvenile	φ	0.50	0.39	0.07	0.73	Yes
nsites = 40 nyears = 2 nsurveys = 12	Adult	φ	0.90	0.83	0.62	0.98	Yes
nsites = 40 nyears = 2 nsurveys = 12	Juvenile	γ	0.25	0.53	0.31	0.83	Yes
nsites = 40 nyears = 2 nsurveys = 12	Adult	γ	0.01	0.20	0.01	0.72	Yes
nsites = 40 nyears = 2 nsurveys = 12	Juvenile	ρ	0.20	0.23	0.17	0.29	Yes
nsites = 40 nyears = 2 nsurveys = 12	Adult	ρ	0.20	0.25	0.19	0.30	Yes
nsites = 250 nyears = 2 nsurveys = 12	Juvenile	λ	1.00	1.07	0.90	1.24	Yes
nsites = 250 nyears = 2 nsurveys = 12	Adult	λ	2.00	2.25	1.96	2.56	Yes
nsites = 250 nyears = 2 nsurveys = 12	Juvenile	φ	0.50	0.54	0.36	0.74	Yes
nsites = 250 nyears = 2 nsurveys = 12	Adult	φ	0.90	0.88	0.77	0.97	Yes
nsites = 250 nyears = 2 nsurveys = 12	Juvenile	γ	0.25	0.47	0.38	0.57	Yes
nsites = 250 nyears = 2 nsurveys = 12	Adult	γ	0.01	0.07	0.00	0.24	Yes
nsites = 250 nyears = 2 nsurveys = 12	Juvenile	ρ	0.20	0.21	0.19	0.23	Yes
nsites = 250 nyears = 2 nsurveys = 12	Adult	ρ	0.20	0.20	0.18	0.22	Yes
nsites = 40 nyears = 5 nsurveys = 12	Juvenile	λ	1.00	0.89	0.59	1.28	Yes
nsites = 40 nyears = 5 nsurveys = 12	Adult	λ	2.00	2.11	1.54	2.81	Yes
nsites = 40 nyears = 5 nsurveys = 12	Juvenile	φ	0.50	0.50	0.26	0.78	Yes
nsites = 40 nyears = 5 nsurveys = 12	Adult	φ	0.90	0.88	0.75	0.97	Yes
nsites = 40 nyears = 5 nsurveys = 12	Juvenile	γ	0.25	0.50	0.35	0.66	Yes
nsites = 40 nyears = 5 nsurveys = 12	Adult	γ	0.01	0.10	0.00	0.30	Yes
nsites = 40 nyears = 5 nsurveys = 12	Juvenile	ρ	0.20	0.22	0.18	0.27	Yes
nsites = 40 nyears = 5 nsurveys = 12	Adult	ρ	0.20	0.19	0.15	0.23	Yes
nsites = 250 nyears = 5 nsurveys = 12	Juvenile	λ	1.00	1.14	0.98	1.32	Yes
nsites = 250 nyears = 5 nsurveys = 12	Adult	λ	2.00	2.07	1.80	2.33	Yes
nsites = 250 nyears = 5 nsurveys = 12	Juvenile	φ	0.50	0.45	0.36	0.55	Yes
nsites = 250 nyears = 5 nsurveys = 12	Adult	φ	0.90	0.89	0.85	0.93	Yes
nsites = 250 nyears = 5 nsurveys = 12	Juvenile	γ	0.25	0.51	0.45	0.57	Yes
nsites = 250 nyears = 5 nsurveys = 12	Adult	γ	0.01	0.02	0.00	0.05	Yes
nsites = 250 nyears = 5 nsurveys = 12	Juvenile	ρ	0.20	0.19	0.18	0.21	Yes
nsites = 250 nyears = 5 nsurveys = 12	Adult	ρ	0.20	0.20	0.18	0.21	Yes
nsites = 40 nyears = 10 nsurveys = 12	Juvenile	λ	1.00	1.06	0.70	1.49	Yes
nsites = 40 nyears = 10 nsurveys = 12	Adult	λ	2.00	2.50	1.91	3.16	Yes

nsites = 40 nyears = 10 nsurveys = 12	Juvenile	φ	0.50	0.48	0.34	0.66	Yes
nsites = 40 nyears = 10 nsurveys = 12	Adult	φ	0.90	0.88	0.82	0.94	Yes
nsites = 40 nyears = 10 nsurveys = 12	Juvenile	γ	0.25	0.47	0.38	0.57	Yes
nsites = 40 nyears = 10 nsurveys = 12	Adult	γ	0.01	0.04	0.00	0.11	Yes
nsites = 40 nyears = 10 nsurveys = 12	Juvenile	ρ	0.20	0.19	0.16	0.22	Yes
nsites = 40 nyears = 10 nsurveys = 12	Adult	ρ	0.20	0.18	0.16	0.21	Yes
nsites = 250 nyears = 10 nsurveys = 12	Juvenile	λ	1.00	0.87	0.73	1.02	Yes
nsites = 250 nyears = 10 nsurveys = 12	Adult	λ	2.00	2.29	2.05	2.53	No
nsites = 250 nyears = 10 nsurveys = 12	Juvenile	φ	0.50	0.49	0.43	0.56	Yes
nsites = 250 nyears = 10 nsurveys = 12	Adult	φ	0.90	0.89	0.86	0.91	Yes
nsites = 250 nyears = 10 nsurveys = 12	Juvenile	γ	0.25	0.51	0.47	0.56	Yes
nsites = 250 nyears = 10 nsurveys = 12	Adult	γ	0.01	0.03	0.01	0.05	Yes
nsites = 250 nyears = 10 nsurveys = 12	Juvenile	ρ	0.20	0.20	0.18	0.21	Yes
nsites = 250 nyears = 10 nsurveys = 12	Adult	ρ	0.20	0.20	0.19	0.21	Yes

Table S4. Simulated mean, lower, and upper 95% credible intervals (CI) of parameters (initial abundance = λ, apparent survival = φ, recruitment = γ, detection probability = ρ) from 2 states (juvenile and adult) estimated using a multistate Dail-Madsen (DM) model under 18 different scenarios, assuming high initial abundance and detection probability and no missing data. The ability of each model to recover the known or true parameters (Truth) was determined by assessing if the true value was within the CIs (Yes/No).

Model Scenario	State	Parameter	Truth	Mean	Lower CI	Upper CI	Within CI
nsites = 40 nyears = 2 nsurveys = 4	Juvenile	λ	1.00	12.73	7.80	23.42	Yes
nsites = 40 nyears = 2 nsurveys = 4	Adult	λ	2.00	21.51	12.10	39.42	Yes
nsites = 40 nyears = 2 nsurveys = 4	Juvenile	φ	0.50	0.33	0.01	0.85	Yes
nsites = 40 nyears = 2 nsurveys = 4	Adult	φ	0.90	0.64	0.16	0.97	Yes
nsites = 40 nyears = 2 nsurveys = 4	Juvenile	γ	0.25	0.25	0.10	0.53	Yes
nsites = 40 nyears = 2 nsurveys = 4	Adult	γ	0.01	7.16	0.30	24.23	No
nsites = 40 nyears = 2 nsurveys = 4	Juvenile	ρ	0.20	0.19	0.09	0.28	Yes
nsites = 40 nyears = 2 nsurveys = 4	Adult	ρ	0.20	0.20	0.10	0.33	Yes
nsites = 250 nyears = 2 nsurveys = 4	Juvenile	λ	1.00	8.00	6.71	9.68	No
nsites = 250 nyears = 2 nsurveys = 4	Adult	λ	2.00	21.80	16.94	29.38	Yes
nsites = 250 nyears = 2 nsurveys = 4	Juvenile	φ	0.50	0.71	0.34	0.97	Yes
nsites = 250 nyears = 2 nsurveys = 4	Adult	φ	0.90	0.83	0.68	0.97	Yes
nsites = 250 nyears = 2 nsurveys = 4	Juvenile	γ	0.25	0.19	0.13	0.25	Yes
nsites = 250 nyears = 2 nsurveys = 4	Adult	γ	0.01	1.02	0.03	3.86	No
nsites = 250 nyears = 2 nsurveys = 4	Juvenile	ρ	0.20	0.24	0.20	0.29	No
nsites = 250 nyears = 2 nsurveys = 4	Adult	ρ	0.20	0.19	0.14	0.23	Yes
nsites = 40 nyears = 5 nsurveys = 4	Juvenile	λ	1.00	11.50	7.82	17.79	Yes
nsites = 40 nyears = 5 nsurveys = 4	Adult	λ	2.00	22.40	15.26	33.83	Yes
nsites = 40 nyears = 5 nsurveys = 4	Juvenile	φ	0.50	0.50	0.18	0.89	Yes
nsites = 40 nyears = 5 nsurveys = 4	Adult	φ	0.90	0.81	0.65	0.91	Yes
nsites = 40 nyears = 5 nsurveys = 4	Juvenile	γ	0.25	0.25	0.15	0.43	Yes
nsites = 40 nyears = 5 nsurveys = 4	Adult	γ	0.01	1.88	0.06	5.96	No
nsites = 40 nyears = 5 nsurveys = 4	Juvenile	ρ	0.20	0.18	0.12	0.25	Yes
nsites = 40 nyears = 5 nsurveys = 4	Adult	ρ	0.20	0.19	0.12	0.26	Yes
nsites = 250 nyears = 5 nsurveys = 4	Juvenile	λ	1.00	9.75	8.44	11.31	Yes
nsites = 250 nyears = 5 nsurveys = 4	Adult	λ	2.00	21.07	18.09	25.36	Yes
nsites = 250 nyears = 5 nsurveys = 4	Juvenile	φ	0.50	0.54	0.39	0.74	Yes
nsites = 250 nyears = 5 nsurveys = 4	Adult	φ	0.90	0.89	0.84	0.92	Yes
nsites = 250 nyears = 5 nsurveys = 4	Juvenile	γ	0.25	0.25	0.20	0.30	Yes
nsites = 250 nyears = 5 nsurveys = 4	Adult	γ	0.01	0.16	0.00	0.58	Yes
nsites = 250 nyears = 5 nsurveys = 4	Juvenile	ρ	0.20	0.19	0.17	0.22	Yes
nsites = 250 nyears = 5 nsurveys = 4	Adult	ρ	0.20	0.19	0.16	0.22	Yes
nsites = 40 nyears = 10 nsurveys = 4	Juvenile	λ	1.00	10.65	8.21	13.89	Yes
nsites = 40 nyears = 10 nsurveys = 4	Adult	λ	2.00	19.44	15.14	25.77	Yes

nsites = 40 nyears = 10 nsurveys = 4	Juvenile	φ	0.50	0.35	0.18	0.59	Yes
nsites = 40 nyears = 10 nsurveys = 4	Adult	φ	0.90	0.92	0.86	0.96	Yes
nsites = 40 nyears = 10 nsurveys = 4	Juvenile	γ	0.25	0.29	0.21	0.40	Yes
nsites = 40 nyears = 10 nsurveys = 4	Adult	γ	0.01	0.32	0.01	1.00	No
nsites = 40 nyears = 10 nsurveys = 4	Juvenile	ρ	0.20	0.20	0.15	0.24	Yes
nsites = 40 nyears = 10 nsurveys = 4	Adult	ρ	0.20	0.22	0.16	0.28	Yes
nsites = 250 nyears = 10 nsurveys = 4	Juvenile	λ	1.00	9.76	8.78	10.85	Yes
nsites = 250 nyears = 10 nsurveys = 4	Adult	λ	2.00	23.33	20.80	26.67	No
nsites = 250 nyears = 10 nsurveys = 4	Juvenile	φ	0.50	0.62	0.46	0.81	Yes
nsites = 250 nyears = 10 nsurveys = 4	Adult	φ	0.90	0.89	0.86	0.91	Yes
nsites = 250 nyears = 10 nsurveys = 4	Juvenile	γ	0.25	0.22	0.19	0.25	Yes
nsites = 250 nyears = 10 nsurveys = 4	Adult	γ	0.01	0.06	0.00	0.22	Yes
nsites = 250 nyears = 10 nsurveys = 4	Juvenile	ρ	0.20	0.20	0.18	0.22	Yes
nsites = 250 nyears = 10 nsurveys = 4	Adult	ρ	0.20	0.18	0.15	0.20	No
nsites = 40 nyears = 2 nsurveys = 8	Juvenile	λ	1.00	8.69	6.31	12.23	Yes
nsites = 40 nyears = 2 nsurveys = 8	Adult	λ	2.00	14.76	11.04	20.55	Yes
nsites = 40 nyears = 2 nsurveys = 8	Juvenile	φ	0.50	0.25	0.01	0.61	Yes
nsites = 40 nyears = 2 nsurveys = 8	Adult	φ	0.90	0.79	0.49	0.97	Yes
nsites = 40 nyears = 2 nsurveys = 8	Juvenile	γ	0.25	0.28	0.17	0.44	Yes
nsites = 40 nyears = 2 nsurveys = 8	Adult	γ	0.01	2.72	0.15	7.92	No
nsites = 40 nyears = 2 nsurveys = 8	Juvenile	ρ	0.20	0.23	0.16	0.31	Yes
nsites = 40 nyears = 2 nsurveys = 8	Adult	ρ	0.20	0.27	0.19	0.34	Yes
nsites = 250 nyears = 2 nsurveys = 8	Juvenile	λ	1.00	10.38	9.05	11.95	Yes
nsites = 250 nyears = 2 nsurveys = 8	Adult	λ	2.00	22.01	18.60	26.51	Yes
nsites = 250 nyears = 2 nsurveys = 8	Juvenile	φ	0.50	0.47	0.25	0.71	Yes
nsites = 250 nyears = 2 nsurveys = 8	Adult	φ	0.90	0.85	0.72	0.96	Yes
nsites = 250 nyears = 2 nsurveys = 8	Juvenile	γ	0.25	0.21	0.17	0.27	Yes
nsites = 250 nyears = 2 nsurveys = 8	Adult	γ	0.01	1.25	0.06	3.77	No
nsites = 250 nyears = 2 nsurveys = 8	Juvenile	ρ	0.20	0.20	0.17	0.22	Yes
nsites = 250 nyears = 2 nsurveys = 8	Adult	ρ	0.20	0.19	0.16	0.23	Yes
nsites = 40 nyears = 5 nsurveys = 8	Juvenile	λ	1.00	10.02	8.11	12.35	Yes
nsites = 40 nyears = 5 nsurveys = 8	Adult	λ	2.00	25.25	18.63	33.35	Yes
nsites = 40 nyears = 5 nsurveys = 8	Juvenile	φ	0.50	0.69	0.37	0.97	Yes
nsites = 40 nyears = 5 nsurveys = 8	Adult	φ	0.90	0.88	0.80	0.95	Yes
nsites = 40 nyears = 5 nsurveys = 8	Juvenile	γ	0.25	0.20	0.14	0.27	Yes
nsites = 40 nyears = 5 nsurveys = 8	Adult	γ	0.01	0.69	0.02	2.33	No
nsites = 40 nyears = 5 nsurveys = 8	Juvenile	ρ	0.20	0.22	0.19	0.26	Yes
nsites = 40 nyears = 5 nsurveys = 8	Adult	ρ	0.20	0.16	0.12	0.21	Yes
nsites = 250 nyears = 5 nsurveys = 8	Juvenile	λ	1.00	10.33	9.40	11.33	Yes
nsites = 250 nyears = 5 nsurveys = 8	Adult	λ	2.00	19.80	17.92	21.76	Yes
nsites = 250 nyears = 5 nsurveys = 8	Juvenile	φ	0.50	0.56	0.46	0.67	Yes
nsites = 250 nyears = 5 nsurveys = 8	Adult	φ	0.90	0.88	0.86	0.90	Yes
nsites = 250 nyears = 5 nsurveys = 8	Juvenile	γ	0.25	0.25	0.22	0.28	Yes
nsites = 250 nyears = 5 nsurveys = 8	Adult	γ	0.01	0.09	0.00	0.32	Yes
nsites = 250 nyears = 5 nsurveys = 8	Juvenile	ρ	0.20	0.20	0.18	0.22	Yes
nsites = 250 nyears = 5 nsurveys = 8	Adult	ρ	0.20	0.20	0.18	0.22	Yes
nsites = 40 nyears = 10 nsurveys = 8	Juvenile	λ	1.00	9.35	7.79	11.11	Yes
nsites = 40 nyears = 10 nsurveys = 8	Adult	λ	2.00	20.24	16.70	24.50	Yes
nsites = 40 nyears = 10 nsurveys = 8	Juvenile	φ	0.50	0.48	0.29	0.70	Yes

nsites = 40 nyears = 10 nsurveys = 8	Adult	φ	0.90	0.89	0.84	0.93	Yes
nsites = 40 nyears = 10 nsurveys = 8	Juvenile	γ	0.25	0.26	0.21	0.32	Yes
nsites = 40 nyears = 10 nsurveys = 8	Adult	γ	0.01	0.30	0.01	0.94	Yes
nsites = 40 nyears = 10 nsurveys = 8	Juvenile	ρ	0.20	0.20	0.18	0.23	Yes
nsites = 40 nyears = 10 nsurveys = 8	Adult	ρ	0.20	0.21	0.17	0.25	Yes
nsites = 250 nyears = 10 nsurveys = 8	Juvenile	λ	1.00	10.19	9.43	10.94	Yes
nsites = 250 nyears = 10 nsurveys = 8	Adult	λ	2.00	21.08	19.32	22.91	Yes
nsites = 250 nyears = 10 nsurveys = 8	Juvenile	φ	0.50	0.52	0.44	0.61	Yes
nsites = 250 nyears = 10 nsurveys = 8	Adult	φ	0.90	0.90	0.88	0.92	Yes
nsites = 250 nyears = 10 nsurveys = 8	Juvenile	γ	0.25	0.23	0.21	0.26	Yes
nsites = 250 nyears = 10 nsurveys = 8	Adult	γ	0.01	0.06	0.00	0.22	Yes
nsites = 250 nyears = 10 nsurveys = 8	Juvenile	ρ	0.20	0.20	0.19	0.21	Yes
nsites = 250 nyears = 10 nsurveys = 8	Adult	ρ	0.20	0.19	0.17	0.20	Yes
nsites = 40 nyears = 2 nsurveys = 12	Juvenile	λ	1.00	9.95	7.36	13.86	Yes
nsites = 40 nyears = 2 nsurveys = 12	Adult	λ	2.00	16.73	13.08	21.89	Yes
nsites = 40 nyears = 2 nsurveys = 12	Juvenile	φ	0.50	0.41	0.07	0.87	Yes
nsites = 40 nyears = 2 nsurveys = 12	Adult	φ	0.90	0.78	0.52	0.97	Yes
nsites = 40 nyears = 2 nsurveys = 12	Juvenile	γ	0.25	0.31	0.20	0.46	Yes
nsites = 40 nyears = 2 nsurveys = 12	Adult	γ	0.01	1.78	0.06	5.85	No
nsites = 40 nyears = 2 nsurveys = 12	Juvenile	ρ	0.20	0.20	0.14	0.25	Yes
nsites = 40 nyears = 2 nsurveys = 12	Adult	ρ	0.20	0.25	0.19	0.31	Yes
nsites = 250 nyears = 2 nsurveys = 12	Juvenile	λ	1.00	9.71	8.68	10.92	Yes
nsites = 250 nyears = 2 nsurveys = 12	Adult	λ	2.00	20.49	18.11	23.31	Yes
nsites = 250 nyears = 2 nsurveys = 12	Juvenile	φ	0.50	0.55	0.36	0.74	Yes
nsites = 250 nyears = 2 nsurveys = 12	Adult	φ	0.90	0.85	0.76	0.93	Yes
nsites = 250 nyears = 2 nsurveys = 12	Juvenile	γ	0.25	0.24	0.20	0.28	Yes
nsites = 250 nyears = 2 nsurveys = 12	Adult	γ	0.01	0.58	0.02	1.93	No
nsites = 250 nyears = 2 nsurveys = 12	Juvenile	ρ	0.20	0.21	0.19	0.23	Yes
nsites = 250 nyears = 2 nsurveys = 12	Adult	ρ	0.20	0.20	0.17	0.22	Yes
nsites = 40 nyears = 5 nsurveys = 12	Juvenile	λ	1.00	11.52	9.49	14.02	Yes
nsites = 40 nyears = 5 nsurveys = 12	Adult	λ	2.00	19.93	16.55	23.98	Yes
nsites = 40 nyears = 5 nsurveys = 12	Juvenile	φ	0.50	0.41	0.24	0.61	Yes
nsites = 40 nyears = 5 nsurveys = 12	Adult	φ	0.90	0.88	0.81	0.93	Yes
nsites = 40 nyears = 5 nsurveys = 12	Juvenile	γ	0.25	0.29	0.23	0.37	Yes
nsites = 40 nyears = 5 nsurveys = 12	Adult	γ	0.01	0.65	0.02	2.14	No
nsites = 40 nyears = 5 nsurveys = 12	Juvenile	ρ	0.20	0.18	0.15	0.21	Yes
nsites = 40 nyears = 5 nsurveys = 12	Adult	ρ	0.20	0.22	0.18	0.25	Yes
nsites = 250 nyears = 5 nsurveys = 12	Juvenile	λ	1.00	10.13	9.35	10.92	Yes
nsites = 250 nyears = 5 nsurveys = 12	Adult	λ	2.00	20.71	19.04	22.62	Yes
nsites = 250 nyears = 5 nsurveys = 12	Juvenile	φ	0.50	0.55	0.46	0.65	Yes
nsites = 250 nyears = 5 nsurveys = 12	Adult	φ	0.90	0.89	0.87	0.91	Yes
nsites = 250 nyears = 5 nsurveys = 12	Juvenile	γ	0.25	0.24	0.21	0.26	Yes
nsites = 250 nyears = 5 nsurveys = 12	Adult	γ	0.01	0.16	0.00	0.52	Yes
nsites = 250 nyears = 5 nsurveys = 12	Juvenile	ρ	0.20	0.20	0.19	0.22	Yes
nsites = 250 nyears = 5 nsurveys = 12	Adult	ρ	0.20	0.19	0.18	0.21	Yes
nsites = 40 nyears = 10 nsurveys = 12	Juvenile	λ	1.00	10.49	8.92	12.23	Yes
nsites = 40 nyears = 10 nsurveys = 12	Adult	λ	2.00	19.80	16.76	23.38	Yes
nsites = 40 nyears = 10 nsurveys = 12	Juvenile	φ	0.50	0.37	0.24	0.51	Yes
nsites = 40 nyears = 10 nsurveys = 12	Adult	φ	0.90	0.92	0.89	0.95	Yes

nsites = 40 nyears = 10 nsurveys = 12	Juvenile	γ	0.25	0.23	0.19	0.28	Yes
nsites = 40 nyears = 10 nsurveys = 12	Adult	γ	0.01	0.36	0.02	1.00	No
nsites = 40 nyears = 10 nsurveys = 12	Juvenile	ρ	0.20	0.22	0.19	0.24	Yes
nsites = 40 nyears = 10 nsurveys = 12	Adult	ρ	0.20	0.20	0.17	0.23	Yes
nsites = 250 nyears = 10 nsurveys = 12	Juvenile	λ	1.00	10.15	9.51	10.83	Yes
nsites = 250 nyears = 10 nsurveys = 12	Adult	λ	2.00	19.68	18.44	21.00	Yes
nsites = 250 nyears = 10 nsurveys = 12	Juvenile	φ	0.50	0.48	0.42	0.55	Yes
nsites = 250 nyears = 10 nsurveys = 12	Adult	φ	0.90	0.90	0.89	0.91	Yes
nsites = 250 nyears = 10 nsurveys = 12	Juvenile	γ	0.25	0.25	0.23	0.27	Yes
nsites = 250 nyears = 10 nsurveys = 12	Adult	γ	0.01	0.04	0.00	0.15	Yes
nsites = 250 nyears = 10 nsurveys = 12	Juvenile	ρ	0.20	0.20	0.19	0.21	Yes
nsites = 250 nyears = 10 nsurveys = 12	Adult	ρ	0.20	0.20	0.19	0.21	Yes

Table S5. Mean, standard deviation (SD), and lower (2.75%) and upper (97.5%) quantiles of relative absolute deviation (RAD), expressed as the percent difference between known parameters (abundance, apparent survival, recruitment, detection probability) and those estimated using a multistate Dail-Madsen (DM) model, from 2 states (juvenile and adult) under 18 different scenarios (i.e., combinations of sites, years, and surveys) and 4 different plausible model settings, including low and high initial abundance and 25 and 50% missing data (NA). Note, that the missing data settings were only conducted using a low initial abundance.

Settings	Sites	Years	Surveys	Parameter	State	Mean	SD	Lower	Upper
Low	40	2	4	abundance	juveniles	189.65	633.98	1.63	2399.78
Low	250	2	4	abundance	juveniles	25.94	9.37	5.57	42.79
Low	40	5	4	abundance	juveniles	36.58	47.26	1.03	163.75
Low	250	5	4	abundance	juveniles	21.98	12.68	1.49	49.51
Low	40	10	4	abundance	juveniles	24.15	18.28	0.98	66.25
Low	250	10	4	abundance	juveniles	9.43	6.58	0.47	24.24
Low	40	2	8	abundance	juveniles	18.08	14.46	0.63	50.49
Low	250	2	8	abundance	juveniles	11.23	8.14	0.48	30.63
Low	40	5	8	abundance	juveniles	24.45	14.25	1.17	53.41
Low	250	5	8	abundance	juveniles	15.34	6.88	1.98	28.51
Low	40	10	8	abundance	juveniles	18.72	14.97	0.81	56.01
Low	250	10	8	abundance	juveniles	9.19	6.70	0.36	24.96
Low	40	2	12	abundance	juveniles	20.95	16.30	0.67	58.93
Low	250	2	12	abundance	juveniles	12.29	6.45	0.91	25.09
Low	40	5	12	abundance	juveniles	14.20	10.91	0.62	39.80
Low	250	5	12	abundance	juveniles	7.12	5.10	0.27	18.61
Low	40	10	12	abundance	juveniles	14.21	10.75	0.57	39.52
Low	250	10	12	abundance	juveniles	6.69	5.20	0.26	19.10
25% NA	40	2	4	abundance	juveniles	236.20	489.91	2.21	1874.81
25% NA	250	2	4	abundance	juveniles	65.00	51.03	7.16	187.71
25% NA	40	5	4	abundance	juveniles	68.96	72.07	2.35	256.33
25% NA	250	5	4	abundance	juveniles	11.81	9.04	0.46	32.02
25% NA	40	10	4	abundance	juveniles	65.02	58.23	2.90	216.77
25% NA	250	10	4	abundance	juveniles	10.87	8.81	0.36	33.30
25% NA	40	2	8	abundance	juveniles	18.95	13.10	0.90	48.58
25% NA	250	2	8	abundance	juveniles	17.76	11.48	1.07	43.12
25% NA	40	5	8	abundance	juveniles	18.24	13.64	0.73	49.69
25% NA	250	5	8	abundance	juveniles	8.80	6.91	0.28	25.81
25% NA	40	10	8	abundance	juveniles	17.32	13.00	0.75	48.03
25% NA	250	10	8	abundance	juveniles	8.99	6.72	0.32	24.75
25% NA	40	2	12	abundance	juveniles	26.37	23.68	0.72	89.24
25% NA	250	2	12	abundance	juveniles	7.58	5.44	0.31	19.95
25% NA	40	5	12	abundance	juveniles	33.21	21.71	1.83	84.44

25% NA	250	5	12	abundance	juveniles	6.06	4.54	0.24	16.96
25% NA	40	10	12	abundance	juveniles	15.69	12.36	0.55	46.13
25% NA	250	10	12	abundance	juveniles	6.58	5.09	0.25	18.84
50% NA	40	2	4	abundance	juveniles	181.41	514.46	1.52	1898.31
50% NA	250	2	4	abundance	juveniles	20.05	20.77	0.75	75.89
50% NA	40	5	4	abundance	juveniles	53.06	55.95	1.42	209.69
50% NA	250	5	4	abundance	juveniles	11.36	8.11	0.56	29.40
50% NA	40	10	4	abundance	juveniles	26.38	24.89	1.04	93.05
50% NA	250	10	4	abundance	juveniles	10.81	8.53	0.47	31.82
50% NA	40	2	8	abundance	juveniles	129.42	189.67	4.58	594.86
50% NA	250	2	8	abundance	juveniles	16.29	11.71	0.78	43.59
50% NA	40	5	8	abundance	juveniles	22.62	18.70	0.84	68.54
50% NA	250	5	8	abundance	juveniles	7.52	6.19	0.26	21.36
50% NA	40	10	8	abundance	juveniles	17.89	14.47	0.64	53.96
50% NA	250	10	8	abundance	juveniles	13.99	6.91	1.30	28.00
50% NA	40	2	12	abundance	juveniles	15.98	13.00	0.66	48.43
50% NA	250	2	12	abundance	juveniles	8.64	6.60	0.27	24.45
50% NA	40	5	12	abundance	juveniles	17.30	11.74	0.78	42.88
50% NA	250	5	12	abundance	juveniles	14.47	8.25	1.02	31.51
50% NA	40	10	12	abundance	juveniles	16.66	13.38	0.68	49.26
50% NA	250	10	12	abundance	juveniles	13.40	6.80	1.13	26.51
High	40	2	4	abundance	juveniles	32.84	36.35	0.86	134.20
High	250	2	4	abundance	juveniles	20.07	7.20	4.16	32.86
High	40	5	4	abundance	juveniles	21.04	21.66	0.64	77.95
High	250	5	4	abundance	juveniles	6.22	4.54	0.20	16.76
High	40	10	4	abundance	juveniles	11.97	10.13	0.37	38.90
High	250	10	4	abundance	juveniles	4.76	3.43	0.22	12.65
High	40	2	8	abundance	juveniles	17.30	10.36	0.84	38.03
High	250	2	8	abundance	juveniles	6.48	5.07	0.30	19.51
High	40	5	8	abundance	juveniles	8.69	6.59	0.37	24.37
High	250	5	8	abundance	juveniles	4.73	3.59	0.20	13.27
High	40	10	8	abundance	juveniles	8.91	5.99	0.46	22.23
High	250	10	8	abundance	juveniles	3.46	2.57	0.13	9.56
High	40	2	12	abundance	juveniles	12.90	10.46	0.47	38.58
High	250	2	12	abundance	juveniles	5.22	3.67	0.24	13.73
High	40	5	12	abundance	juveniles	15.88	10.64	0.78	40.20
High	250	5	12	abundance	juveniles	3.33	2.60	0.12	9.47
High	40	10	12	abundance	juveniles	7.69	6.12	0.26	22.30
High	250	10	12	abundance	juveniles	2.94	2.28	0.12	8.44
Low	40	2	4	survival	juveniles	46.31	29.33	2.04	98.27
Low	250	2	4	survival	juveniles	23.80	17.33	0.99	63.62
Low	40	5	4	survival	juveniles	66.78	21.69	14.59	97.60
Low	250	5	4	survival	juveniles	20.67	12.28	1.13	45.67
Low	40	10	4	survival	juveniles	29.02	21.55	1.25	80.57
Low	250	10	4	survival	juveniles	13.13	8.91	0.54	33.42
Low	40	2	8	survival	juveniles	59.14	25.52	5.58	97.66
Low	250	2	8	survival	juveniles	22.31	14.01	1.12	52.06
Low	40	5	8	survival	juveniles	64.71	22.27	14.40	97.78
Low	250	5	8	survival	juveniles	19.07	10.81	1.34	41.09
Low	40	10	8	survival	juveniles	16.45	11.76	0.58	42.43

Low	250	10	8	survival	juveniles	7.72	5.60	0.33	20.83
Low	40	2	12	survival	juveniles	46.10	28.02	2.20	96.22
Low	250	2	12	survival	juveniles	27.22	14.59	1.97	57.65
Low	40	5	12	survival	juveniles	20.14	15.31	0.73	58.96
Low	250	5	12	survival	juveniles	15.27	8.98	0.79	33.66
Low	40	10	12	survival	juveniles	17.93	13.89	0.65	52.01
Low	250	10	12	survival	juveniles	6.67	4.96	0.28	18.11
25% NA	40	2	4	survival	juveniles	56.25	29.19	3.31	98.57
25% NA	250	2	4	survival	juveniles	46.49	23.29	4.08	90.61
25% NA	40	5	4	survival	juveniles	58.53	21.57	10.42	94.04
25% NA	250	5	4	survival	juveniles	32.78	18.50	2.89	86.22
25% NA	40	10	4	survival	juveniles	27.78	20.54	1.11	75.91
25% NA	250	10	4	survival	juveniles	10.48	8.05	0.44	29.84
25% NA	40	2	8	survival	juveniles	42.65	27.20	1.77	94.39
25% NA	250	2	8	survival	juveniles	18.37	13.83	0.82	51.45
25% NA	40	5	8	survival	juveniles	41.11	23.36	2.49	87.74
25% NA	250	5	8	survival	juveniles	31.38	18.85	5.02	92.95
25% NA	40	10	8	survival	juveniles	27.05	16.55	1.43	61.26
25% NA	250	10	8	survival	juveniles	6.82	5.15	0.27	18.43
25% NA	40	2	12	survival	juveniles	45.15	26.33	2.08	94.13
25% NA	250	2	12	survival	juveniles	23.63	15.37	1.26	56.89
25% NA	40	5	12	survival	juveniles	21.57	15.10	0.85	54.21
25% NA	250	5	12	survival	juveniles	25.14	10.03	5.61	45.46
25% NA	40	10	12	survival	juveniles	27.05	15.04	1.81	57.39
25% NA	250	10	12	survival	juveniles	5.30	4.05	0.21	15.03
50% NA	40	2	4	survival	juveniles	60.48	28.64	4.35	98.71
50% NA	250	2	4	survival	juveniles	67.36	24.32	7.75	98.63
50% NA	40	5	4	survival	juveniles	31.99	20.78	1.40	74.28
50% NA	250	5	4	survival	juveniles	19.44	21.51	0.51	95.40
50% NA	40	10	4	survival	juveniles	21.22	16.07	1.02	58.20
50% NA	250	10	4	survival	juveniles	24.57	13.54	1.75	52.21
50% NA	40	2	8	survival	juveniles	45.07	27.82	2.01	96.09
50% NA	250	2	8	survival	juveniles	18.75	14.66	0.82	55.27
50% NA	40	5	8	survival	juveniles	35.03	19.19	2.71	72.29
50% NA	250	5	8	survival	juveniles	17.94	24.65	0.54	97.82
50% NA	40	10	8	survival	juveniles	19.09	12.80	0.70	47.34
50% NA	250	10	8	survival	juveniles	15.50	8.24	1.26	32.28
50% NA	40	2	12	survival	juveniles	32.76	23.15	1.54	86.06
50% NA	250	2	12	survival	juveniles	16.64	12.98	0.52	47.79
50% NA	40	5	12	survival	juveniles	21.38	15.80	0.79	59.44
50% NA	250	5	12	survival	juveniles	11.54	8.13	0.57	27.89
50% NA	40	10	12	survival	juveniles	13.75	9.90	0.61	37.08
50% NA	250	10	12	survival	juveniles	5.64	4.15	0.24	15.42
High	40	2	4	survival	juveniles	49.07	28.05	2.66	97.17
High	250	2	4	survival	juveniles	47.07	27.47	2.86	94.81
High	40	5	4	survival	juveniles	29.56	21.56	1.28	80.17
High	250	5	4	survival	juveniles	15.40	12.67	0.51	48.02
High	40	10	4	survival	juveniles	32.91	16.90	2.32	63.73
High	250	10	4	survival	juveniles	24.23	16.39	1.14	62.61
High	40	2	8	survival	juveniles	53.45	26.82	3.82	97.00

High	250	2	8	survival	juveniles	19.39	14.46	0.70	54.27
High	40	5	8	survival	juveniles	41.46	26.19	2.00	93.30
High	250	5	8	survival	juveniles	13.25	9.14	0.55	34.14
High	40	10	8	survival	juveniles	16.97	12.33	0.80	44.96
High	250	10	8	survival	juveniles	7.74	5.90	0.31	21.97
High	40	2	12	survival	juveniles	38.39	24.74	1.83	90.66
High	250	2	12	survival	juveniles	16.97	13.05	0.61	48.23
High	40	5	12	survival	juveniles	21.86	14.08	1.28	52.57
High	250	5	12	survival	juveniles	11.72	8.11	0.53	30.34
High	40	10	12	survival	juveniles	27.43	13.42	2.18	52.82
High	250	10	12	survival	juveniles	6.35	4.64	0.22	17.07
Low	40	2	4	recruitment	juveniles	399.25	1484.17	3.02	4173.67
Low	250	2	4	recruitment	juveniles	15.41	10.94	0.78	39.18
Low	40	5	4	recruitment	juveniles	106.79	98.02	6.67	373.45
Low	250	5	4	recruitment	juveniles	10.61	8.41	0.49	31.98
Low	40	10	4	recruitment	juveniles	17.78	12.70	0.72	44.52
Low	250	10	4	recruitment	juveniles	6.81	5.47	0.22	20.58
Low	40	2	8	recruitment	juveniles	34.74	30.60	1.36	115.45
Low	250	2	8	recruitment	juveniles	21.31	13.93	0.92	52.05
Low	40	5	8	recruitment	juveniles	16.11	13.71	0.58	51.88
Low	250	5	8	recruitment	juveniles	8.21	5.50	0.39	20.31
Low	40	10	8	recruitment	juveniles	9.23	7.13	0.31	26.53
Low	250	10	8	recruitment	juveniles	5.88	3.70	0.27	13.72
Low	40	2	12	recruitment	juveniles	27.01	16.56	1.25	61.09
Low	250	2	12	recruitment	juveniles	14.43	8.64	0.76	32.12
Low	40	5	12	recruitment	juveniles	16.36	12.73	0.71	47.83
Low	250	5	12	recruitment	juveniles	4.82	3.80	0.19	13.67
Low	40	10	12	recruitment	juveniles	8.52	6.05	0.40	22.17
Low	250	10	12	recruitment	juveniles	3.50	2.53	0.16	9.26
25% NA	40	2	4	recruitment	juveniles	149.16	334.03	3.57	1117.25
25% NA	250	2	4	recruitment	juveniles	79.45	60.40	6.92	222.50
25% NA	40	5	4	recruitment	juveniles	126.74	98.01	17.23	391.12
25% NA	250	5	4	recruitment	juveniles	19.48	18.80	0.60	76.55
25% NA	40	10	4	recruitment	juveniles	54.00	50.33	1.69	180.42
25% NA	250	10	4	recruitment	juveniles	10.99	7.90	0.51	30.20
25% NA	40	2	8	recruitment	juveniles	25.45	19.00	1.00	65.87
25% NA	250	2	8	recruitment	juveniles	12.02	8.70	0.45	31.80
25% NA	40	5	8	recruitment	juveniles	15.72	12.07	0.58	45.38
25% NA	250	5	8	recruitment	juveniles	26.58	18.87	4.22	83.85
25% NA	40	10	8	recruitment	juveniles	12.47	9.99	0.47	38.52
25% NA	250	10	8	recruitment	juveniles	4.28	3.26	0.16	12.17
25% NA	40	2	12	recruitment	juveniles	36.50	32.79	1.07	124.69
25% NA	250	2	12	recruitment	juveniles	16.34	9.35	0.90	35.31
25% NA	40	5	12	recruitment	juveniles	14.18	9.71	0.62	35.74
25% NA	250	5	12	recruitment	juveniles	11.32	5.23	1.30	21.48
25% NA	40	10	12	recruitment	juveniles	8.90	6.69	0.36	24.88
25% NA	250	10	12	recruitment	juveniles	3.17	2.41	0.13	8.87
50% NA	40	2	4	recruitment	juveniles	549.45	1162.62	9.22	4648.58
50% NA	250	2	4	recruitment	juveniles	148.22	77.23	43.10	331.60
50% NA	40	5	4	recruitment	juveniles	215.52	124.06	60.64	536.44

50% NA	250	5	4	recruitment	juveniles	72.19	21.46	31.85	115.53
50% NA	40	10	4	recruitment	juveniles	160.91	63.18	70.36	315.96
50% NA	250	10	4	recruitment	juveniles	93.33	15.71	65.81	127.31
50% NA	40	2	8	recruitment	juveniles	278.22	355.18	20.96	1218.74
50% NA	250	2	8	recruitment	juveniles	116.59	31.23	61.84	184.59
50% NA	40	5	8	recruitment	juveniles	100.95	37.73	38.62	188.31
50% NA	250	5	8	recruitment	juveniles	92.39	22.48	32.89	127.52
50% NA	40	10	8	recruitment	juveniles	121.30	31.55	66.98	190.37
50% NA	250	10	8	recruitment	juveniles	85.01	8.57	69.32	102.38
50% NA	40	2	12	recruitment	juveniles	111.55	52.61	25.78	230.12
50% NA	250	2	12	recruitment	juveniles	88.58	19.26	53.70	129.55
50% NA	40	5	12	recruitment	juveniles	98.15	30.52	40.97	162.36
50% NA	250	5	12	recruitment	juveniles	104.76	13.06	81.52	128.73
50% NA	40	10	12	recruitment	juveniles	86.99	19.51	51.77	128.61
50% NA	250	10	12	recruitment	juveniles	105.67	8.62	89.98	123.27
High	40	2	4	recruitment	juveniles	32.24	31.76	1.10	112.92
High	250	2	4	recruitment	juveniles	25.78	11.61	3.13	47.95
High	40	5	4	recruitment	juveniles	22.11	18.28	0.87	71.38
High	250	5	4	recruitment	juveniles	8.35	6.28	0.33	23.49
High	40	10	4	recruitment	juveniles	20.21	15.79	0.97	59.29
High	250	10	4	recruitment	juveniles	12.41	6.15	1.23	24.36
High	40	2	8	recruitment	juveniles	22.12	19.45	0.85	74.13
High	250	2	8	recruitment	juveniles	15.19	8.49	0.94	32.68
High	40	5	8	recruitment	juveniles	22.41	11.24	1.93	43.61
High	250	5	8	recruitment	juveniles	4.85	3.79	0.17	13.80
High	40	10	8	recruitment	juveniles	9.85	7.76	0.37	29.39
High	250	10	8	recruitment	juveniles	6.68	3.87	0.40	14.70
High	40	2	12	recruitment	juveniles	27.98	22.60	1.06	82.36
High	250	2	12	recruitment	juveniles	7.62	5.44	0.34	20.03
High	40	5	12	recruitment	juveniles	18.52	13.25	0.85	49.76
High	250	5	12	recruitment	juveniles	6.35	4.08	0.29	15.08
High	40	10	12	recruitment	juveniles	9.17	6.32	0.44	23.23
High	250	10	12	recruitment	juveniles	3.12	2.38	0.12	8.83
Low	40	2	4	detection	juveniles	37.79	26.19	1.52	97.16
Low	250	2	4	detection	juveniles	15.77	10.60	0.75	39.32
Low	40	5	4	detection	juveniles	33.61	19.58	2.05	74.91
Low	250	5	4	detection	juveniles	7.17	5.32	0.30	19.87
Low	40	10	4	detection	juveniles	21.98	13.25	1.21	49.32
Low	250	10	4	detection	juveniles	6.64	4.62	0.30	17.00
Low	40	2	8	detection	juveniles	14.07	10.51	0.65	38.96
Low	250	2	8	detection	juveniles	7.94	5.37	0.36	19.81
Low	40	5	8	detection	juveniles	9.68	7.32	0.35	27.59
Low	250	5	8	detection	juveniles	4.96	3.49	0.20	12.81
Low	40	10	8	detection	juveniles	6.54	4.83	0.24	18.20
Low	250	10	8	detection	juveniles	2.67	2.03	0.10	7.50
Low	40	2	12	detection	juveniles	16.40	10.65	0.82	39.36
Low	250	2	12	detection	juveniles	4.13	3.12	0.16	11.51
Low	40	5	12	detection	juveniles	6.91	5.24	0.27	19.40
Low	250	5	12	detection	juveniles	2.76	2.04	0.10	7.59
Low	40	10	12	detection	juveniles	5.62	4.11	0.22	15.02

Low	250	10	12	detection	juveniles	2.00	1.54	0.08	5.70
25% NA	40	2	4	detection	juveniles	44.32	28.75	1.88	95.45
25% NA	250	2	4	detection	juveniles	30.03	15.64	2.14	61.92
25% NA	40	5	4	detection	juveniles	23.51	17.39	0.77	63.97
25% NA	250	5	4	detection	juveniles	11.64	8.49	0.51	31.44
25% NA	40	10	4	detection	juveniles	37.16	16.61	4.70	69.72
25% NA	250	10	4	detection	juveniles	7.39	5.30	0.30	19.55
25% NA	40	2	8	detection	juveniles	33.01	18.75	2.09	72.65
25% NA	250	2	8	detection	juveniles	8.76	6.11	0.39	22.29
25% NA	40	5	8	detection	juveniles	11.15	8.33	0.45	30.99
25% NA	250	5	8	detection	juveniles	8.06	4.70	0.44	17.90
25% NA	40	10	8	detection	juveniles	9.38	7.01	0.40	26.08
25% NA	250	10	8	detection	juveniles	3.62	2.68	0.18	9.90
25% NA	40	2	12	detection	juveniles	31.45	13.13	4.61	56.63
25% NA	250	2	12	detection	juveniles	4.88	3.67	0.17	13.65
25% NA	40	5	12	detection	juveniles	10.15	7.17	0.44	26.83
25% NA	250	5	12	detection	juveniles	5.28	3.44	0.22	12.65
25% NA	40	10	12	detection	juveniles	7.66	5.43	0.35	19.91
25% NA	250	10	12	detection	juveniles	3.66	2.34	0.19	8.75
50% NA	40	2	4	detection	juveniles	47.83	31.91	1.70	111.89
50% NA	250	2	4	detection	juveniles	17.42	13.05	0.65	48.25
50% NA	40	5	4	detection	juveniles	24.32	16.95	1.07	62.99
50% NA	250	5	4	detection	juveniles	13.45	8.95	0.69	33.40
50% NA	40	10	4	detection	juveniles	15.37	11.75	0.76	44.29
50% NA	250	10	4	detection	juveniles	6.15	4.58	0.26	17.10
50% NA	40	2	8	detection	juveniles	38.36	22.33	2.11	84.71
50% NA	250	2	8	detection	juveniles	9.71	6.92	0.45	25.85
50% NA	40	5	8	detection	juveniles	11.95	8.92	0.51	33.58
50% NA	250	5	8	detection	juveniles	6.15	4.52	0.23	16.17
50% NA	40	10	8	detection	juveniles	10.31	7.34	0.50	27.21
50% NA	250	10	8	detection	juveniles	11.83	4.04	3.76	19.94
50% NA	40	2	12	detection	juveniles	19.41	12.77	0.87	47.49
50% NA	250	2	12	detection	juveniles	6.94	4.85	0.32	17.36
50% NA	40	5	12	detection	juveniles	13.36	8.87	0.59	32.85
50% NA	250	5	12	detection	juveniles	4.88	3.30	0.18	12.49
50% NA	40	10	12	detection	juveniles	7.88	5.55	0.26	20.81
50% NA	250	10	12	detection	juveniles	3.17	2.27	0.14	8.35
High	40	2	4	detection	juveniles	20.17	14.51	0.99	55.27
High	250	2	4	detection	juveniles	22.50	10.33	2.47	43.03
High	40	5	4	detection	juveniles	15.31	11.71	0.53	43.53
High	250	5	4	detection	juveniles	5.85	4.25	0.23	15.96
High	40	10	4	detection	juveniles	9.07	6.84	0.40	25.35
High	250	10	4	detection	juveniles	3.93	2.96	0.17	10.95
High	40	2	8	detection	juveniles	20.72	14.29	0.82	53.40
High	250	2	8	detection	juveniles	5.42	4.02	0.25	15.02
High	40	5	8	detection	juveniles	12.97	8.76	0.52	32.42
High	250	5	8	detection	juveniles	3.43	2.61	0.14	9.87
High	40	10	8	detection	juveniles	5.88	4.30	0.22	16.50
High	250	10	8	detection	juveniles	2.38	1.79	0.09	6.52
High	40	2	12	detection	juveniles	11.89	8.82	0.36	33.54

High	250	2	12	detection	juveniles	5.72	4.07	0.24	15.02
High	40	5	12	detection	juveniles	11.45	6.87	0.71	26.20
High	250	5	12	detection	juveniles	2.86	2.18	0.11	8.17
High	40	10	12	detection	juveniles	8.75	5.37	0.57	20.50
High	250	10	12	detection	juveniles	2.44	1.79	0.09	6.71
Low	40	2	4	abundance	adults	22.87	15.53	1.15	53.92
Low	250	2	4	abundance	adults	9.76	6.48	0.46	23.65
Low	40	5	4	abundance	adults	13.62	10.12	0.48	37.30
Low	250	5	4	abundance	adults	6.18	4.49	0.25	16.76
Low	40	10	4	abundance	adults	52.97	27.05	6.59	114.58
Low	250	10	4	abundance	adults	5.08	3.85	0.19	14.07
Low	40	2	8	abundance	adults	31.67	12.38	5.88	53.77
Low	250	2	8	abundance	adults	5.93	4.44	0.23	16.24
Low	40	5	8	abundance	adults	11.79	9.44	0.49	35.00
Low	250	5	8	abundance	adults	5.47	4.25	0.23	15.57
Low	40	10	8	abundance	adults	19.51	13.71	0.70	51.29
Low	250	10	8	abundance	adults	4.83	3.75	0.20	14.11
Low	40	2	12	abundance	adults	17.68	14.93	0.59	56.02
Low	250	2	12	abundance	adults	5.16	3.88	0.20	14.62
Low	40	5	12	abundance	adults	15.15	9.77	0.71	36.06
Low	250	5	12	abundance	adults	5.13	3.98	0.20	14.55
Low	40	10	12	abundance	adults	10.66	8.19	0.43	29.92
Low	250	10	12	abundance	adults	4.84	3.66	0.19	13.63
25% NA	40	2	4	abundance	adults	144.59	185.42	3.71	594.76
25% NA	250	2	4	abundance	adults	12.64	7.89	0.65	28.88
25% NA	40	5	4	abundance	adults	19.16	11.48	1.01	42.41
25% NA	250	5	4	abundance	adults	33.02	93.19	0.59	418.12
25% NA	40	10	4	abundance	adults	16.57	13.63	0.60	49.21
25% NA	250	10	4	abundance	adults	10.46	5.70	0.70	21.91
25% NA	40	2	8	abundance	adults	20.04	17.79	0.78	65.78
25% NA	250	2	8	abundance	adults	11.84	8.05	0.66	30.49
25% NA	40	5	8	abundance	adults	17.40	16.66	0.64	54.10
25% NA	250	5	8	abundance	adults	57.66	168.40	0.25	705.66
25% NA	40	10	8	abundance	adults	12.08	8.77	0.55	32.49
25% NA	250	10	8	abundance	adults	8.55	5.42	0.41	20.50
25% NA	40	2	12	abundance	adults	13.32	10.50	0.46	37.36
25% NA	250	2	12	abundance	adults	9.82	6.94	0.43	25.58
25% NA	40	5	12	abundance	adults	22.83	15.82	0.97	58.69
25% NA	250	5	12	abundance	adults	4.71	3.57	0.17	13.41
25% NA	40	10	12	abundance	adults	12.48	9.79	0.52	37.42
25% NA	250	10	12	abundance	adults	5.02	3.66	0.18	13.62
50% NA	40	2	4	abundance	adults	31.66	28.47	1.42	74.64
50% NA	250	2	4	abundance	adults	11.55	8.78	0.49	32.31
50% NA	40	5	4	abundance	adults	17.74	12.28	0.74	44.07
50% NA	250	5	4	abundance	adults	34.95	109.18	0.37	438.41
50% NA	40	10	4	abundance	adults	19.64	12.19	0.96	44.66
50% NA	250	10	4	abundance	adults	5.97	4.50	0.23	16.71
50% NA	40	2	8	abundance	adults	19.17	14.46	0.78	50.60
50% NA	250	2	8	abundance	adults	14.09	6.59	1.49	26.80
50% NA	40	5	8	abundance	adults	15.98	12.90	0.65	48.62

50% NA	250	5	8	abundance	adults	34.99	91.36	0.33	379.44
50% NA	40	10	8	abundance	adults	13.07	10.36	0.50	39.14
50% NA	250	10	8	abundance	adults	5.00	3.65	0.24	13.70
50% NA	40	2	12	abundance	adults	12.66	9.67	0.45	35.82
50% NA	250	2	12	abundance	adults	12.60	7.42	0.88	28.16
50% NA	40	5	12	abundance	adults	13.43	10.85	0.55	40.72
50% NA	250	5	12	abundance	adults	6.33	15.96	0.19	16.40
50% NA	40	10	12	abundance	adults	25.50	15.20	1.51	58.01
50% NA	250	10	12	abundance	adults	14.49	6.07	2.71	26.62
High	40	2	4	abundance	adults	26.40	24.24	0.94	97.12
High	250	2	4	abundance	adults	13.32	11.65	0.52	46.91
High	40	5	4	abundance	adults	19.37	17.92	0.57	69.13
High	250	5	4	abundance	adults	7.69	6.83	0.25	26.81
High	40	10	4	abundance	adults	10.50	8.88	0.32	30.21
High	250	10	4	abundance	adults	16.69	7.44	4.08	33.36
High	40	2	8	abundance	adults	26.84	10.70	3.07	44.81
High	250	2	8	abundance	adults	11.19	8.60	0.48	32.56
High	40	5	8	abundance	adults	27.04	17.86	1.13	66.74
High	250	5	8	abundance	adults	3.84	2.92	0.16	11.05
High	40	10	8	abundance	adults	7.78	6.12	0.29	22.66
High	250	10	8	abundance	adults	5.90	3.84	0.29	14.55
High	40	2	12	abundance	adults	17.63	9.15	1.18	34.64
High	250	2	12	abundance	adults	5.67	4.38	0.26	16.55
High	40	5	12	abundance	adults	7.56	5.89	0.29	21.19
High	250	5	12	abundance	adults	4.63	3.55	0.20	13.09
High	40	10	12	abundance	adults	6.75	5.13	0.26	19.09
High	250	10	12	abundance	adults	2.95	2.13	0.10	7.82
Low	40	2	4	survival	adults	26.27	21.25	0.95	76.94
Low	250	2	4	survival	adults	6.32	4.86	0.24	18.97
Low	40	5	4	survival	adults	7.31	2.94	0.80	10.97
Low	250	5	4	survival	adults	2.88	1.90	0.13	7.01
Low	40	10	4	survival	adults	5.31	4.18	0.19	15.32
Low	250	10	4	survival	adults	1.38	1.07	0.07	4.03
Low	40	2	8	survival	adults	7.37	6.27	0.42	24.30
Low	250	2	8	survival	adults	4.27	3.31	0.18	12.51
Low	40	5	8	survival	adults	3.89	2.54	0.18	9.35
Low	250	5	8	survival	adults	1.52	1.16	0.07	4.45
Low	40	10	8	survival	adults	2.07	1.58	0.08	5.91
Low	250	10	8	survival	adults	0.85	0.65	0.03	2.43
Low	40	2	12	survival	adults	14.92	13.38	0.44	48.60
Low	250	2	12	survival	adults	5.90	3.72	0.30	13.98
Low	40	5	12	survival	adults	6.70	4.17	0.38	15.68
Low	250	5	12	survival	adults	1.91	1.33	0.08	4.85
Low	40	10	12	survival	adults	2.01	1.48	0.08	5.29
Low	250	10	12	survival	adults	0.90	0.67	0.04	2.46
25% NA	40	2	4	survival	adults	28.47	23.77	0.87	84.39
25% NA	250	2	4	survival	adults	21.06	11.65	1.68	46.17
25% NA	40	5	4	survival	adults	5.20	4.49	0.22	17.13
25% NA	250	5	4	survival	adults	4.70	2.70	0.34	10.90
25% NA	40	10	4	survival	adults	11.52	6.49	1.22	26.58

25% NA	250	10	4	survival	adults	2.58	1.47	0.19	5.76
25% NA	40	2	8	survival	adults	9.47	8.48	0.39	32.35
25% NA	250	2	8	survival	adults	5.81	4.42	0.22	16.66
25% NA	40	5	8	survival	adults	4.28	3.46	0.17	13.24
25% NA	250	5	8	survival	adults	2.96	2.75	0.11	10.89
25% NA	40	10	8	survival	adults	4.01	2.26	0.24	8.38
25% NA	250	10	8	survival	adults	1.01	0.78	0.04	2.84
25% NA	40	2	12	survival	adults	13.18	11.08	0.43	40.48
25% NA	250	2	12	survival	adults	4.07	2.99	0.18	10.61
25% NA	40	5	12	survival	adults	4.75	3.50	0.18	13.21
25% NA	250	5	12	survival	adults	2.19	1.44	0.08	5.32
25% NA	40	10	12	survival	adults	4.29	2.56	0.26	9.78
25% NA	250	10	12	survival	adults	0.91	0.64	0.03	2.38
50% NA	40	2	4	survival	adults	52.05	25.97	4.15	95.93
50% NA	250	2	4	survival	adults	9.22	8.06	0.31	31.01
50% NA	40	5	4	survival	adults	15.12	10.76	0.65	40.04
50% NA	250	5	4	survival	adults	6.43	2.95	0.59	10.99
50% NA	40	10	4	survival	adults	4.01	3.29	0.18	12.98
50% NA	250	10	4	survival	adults	5.49	2.82	0.58	11.37
50% NA	40	2	8	survival	adults	21.38	20.10	0.54	74.57
50% NA	250	2	8	survival	adults	6.30	5.04	0.25	19.18
50% NA	40	5	8	survival	adults	5.62	4.31	0.26	17.06
50% NA	250	5	8	survival	adults	2.88	2.96	0.07	11.02
50% NA	40	10	8	survival	adults	4.87	2.85	0.26	10.17
50% NA	250	10	8	survival	adults	2.61	1.54	0.15	5.95
50% NA	40	2	12	survival	adults	9.99	8.39	0.38	31.59
50% NA	250	2	12	survival	adults	4.76	3.68	0.21	14.03
50% NA	40	5	12	survival	adults	5.37	4.31	0.22	16.27
50% NA	250	5	12	survival	adults	1.79	1.53	0.07	5.60
50% NA	40	10	12	survival	adults	2.93	2.36	0.11	8.93
50% NA	250	10	12	survival	adults	1.40	1.06	0.05	3.92
High	40	2	4	survival	adults	29.65	22.84	1.12	82.52
High	250	2	4	survival	adults	9.08	6.74	0.45	24.82
High	40	5	4	survival	adults	10.59	7.06	0.56	27.55
High	250	5	4	survival	adults	2.19	1.68	0.08	6.24
High	40	10	4	survival	adults	2.68	1.78	0.13	6.52
High	250	10	4	survival	adults	1.67	1.27	0.07	4.58
High	40	2	8	survival	adults	14.05	12.30	0.48	45.60
High	250	2	8	survival	adults	6.87	5.27	0.25	19.48
High	40	5	8	survival	adults	3.52	2.85	0.13	10.63
High	250	5	8	survival	adults	2.26	1.30	0.14	4.98
High	40	10	8	survival	adults	2.24	1.76	0.08	6.68
High	250	10	8	survival	adults	0.74	0.55	0.03	2.05
High	40	2	12	survival	adults	14.56	11.77	0.48	42.51
High	250	2	12	survival	adults	6.17	4.20	0.31	15.52
High	40	5	12	survival	adults	3.34	2.71	0.14	10.13
High	250	5	12	survival	adults	1.45	1.03	0.04	3.81
High	40	10	12	survival	adults	2.41	1.43	0.13	5.26
High	250	10	12	survival	adults	0.67	0.49	0.03	1.79
Low	40	2	4	recruitment	adults	6479.82	4933.90	237.50	18333.23

Low	250	2	4	recruitment	adults	917.31	836.54	19.55	3038.20
Low	40	5	4	recruitment	adults	676.68	657.84	12.15	2370.19
Low	250	5	4	recruitment	adults	233.50	210.44	7.25	746.79
Low	40	10	4	recruitment	adults	1491.28	1044.39	45.48	3932.32
Low	250	10	4	recruitment	adults	63.74	47.98	3.41	188.32
Low	40	2	8	recruitment	adults	3073.57	1977.09	147.12	7671.44
Low	250	2	8	recruitment	adults	607.90	607.13	12.70	2163.78
Low	40	5	8	recruitment	adults	1586.99	783.39	295.94	3311.63
Low	250	5	8	recruitment	adults	95.32	99.38	3.15	385.65
Low	40	10	8	recruitment	adults	213.15	224.31	4.89	822.89
Low	250	10	8	recruitment	adults	67.20	57.93	2.76	219.23
Low	40	2	12	recruitment	adults	4995.95	4087.28	168.99	15777.28
Low	250	2	12	recruitment	adults	592.50	538.65	14.14	1888.41
Low	40	5	12	recruitment	adults	622.41	487.55	17.53	1781.88
Low	250	5	12	recruitment	adults	278.77	187.07	12.85	696.52
Low	40	10	12	recruitment	adults	226.36	205.19	5.83	748.32
Low	250	10	12	recruitment	adults	66.45	55.45	2.59	202.38
25% NA	40	2	4	recruitment	adults	15967.38	21148.77	272.22	71480.15
25% NA	250	2	4	recruitment	adults	3583.55	2399.91	112.50	9000.99
25% NA	40	5	4	recruitment	adults	903.42	754.81	20.34	2776.25
25% NA	250	5	4	recruitment	adults	442.94	332.23	15.26	1217.31
25% NA	40	10	4	recruitment	adults	295.44	322.96	5.91	1155.12
25% NA	250	10	4	recruitment	adults	128.22	102.02	4.80	373.68
25% NA	40	2	8	recruitment	adults	1498.67	1671.51	22.34	6072.66
25% NA	250	2	8	recruitment	adults	964.48	880.27	16.77	3214.30
25% NA	40	5	8	recruitment	adults	2008.59	1147.33	194.13	4593.40
25% NA	250	5	8	recruitment	adults	161.78	167.28	4.51	609.33
25% NA	40	10	8	recruitment	adults	322.18	285.30	8.08	1033.62
25% NA	250	10	8	recruitment	adults	54.42	48.67	2.07	185.70
25% NA	40	2	12	recruitment	adults	3390.00	2811.35	69.43	10435.16
25% NA	250	2	12	recruitment	adults	1023.69	856.92	18.69	3157.24
25% NA	40	5	12	recruitment	adults	400.97	439.19	8.66	1561.35
25% NA	250	5	12	recruitment	adults	132.62	132.65	3.81	483.59
25% NA	40	10	12	recruitment	adults	502.28	340.89	19.12	1281.26
25% NA	250	10	12	recruitment	adults	74.10	61.67	2.81	225.07
50% NA	40	2	4	recruitment	adults	10154.61	6931.11	1117.54	26339.95
50% NA	250	2	4	recruitment	adults	4563.88	2460.90	553.07	9951.91
50% NA	40	5	4	recruitment	adults	1282.10	1105.32	29.89	4156.31
50% NA	250	5	4	recruitment	adults	189.90	285.21	5.11	813.23
50% NA	40	10	4	recruitment	adults	540.98	484.79	10.51	1761.83
50% NA	250	10	4	recruitment	adults	152.16	119.52	5.55	449.26
50% NA	40	2	8	recruitment	adults	10643.18	7112.79	651.73	27010.40
50% NA	250	2	8	recruitment	adults	871.63	806.83	19.16	2865.77
50% NA	40	5	8	recruitment	adults	718.20	715.96	13.16	2625.05
50% NA	250	5	8	recruitment	adults	123.24	141.67	3.57	509.68
50% NA	40	10	8	recruitment	adults	507.01	372.03	16.63	1379.40
50% NA	250	10	8	recruitment	adults	122.40	100.16	4.19	363.06
50% NA	40	2	12	recruitment	adults	1922.08	1920.15	23.91	7098.17
50% NA	250	2	12	recruitment	adults	631.71	635.57	14.14	2267.20
50% NA	40	5	12	recruitment	adults	884.86	781.87	21.49	2869.54

50% NA	250	5	12	recruitment	adults	108.45	112.96	3.42	417.89
50% NA	40	10	12	recruitment	adults	284.71	284.07	6.38	1011.35
50% NA	250	10	12	recruitment	adults	163.23	122.13	5.62	442.08
High	40	2	4	recruitment	adults	71466.31	61524.45	2905.83	242157.61
High	250	2	4	recruitment	adults	10080.62	10532.65	150.71	38481.23
High	40	5	4	recruitment	adults	18739.97	15986.44	462.34	59462.22
High	250	5	4	recruitment	adults	1521.29	1477.55	21.46	5673.87
High	40	10	4	recruitment	adults	3057.25	2709.87	63.51	9857.03
High	250	10	4	recruitment	adults	503.87	577.78	8.32	2078.26
High	40	2	8	recruitment	adults	27066.58	20845.10	1390.65	79142.43
High	250	2	8	recruitment	adults	12447.86	10118.38	524.20	37644.26
High	40	5	8	recruitment	adults	6820.72	6543.18	108.52	23177.15
High	250	5	8	recruitment	adults	764.95	843.01	12.49	3115.11
High	40	10	8	recruitment	adults	2930.08	2511.27	61.53	9305.18
High	250	10	8	recruitment	adults	507.57	543.91	10.11	2056.52
High	40	2	12	recruitment	adults	17682.87	15715.75	505.58	58445.93
High	250	2	12	recruitment	adults	5668.12	5352.23	82.52	19237.63
High	40	5	12	recruitment	adults	6363.27	5626.75	146.76	21259.12
High	250	5	12	recruitment	adults	1478.31	1403.93	29.09	5088.19
High	40	10	12	recruitment	adults	3523.11	2654.32	80.03	9905.79
High	250	10	12	recruitment	adults	331.94	383.46	6.15	1395.64
Low	40	2	4	detection	adults	17.94	13.70	0.79	52.04
Low	250	2	4	detection	adults	7.23	5.51	0.23	20.41
Low	40	5	4	detection	adults	12.57	8.96	0.58	32.93
Low	250	5	4	detection	adults	5.66	4.13	0.23	15.35
Low	40	10	4	detection	adults	28.03	9.42	9.30	46.16
Low	250	10	4	detection	adults	3.64	2.67	0.13	9.92
Low	40	2	8	detection	adults	25.79	14.20	2.08	54.48
Low	250	2	8	detection	adults	6.23	4.63	0.28	16.98
Low	40	5	8	detection	adults	7.24	5.34	0.32	20.22
Low	250	5	8	detection	adults	3.00	2.26	0.12	8.20
Low	40	10	8	detection	adults	6.81	5.11	0.27	19.19
Low	250	10	8	detection	adults	4.05	2.37	0.28	9.20
Low	40	2	12	detection	adults	21.32	10.89	1.73	43.14
Low	250	2	12	detection	adults	3.87	2.85	0.15	10.62
Low	40	5	12	detection	adults	5.91	4.53	0.19	16.97
Low	250	5	12	detection	adults	2.41	1.83	0.08	6.76
Low	40	10	12	detection	adults	9.14	4.93	0.68	19.51
Low	250	10	12	detection	adults	2.69	1.69	0.10	6.36
25% NA	40	2	4	detection	adults	48.51	22.09	4.41	86.02
25% NA	250	2	4	detection	adults	16.37	10.48	0.74	39.84
25% NA	40	5	4	detection	adults	31.62	15.64	2.97	62.85
25% NA	250	5	4	detection	adults	13.59	17.48	0.49	80.14
25% NA	40	10	4	detection	adults	13.39	8.99	0.56	32.74
25% NA	250	10	4	detection	adults	7.98	4.36	0.61	16.74
25% NA	40	2	8	detection	adults	14.22	10.31	0.64	38.00
25% NA	250	2	8	detection	adults	6.10	4.61	0.22	17.02
25% NA	40	5	8	detection	adults	8.97	7.25	0.33	24.90
25% NA	250	5	8	detection	adults	11.19	23.05	0.16	86.59
25% NA	40	10	8	detection	adults	9.53	6.46	0.40	23.90

25% NA	250	10	8	detection	adults	2.63	1.94	0.11	7.19
25% NA	40	2	12	detection	adults	12.27	8.88	0.48	32.66
25% NA	250	2	12	detection	adults	10.02	5.07	0.87	20.19
25% NA	40	5	12	detection	adults	11.01	7.03	0.46	26.43
25% NA	250	5	12	detection	adults	4.81	2.86	0.30	10.69
25% NA	40	10	12	detection	adults	5.17	3.82	0.20	14.31
25% NA	250	10	12	detection	adults	3.64	1.96	0.25	7.57
50% NA	40	2	4	detection	adults	27.96	21.06	1.02	77.76
50% NA	250	2	4	detection	adults	10.76	8.06	0.44	29.08
50% NA	40	5	4	detection	adults	19.81	14.16	0.99	51.71
50% NA	250	5	4	detection	adults	10.70	17.92	0.19	77.76
50% NA	40	10	4	detection	adults	11.60	8.67	0.47	32.09
50% NA	250	10	4	detection	adults	4.88	3.44	0.23	12.96
50% NA	40	2	8	detection	adults	16.77	12.07	0.68	44.97
50% NA	250	2	8	detection	adults	8.05	5.70	0.30	21.21
50% NA	40	5	8	detection	adults	10.05	7.56	0.43	28.31
50% NA	250	5	8	detection	adults	12.63	19.74	0.32	77.09
50% NA	40	10	8	detection	adults	11.60	7.36	0.59	27.84
50% NA	250	10	8	detection	adults	3.02	2.29	0.12	8.58
50% NA	40	2	12	detection	adults	25.13	13.48	1.85	52.37
50% NA	250	2	12	detection	adults	4.68	3.60	0.20	13.31
50% NA	40	5	12	detection	adults	8.98	6.74	0.38	25.11
50% NA	250	5	12	detection	adults	3.26	5.01	0.14	8.34
50% NA	40	10	12	detection	adults	8.59	5.42	0.45	20.10
50% NA	250	10	12	detection	adults	2.64	1.94	0.10	7.10
High	40	2	4	detection	adults	23.88	17.57	0.97	64.46
High	250	2	4	detection	adults	11.55	8.33	0.41	32.12
High	40	5	4	detection	adults	15.31	11.07	0.60	41.10
High	250	5	4	detection	adults	6.32	5.17	0.24	19.02
High	40	10	4	detection	adults	13.92	10.22	0.61	38.02
High	250	10	4	detection	adults	12.42	5.40	2.71	23.88
High	40	2	8	detection	adults	34.17	18.17	2.35	70.89
High	250	2	8	detection	adults	7.36	5.63	0.30	20.99
High	40	5	8	detection	adults	20.84	10.38	1.91	40.36
High	250	5	8	detection	adults	3.77	2.94	0.13	10.90
High	40	10	8	detection	adults	8.40	6.42	0.34	23.17
High	250	10	8	detection	adults	6.45	3.47	0.43	13.40
High	40	2	12	detection	adults	27.42	14.77	2.14	57.18
High	250	2	12	detection	adults	5.15	3.79	0.19	14.05
High	40	5	12	detection	adults	9.88	7.21	0.45	26.62
High	250	5	12	detection	adults	4.64	3.20	0.21	11.87
High	40	10	12	detection	adults	6.05	4.56	0.23	16.79
High	250	10	12	detection	adults	2.48	1.87	0.09	7.05

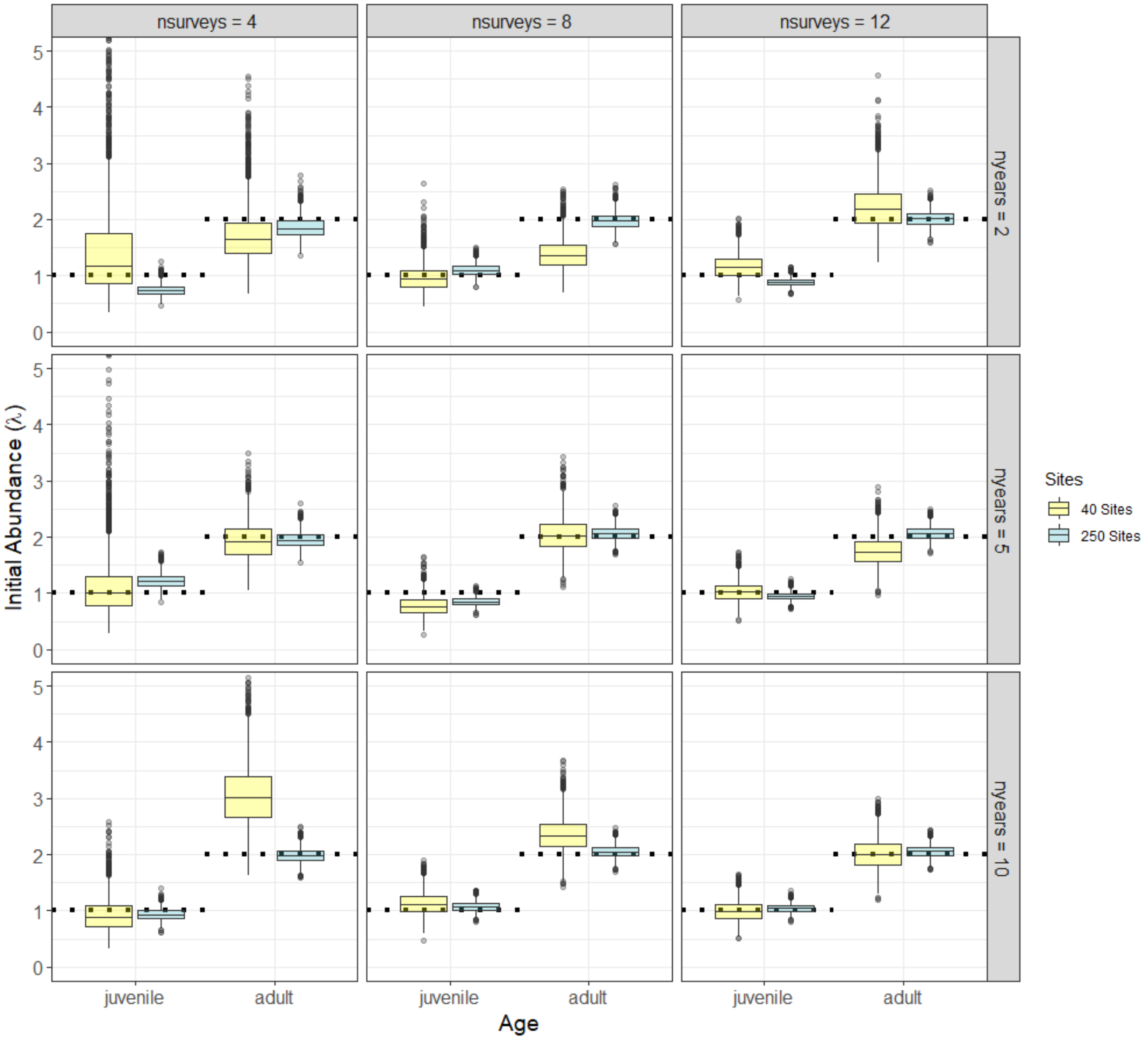

Figure S1. Boxplots of Monte Carlo Markov chain (MCMC) draws (n = 3,000) of simulated initial abundance for juveniles and adults compared to known abundances for each age class (horizontal dashed lines) under 18 different sampling scenarios assuming a low initial abundance with no missing data and equal detection probability for both age classes (ρ = 0.2). Note that the y-axis is limited between 0 and 5 to improve visual comparison across scenarios but the outliers of some boxplots extend considerably higher for some scenarios (e.g., the highest value for initial abundance was 50 under a scenario with 40 sites, 2 years, and 4 surveys).

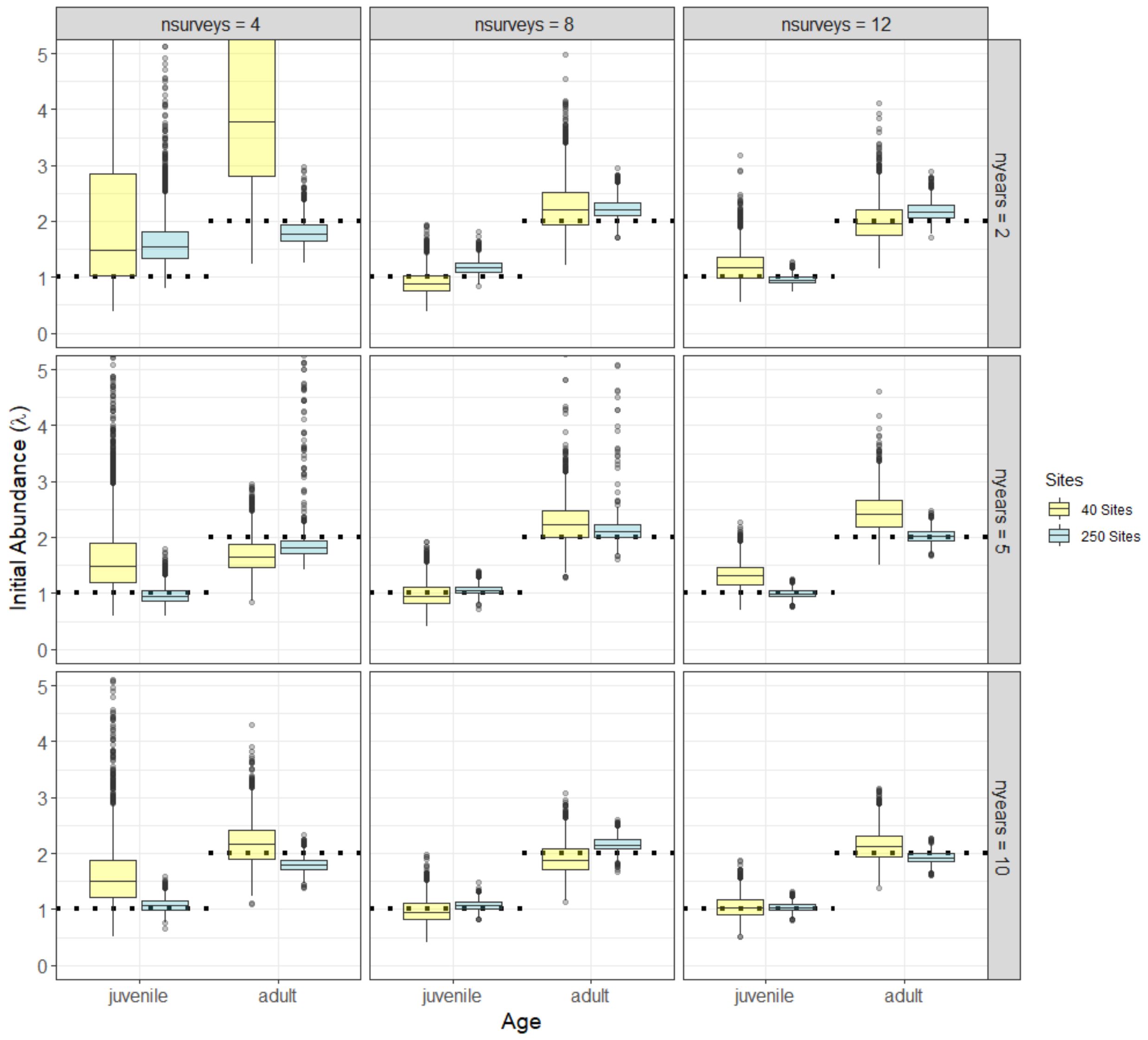

Figure S2. Boxplots of Monte Carlo Markov chain (MCMC) draws (n = 3,000) of simulated initial abundance for juveniles and adults compared to known abundances for each age class (horizontal dashed lines) under 18 different sampling scenarios assuming a low initial abundance with 25% missing data and equal detection probability for both age classes (ρ = 0.2). Note that the y-axis is limited between 0 and 5 to improve visual comparison across scenarios but the outliers of some boxplots extend considerably higher for some scenarios (e.g., the highest value for initial abundance was 44 under a scenario with 40 sites, 2 years, and 4 surveys).

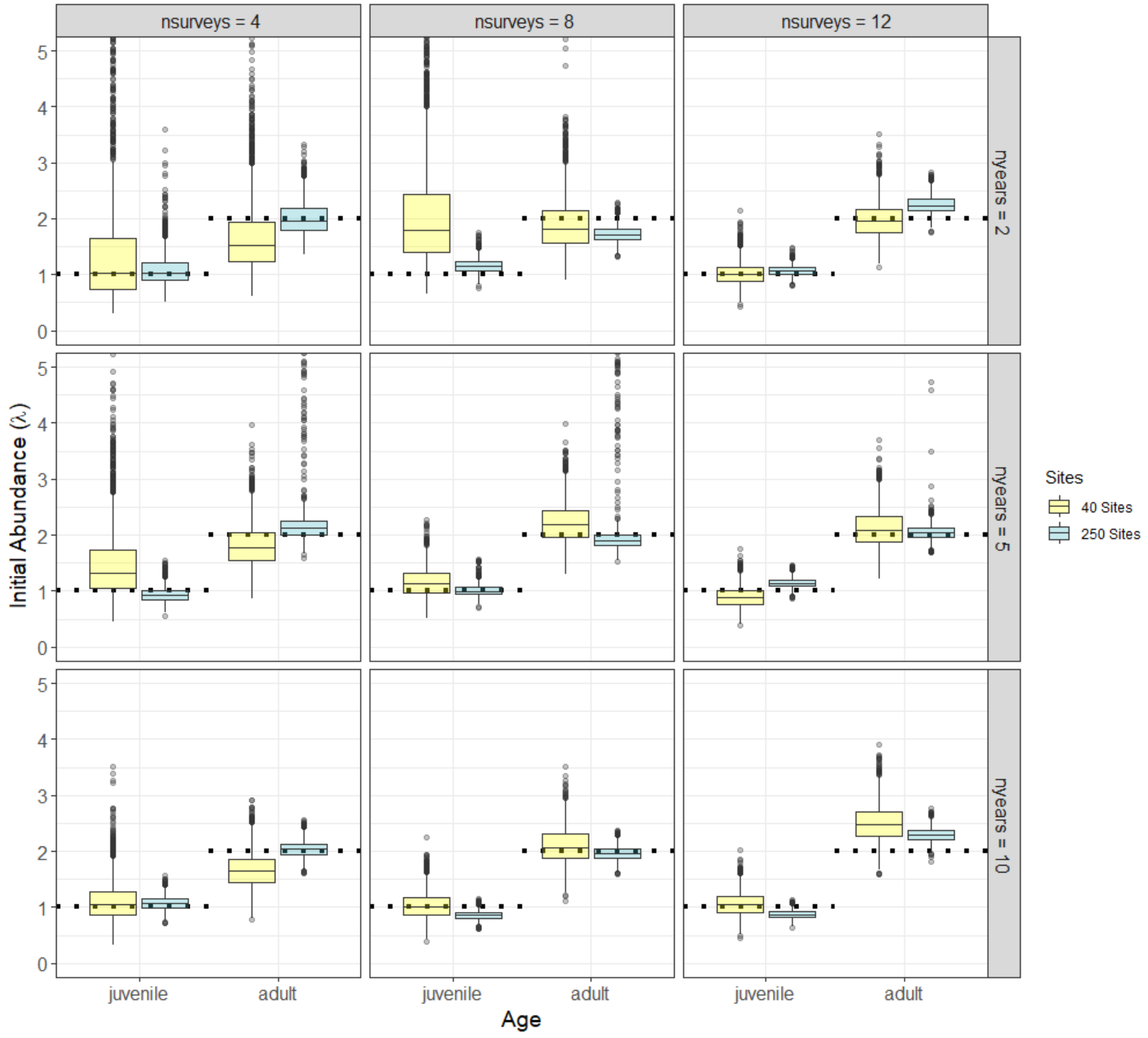

Figure S3. Boxplots of Monte Carlo Markov chain (MCMC) draws (n = 3,000) of simulated initial abundance for juveniles and adults compared to known abundances for each age class (horizontal dashed lines) under 18 different sampling scenarios assuming a low initial abundance with 50% missing data and equal detection probability for both age classes (ρ = 0.2). Note that the y-axis is limited between 0 and 5 to improve visual comparison across scenarios but the outliers of some boxplots extend considerably higher for some scenarios (e.g., the highest value for initial abundance was 49 under a scenario with 40 sites, 2 years, and 4 surveys).

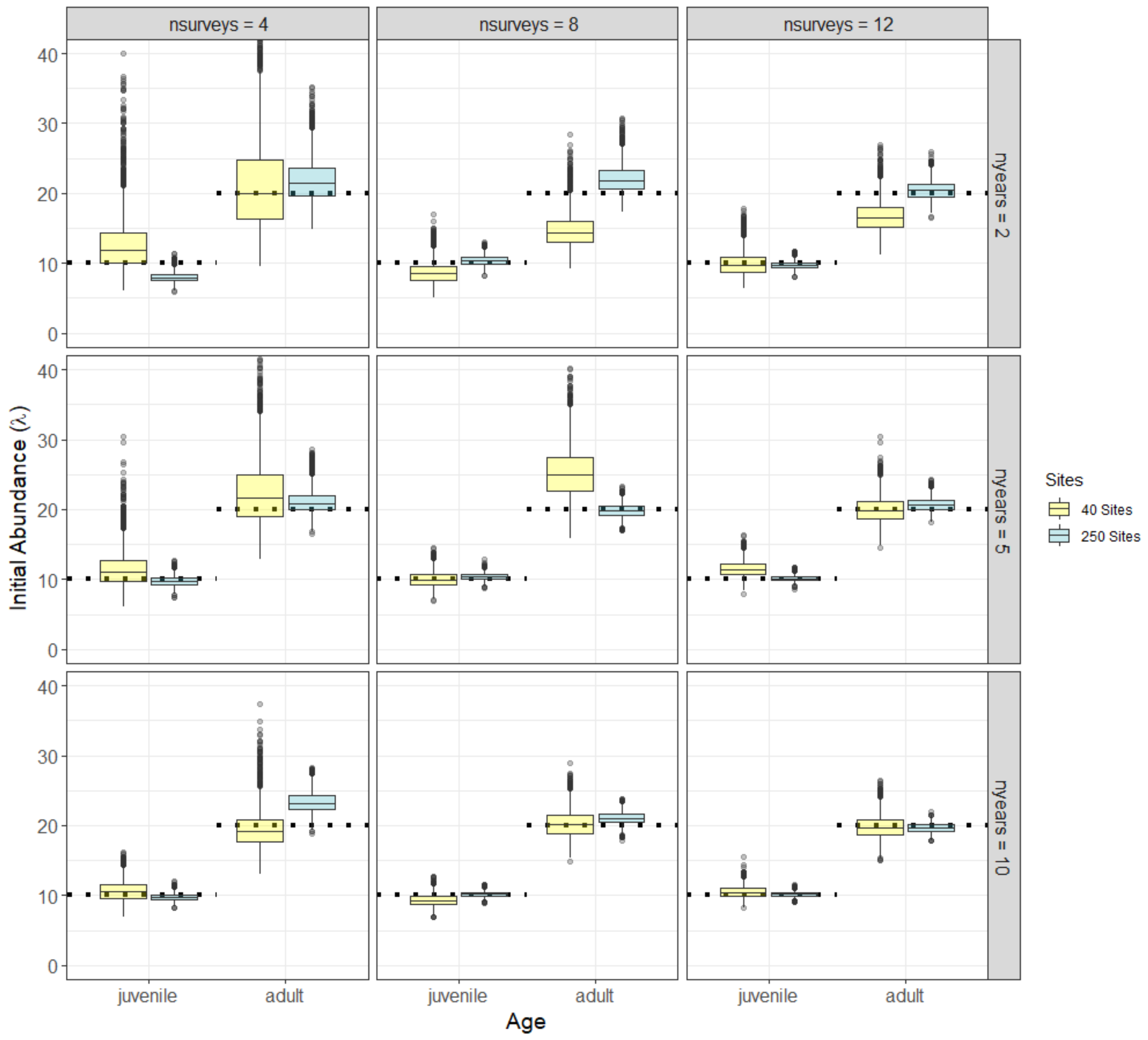

Figure S4. Boxplots of Monte Carlo Markov chain (MCMC) draws (n = 3,000) of simulated initial abundance for juveniles and adults compared to known abundances for each age class (horizontal dashed lines) under 18 different sampling scenarios assuming a high initial abundance with no missing data and equal detection probability for both age classes (ρ = 0.2). Note that the y-axis is limited between 0 and 40 to improve visual comparison across scenarios but the outliers of some boxplots extend considerably higher for some scenarios (e.g., the highest value for initial abundance was 50 under a scenario with 40 sites, 2 years, and 4 surveys).

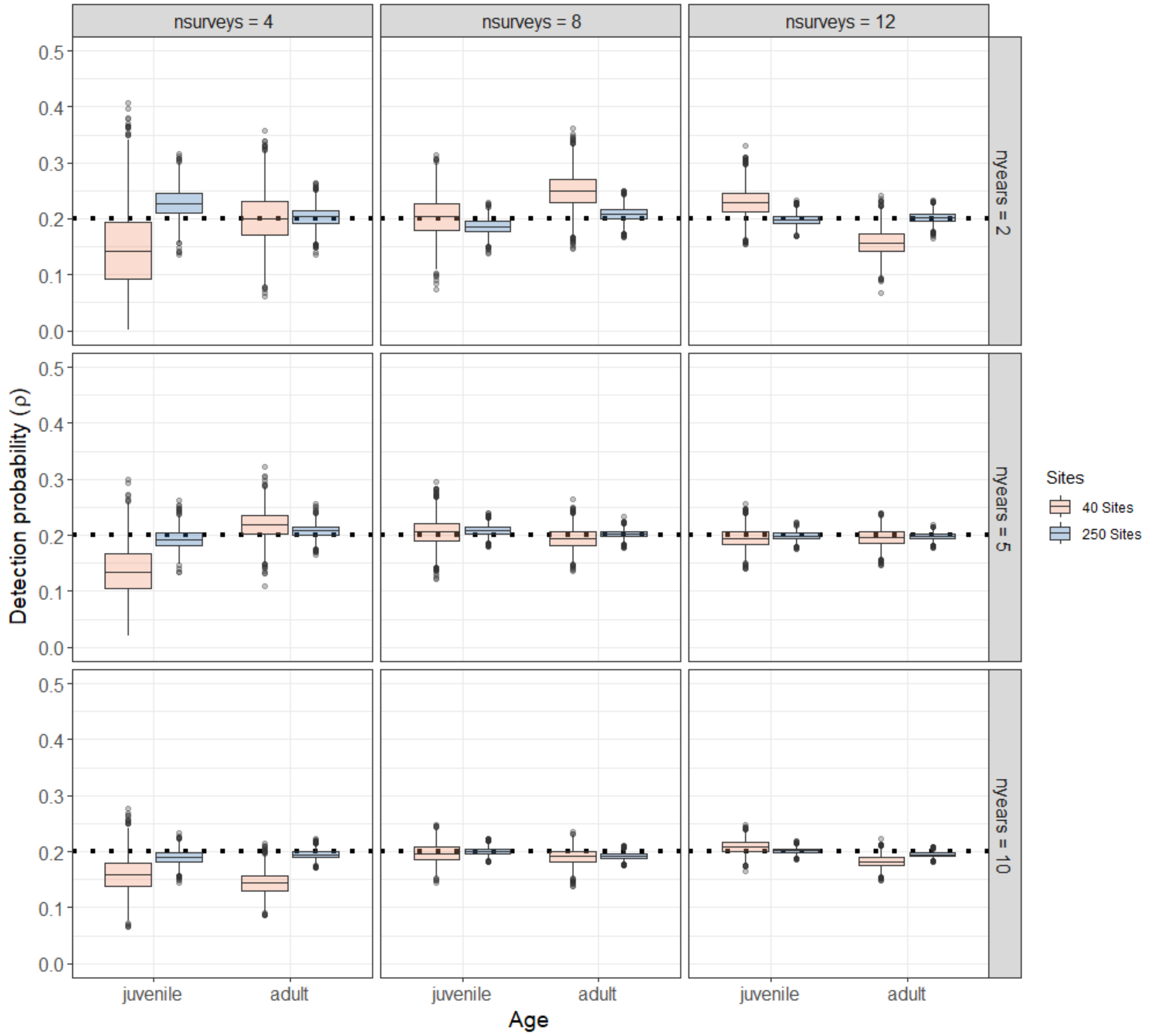

Figure S5. Boxplots of Monte Carlo Markov chain (MCMC) draws (n = 3,000) of simulated detection probability for juveniles and adults compared to known and equal detection probability for both age classes (ρ = 0.2; horizontal dashed lines) under 18 different sampling scenarios assuming a low initial abundance with no missing data.

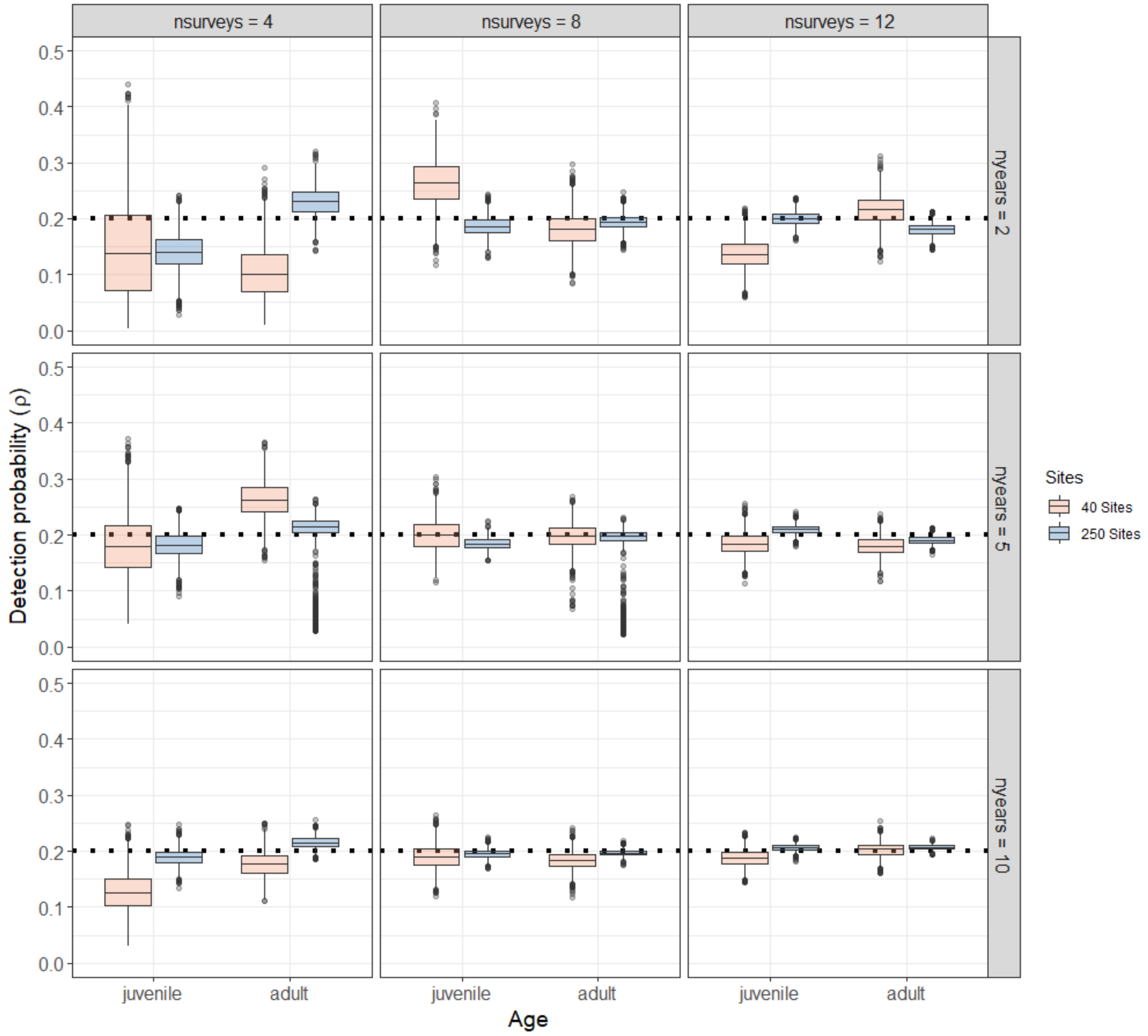

Figure S6. Boxplots of Monte Carlo Markov chain (MCMC) draws (n = 3,000) of simulated detection probability for juveniles and adults compared to known and equal detection probability for both age classes (ρ = 0.2; horizontal dashed lines) under 18 different sampling scenarios assuming a low initial abundance with 25% missing data.

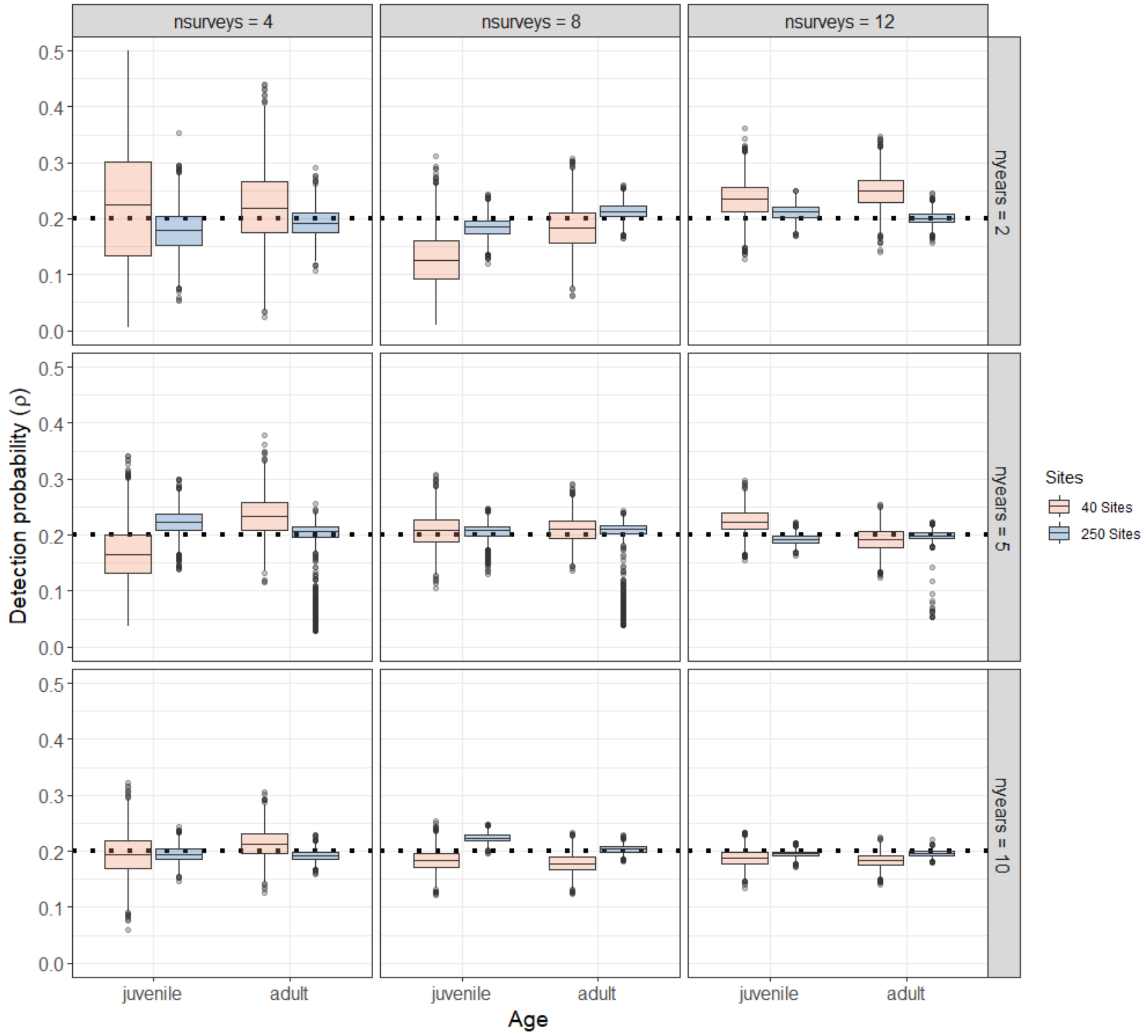

Figure S7. Boxplots of Monte Carlo Markov chain (MCMC) draws (n = 3,000) of simulated detection probability for juveniles and adults compared to known and equal detection probability for both age classes (ρ = 0.2; horizontal dashed lines) under 18 different sampling scenarios assuming a low initial abundance with 50% missing data.

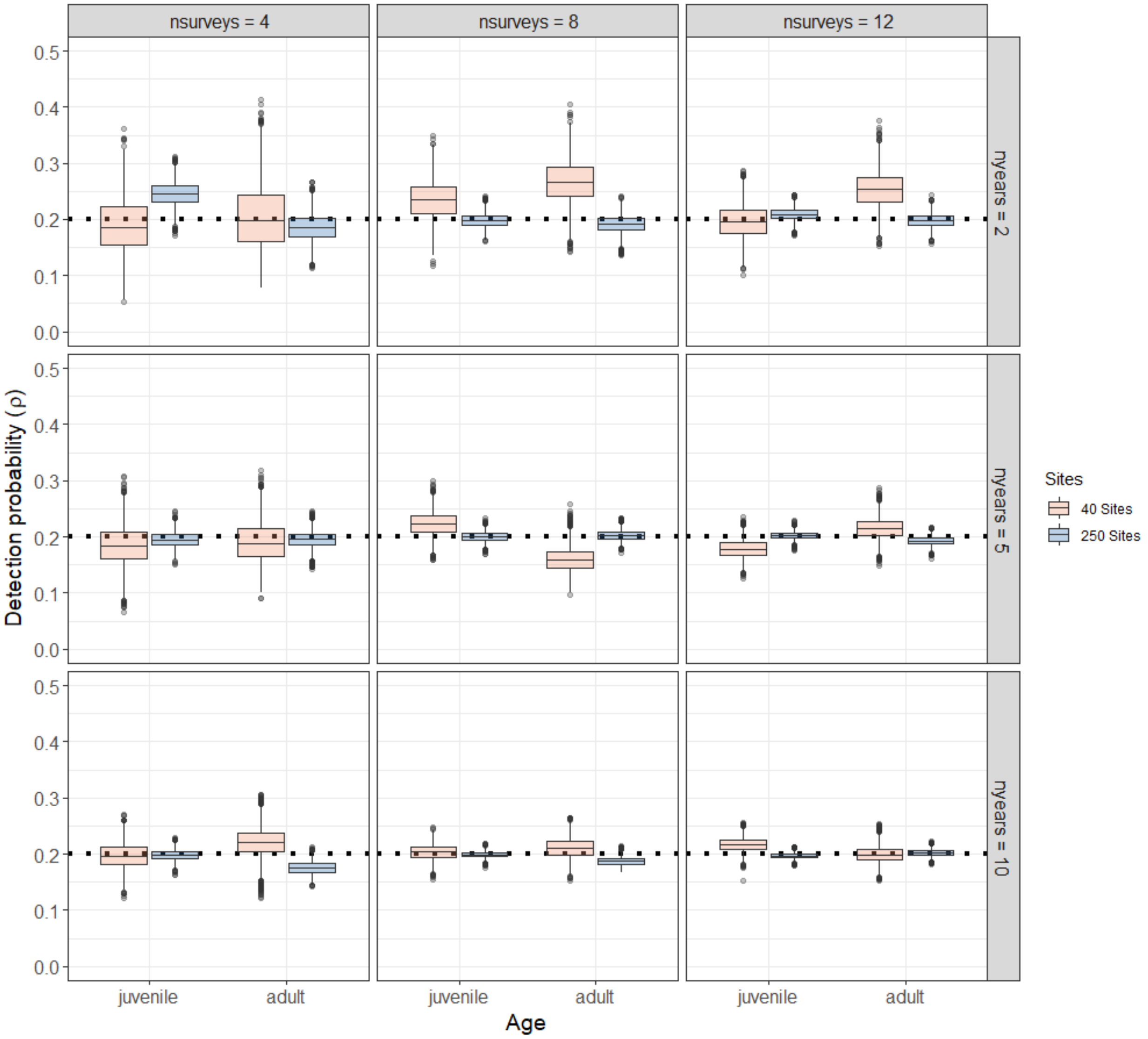

Figure S8. Boxplots of Monte Carlo Markov chain (MCMC) draws (n = 3,000) of simulated detection probability for juveniles and adults compared to known and equal detection probability for both age classes (ρ = 0.2; horizontal dashed lines) under 18 different sampling scenarios assuming a high initial abundance with no missing data.

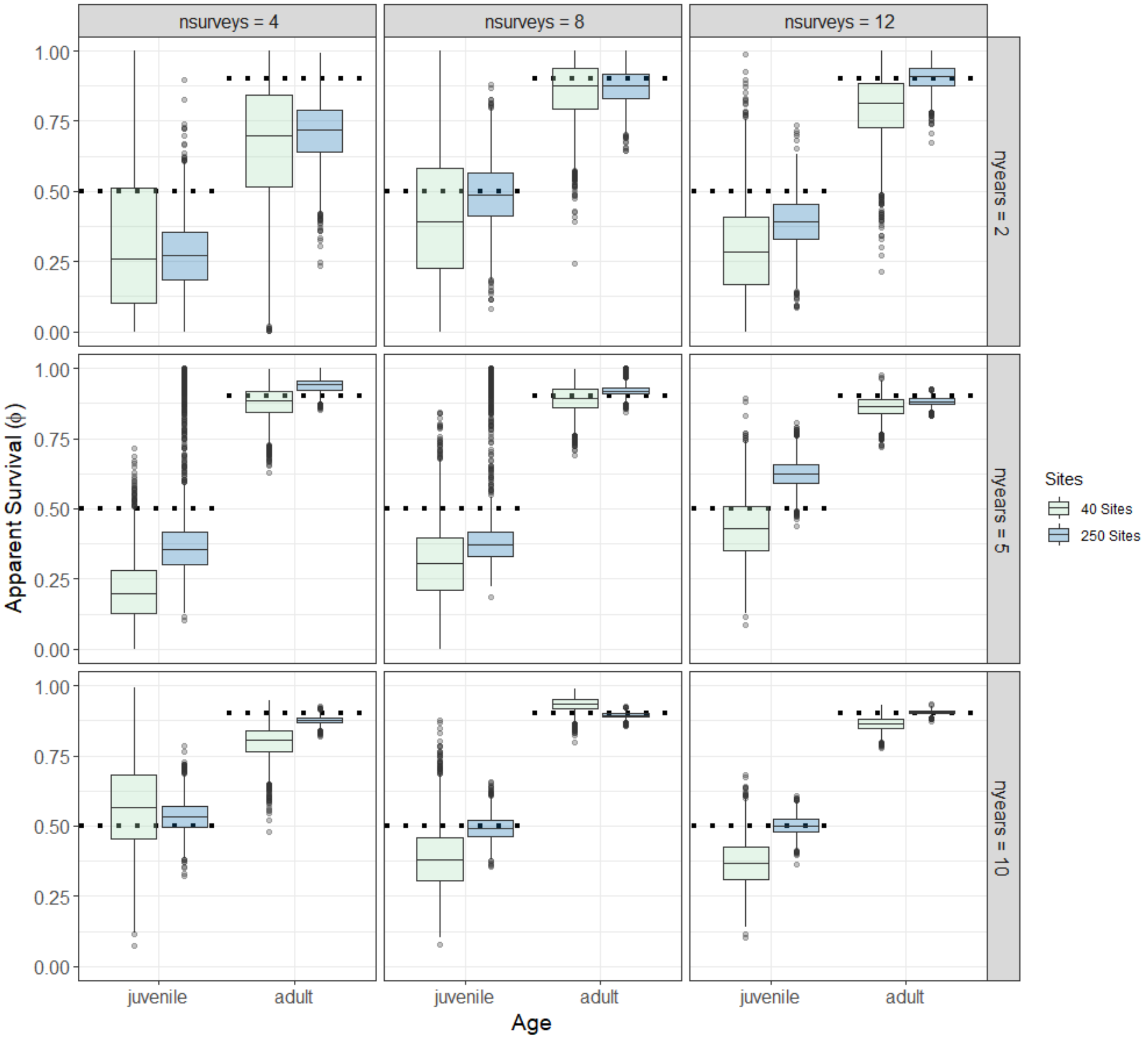

Figure S9. Boxplots of Monte Carlo Markov chain (MCMC) draws (n = 3,000) of simulated apparent survival for juveniles and adults compared to known survival (horizontal dashed lines) under 18 different sampling scenarios assuming a low initial abundance with 25% missing data and equal detection probability for both age classes (ρ = 0.2).

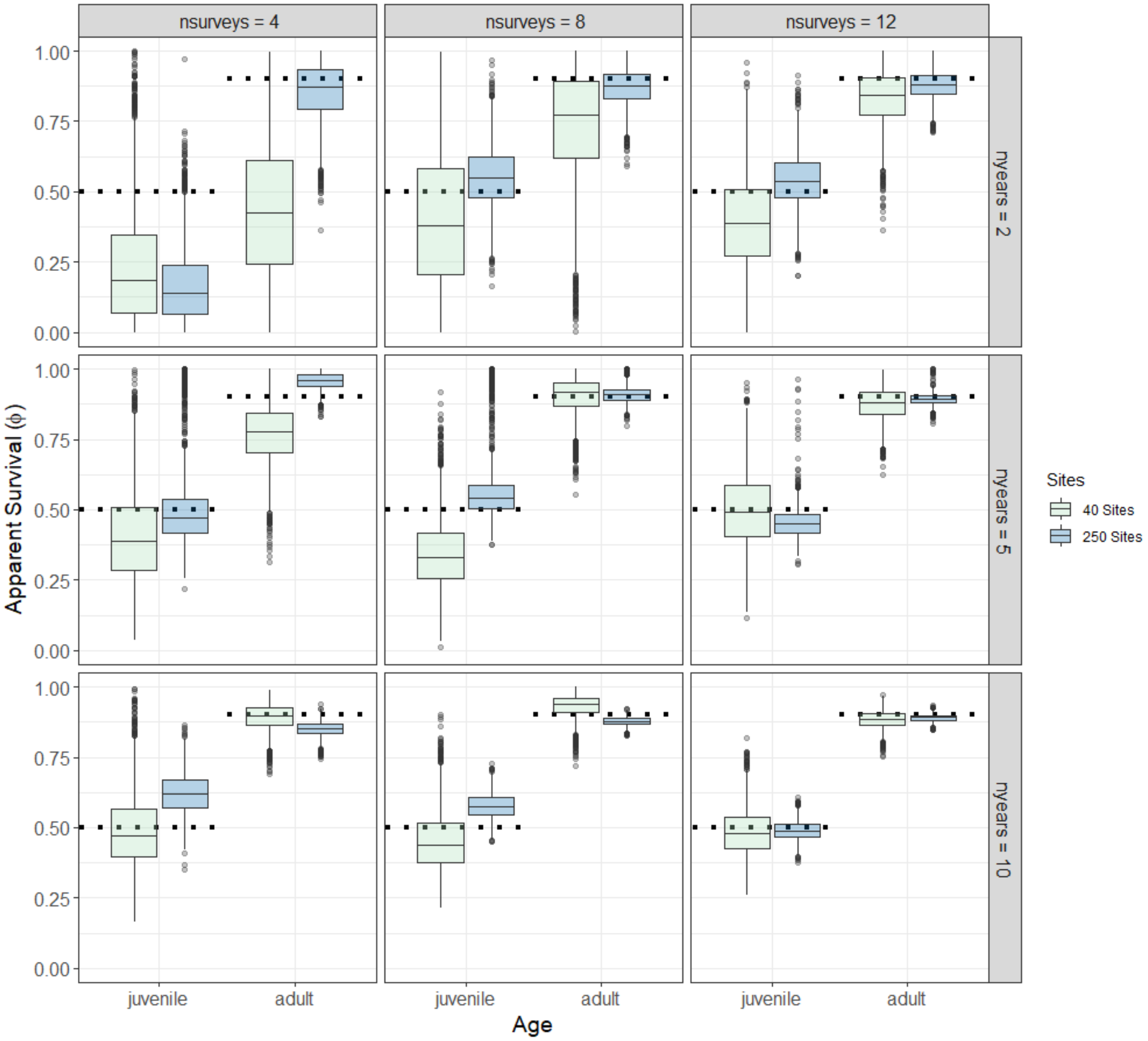

Figure S10. Boxplots of Monte Carlo Markov chain (MCMC) draws (n = 3,000) of simulated apparent survival for juveniles and adults compared to known survival (horizontal dashed lines) under 18 different sampling scenarios assuming a low initial abundance with 50% missing data and equal detection probability for both age classes (ρ = 0.2).

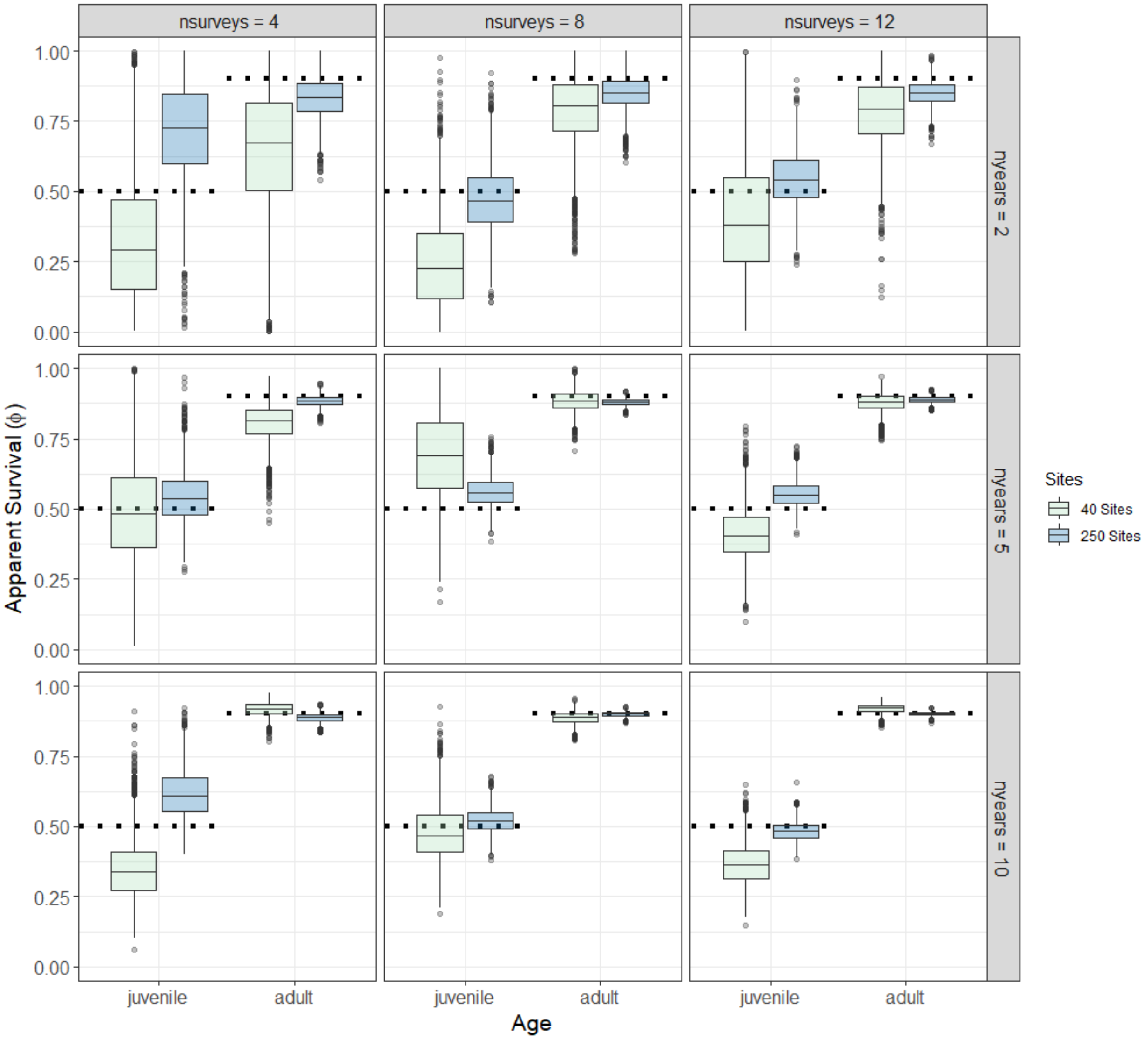

Figure S11. Boxplots of Monte Carlo Markov chain (MCMC) draws (n = 3,000) of simulated apparent survival for juveniles and adults compared to known survival (horizontal dashed lines) under 18 different sampling scenarios assuming a high initial abundance with no missing data and equal detection probability for both age classes (ρ = 0.2).

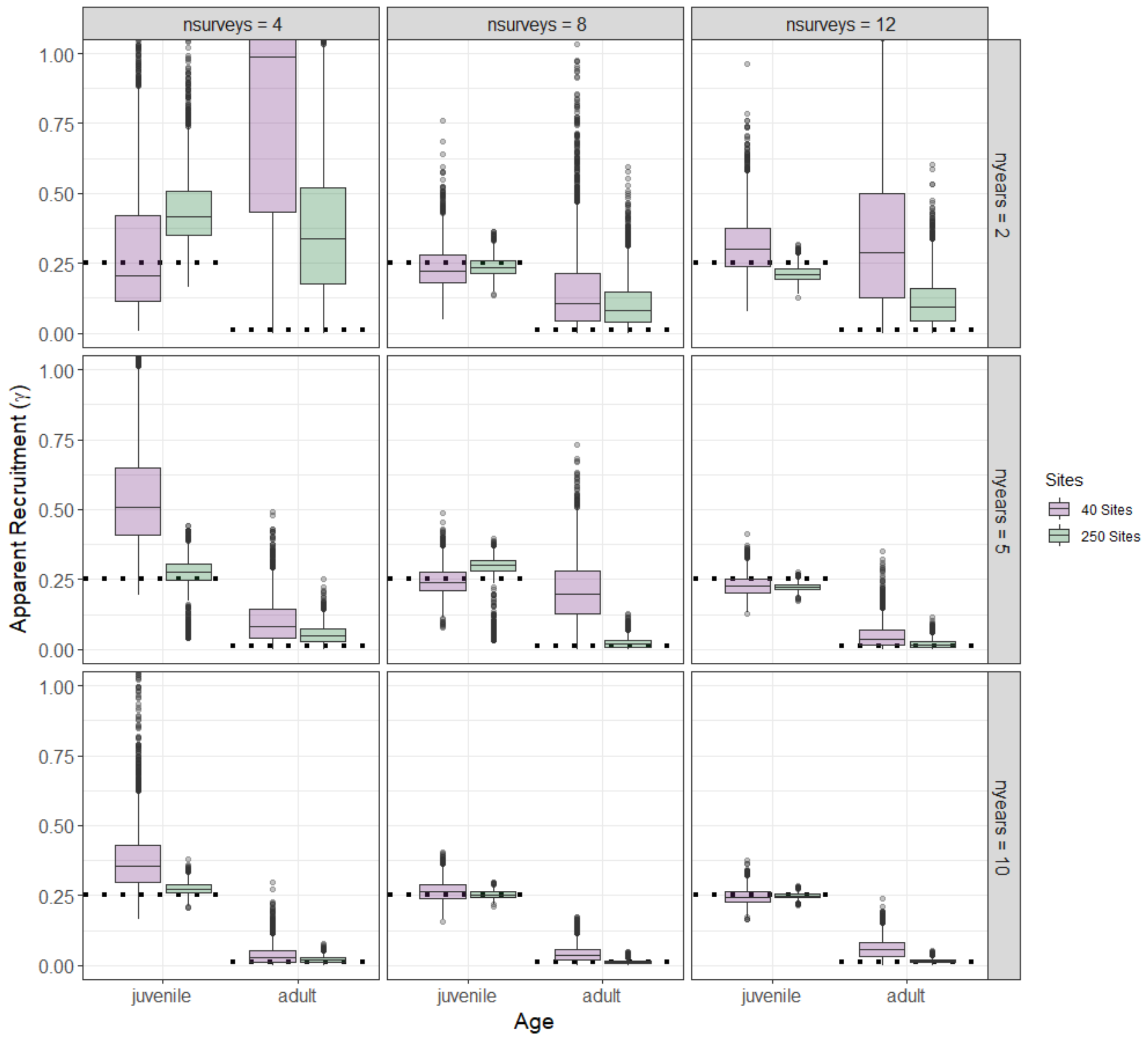

Figure S12. Boxplots of Monte Carlo Markov chain (MCMC) draws (n = 3,000) of simulated apparent recruitment/immigration for juveniles and adults compared to known recruitment (horizontal dashed lines) under 18 different sampling scenarios assuming a low initial abundance with 25% missing data and equal detection probability for both age classes (ρ = 0.2). Note that the y-axis is limited between 0 and 1 to improve comparison across scenarios but the outliers of some boxplots extend considerably higher for some scenarios (e.g., the highest value for apparent recruitment was 24 under a scenario with 40 sites, 2 years, and 4 surveys).

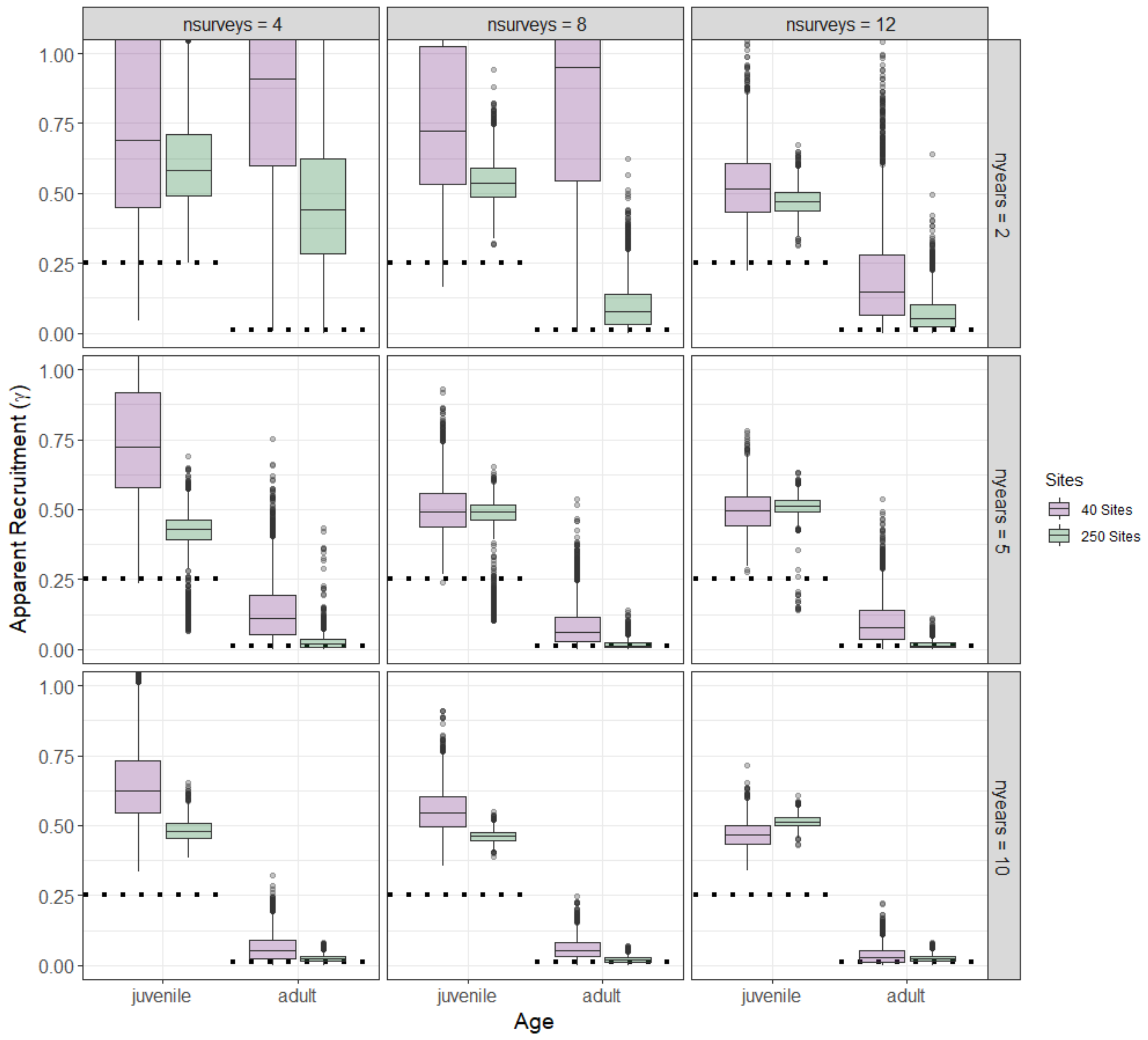

Figure S13. Boxplots of Monte Carlo Markov chain (MCMC) draws (n = 3,000) of simulated apparent recruitment/immigration for juveniles and adults compared to known recruitment (horizontal dashed lines) under 18 different sampling scenarios assuming a low initial abundance with 50% missing data and equal detection probability for both age classes (ρ = 0.2). Note that the y-axis is limited between 0 and 1 to improve comparison across scenarios but the outliers of some boxplots extend considerably higher for some scenarios (e.g., the highest value for apparent recruitment was 26 under a scenario with 40 sites, 2 years, and 4 surveys).

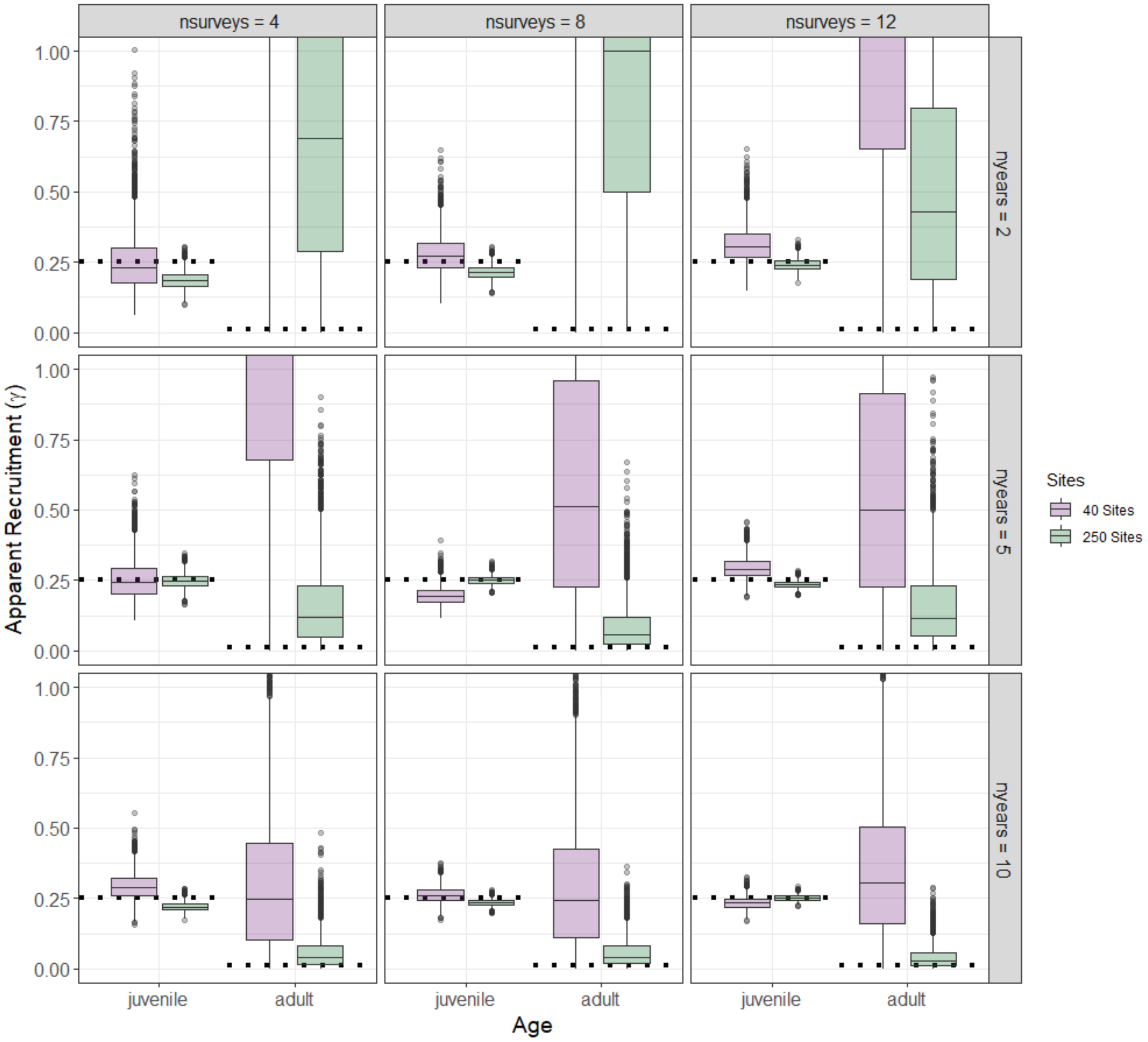

Figure S14. Boxplots of Monte Carlo Markov chain (MCMC) draws (n = 3,000) of simulated apparent recruitment/immigration for juveniles and adults compared to known recruitment (horizontal dashed lines) under 18 different sampling scenarios assuming a high initial abundance with no missing data and equal detection probability for both age classes (ρ = 0.2). Note that the y-axis is limited between 0 and 1 to improve comparison across scenarios but the outliers of some boxplots extend considerably higher for some scenarios (e.g., the highest value for apparent recruitment was 49 under a scenario with 40 sites, 2 years, and 4 surveys).

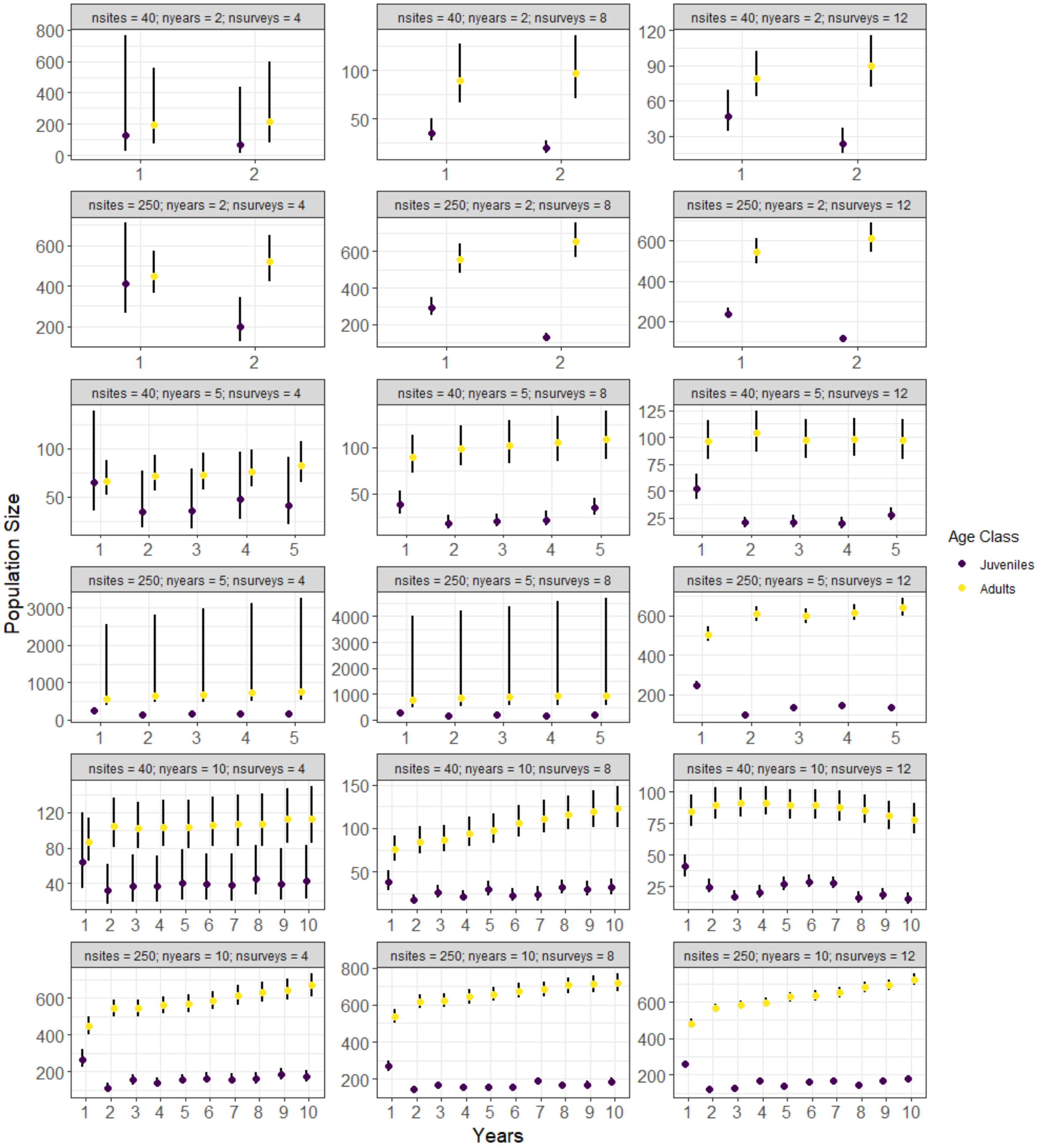

Figure S15. Simulated estimates of population size and 95% credible intervals for each age class under 18 different sampling scenarios assuming a low initial abundance with 25% missing data and equal detection probability for both age classes (ρ = 0.2). The circles indicated parameter estimates, and the lines indicate the 95% credible intervals.

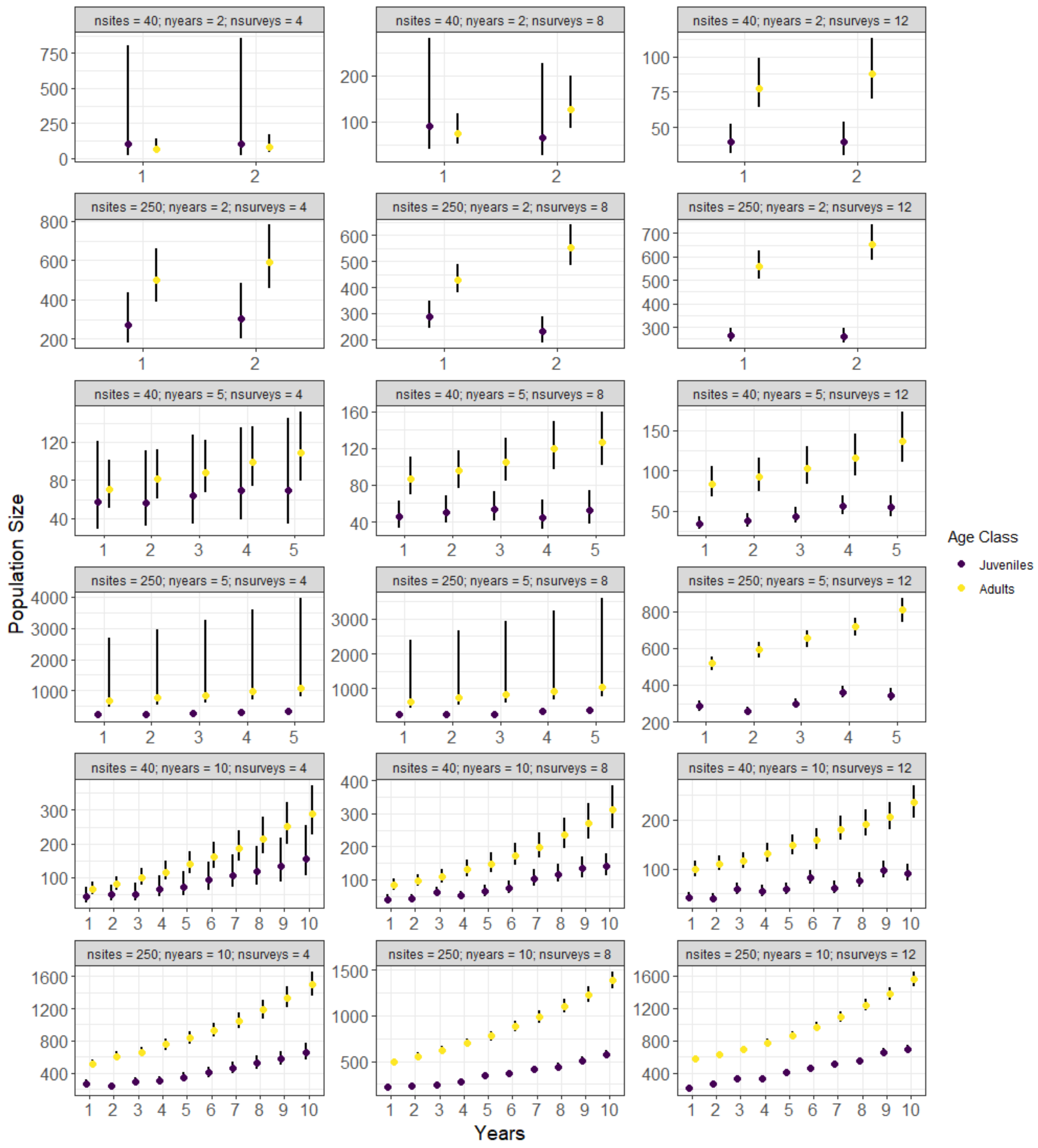

Figure S16. Simulated estimates of population size and 95% credible intervals for each age class under 18 different sampling scenarios assuming a low initial abundance with 50% missing data and equal detection probability for both age classes (ρ = 0.2). The circles indicated parameter estimates, and the lines indicate the 95% credible intervals.

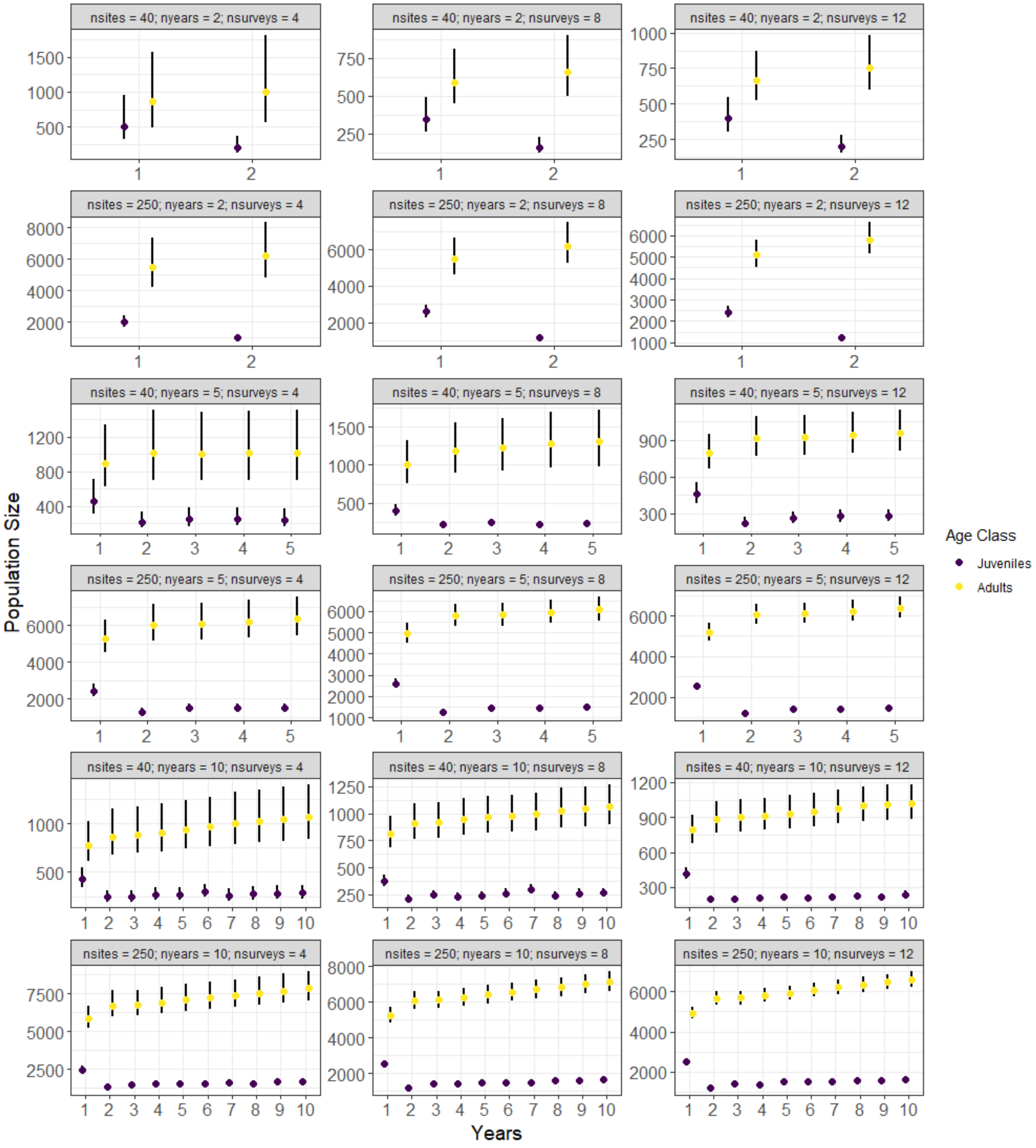

Figure S17. Simulated estimates of population size and 95% credible intervals for each age class under 18 different sampling scenarios assuming a high initial abundance with no missing data and equal detection probability for both age classes (ρ = 0.2). The circles indicated parameter estimates, and the lines indicate the 95% credible intervals.

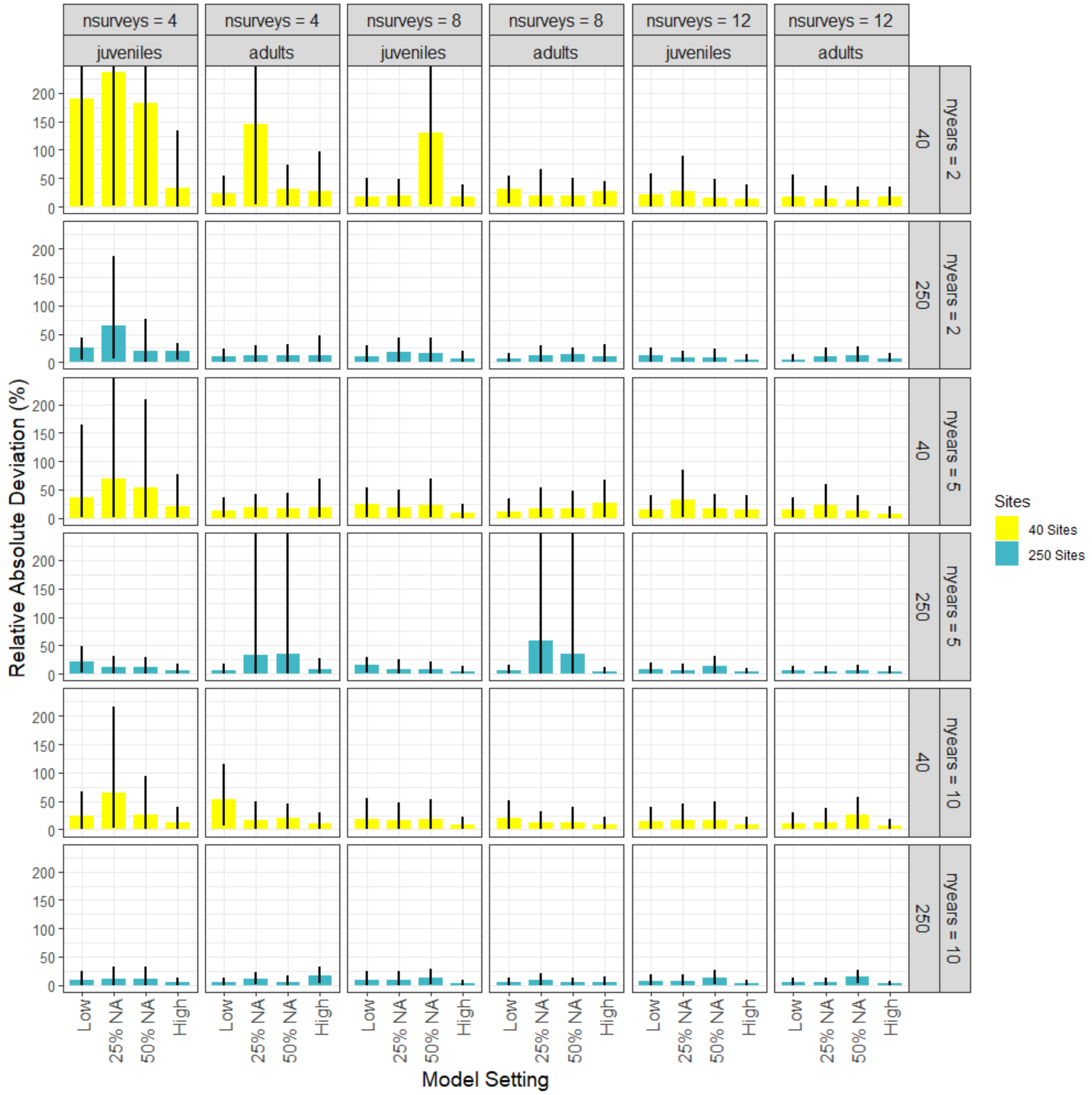

Figure S18. Mean and lower (2.75%) and upper (97.5%) quantiles (error bars) of relative absolute deviation (RAD), expressed as the percent difference between known initial abundances and those estimated using a multistate Dail-Madsen (DM) model of 2 states (juvenile and adult) under 18 different combinations of sites, years, and surveys and 4 different model settings, including low and high initial abundance and 25 and 50% missing data (NA). Note, that the missing data settings were only conducted using a low initial abundance and the y-axis is limited between 0 and 225 to improve visual comparison across scenarios but the error bars extend considerably higher for some scenarios (e.g., the highest RAD value for initial abundance of juveniles was 2,399.78% under a scenario with 40 sites, 2 years, and 4 surveys [see Table S5]).

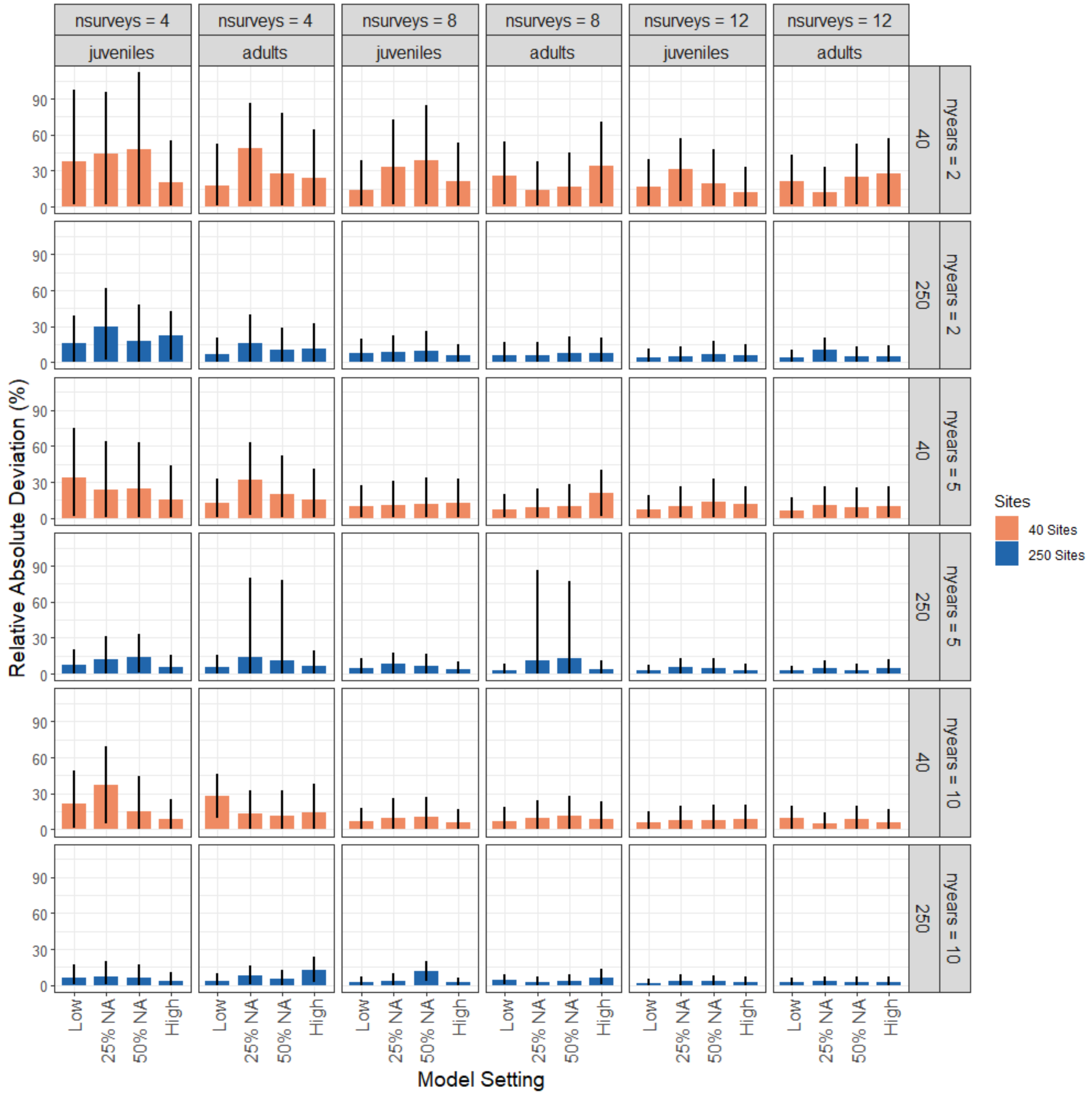

Figure S19. Mean and lower (2.75%) and upper (97.5%) quantiles (error bars) of relative absolute deviation (RAD), expressed as the percent difference between known detection probabilities and those estimated using a multistate Dail-Madsen (DM) model of 2 states (juvenile and adult) under 18 different combinations of sites, years, and surveys and 4 different model settings, including low and high initial abundance and 25 and 50% missing data (NA). Note, that the missing data settings were only conducted using a low initial abundance.

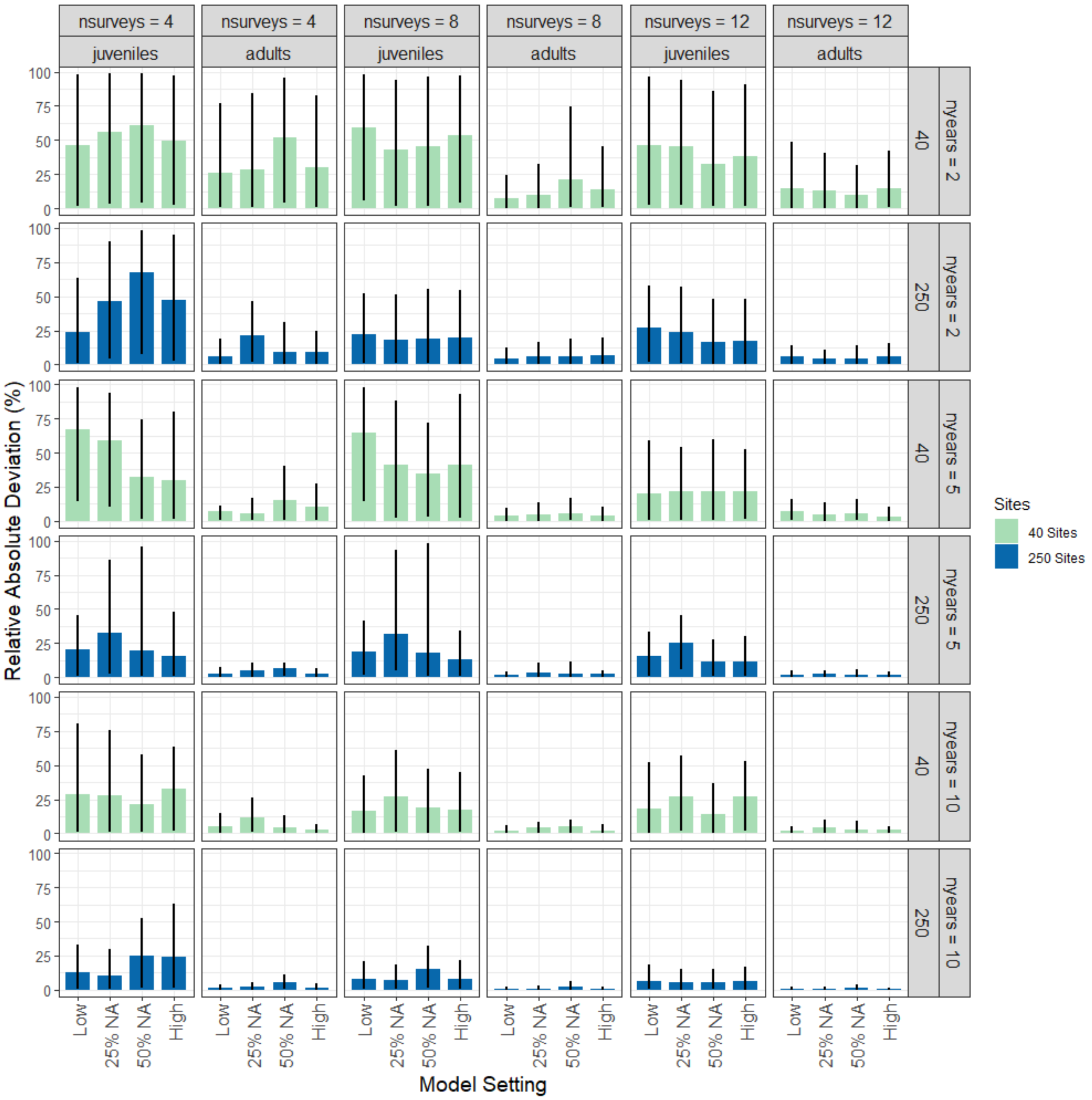

Figure S20. Mean and lower (2.75%) and upper (97.5%) quantiles (error bars) of relative absolute deviation (RAD), expressed as the percent difference between known apparent survival and that estimated using a multistate Dail-Madsen (DM) model of 2 states (juvenile and adult) under 18 different combinations of sites, years, and surveys and 4 different model settings, including low and high initial abundance and 25 and 50% missing data (NA).

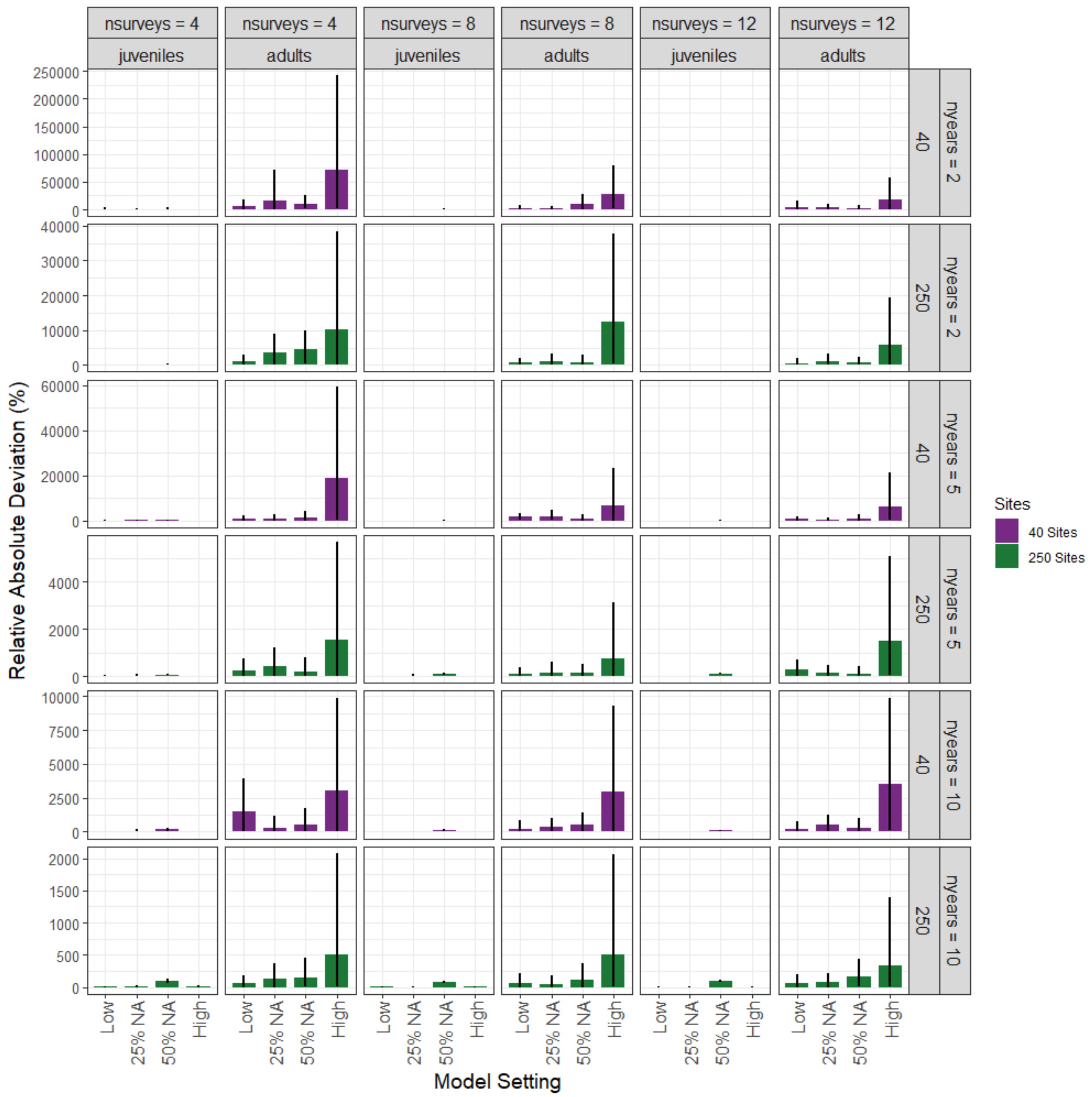

Figure S21. Mean and lower (2.75%) and upper (97.5%) quantiles (error bars) of relative absolute deviation (RAD), expressed as the percent difference between known apparent recruitment and that estimated using a multistate Dail-Madsen (DM) model of 2 states (juvenile and adult) under 18 different combinations of sites, years, and surveys and 4 different model settings, including low and high initial abundance and 25 and 50% missing data (NA). Note, that the missing data settings were only conducted using a low initial abundance and the y-axis varies between year and sample size scenarios to improve visual comparison across scenarios but the error bars extend considerably higher for some scenarios (e.g., the highest RAD value for apparent recruitment of adults was 242,157.61% under a scenario with 40 sites, 2 years, and 4 surveys [see Table S5]).

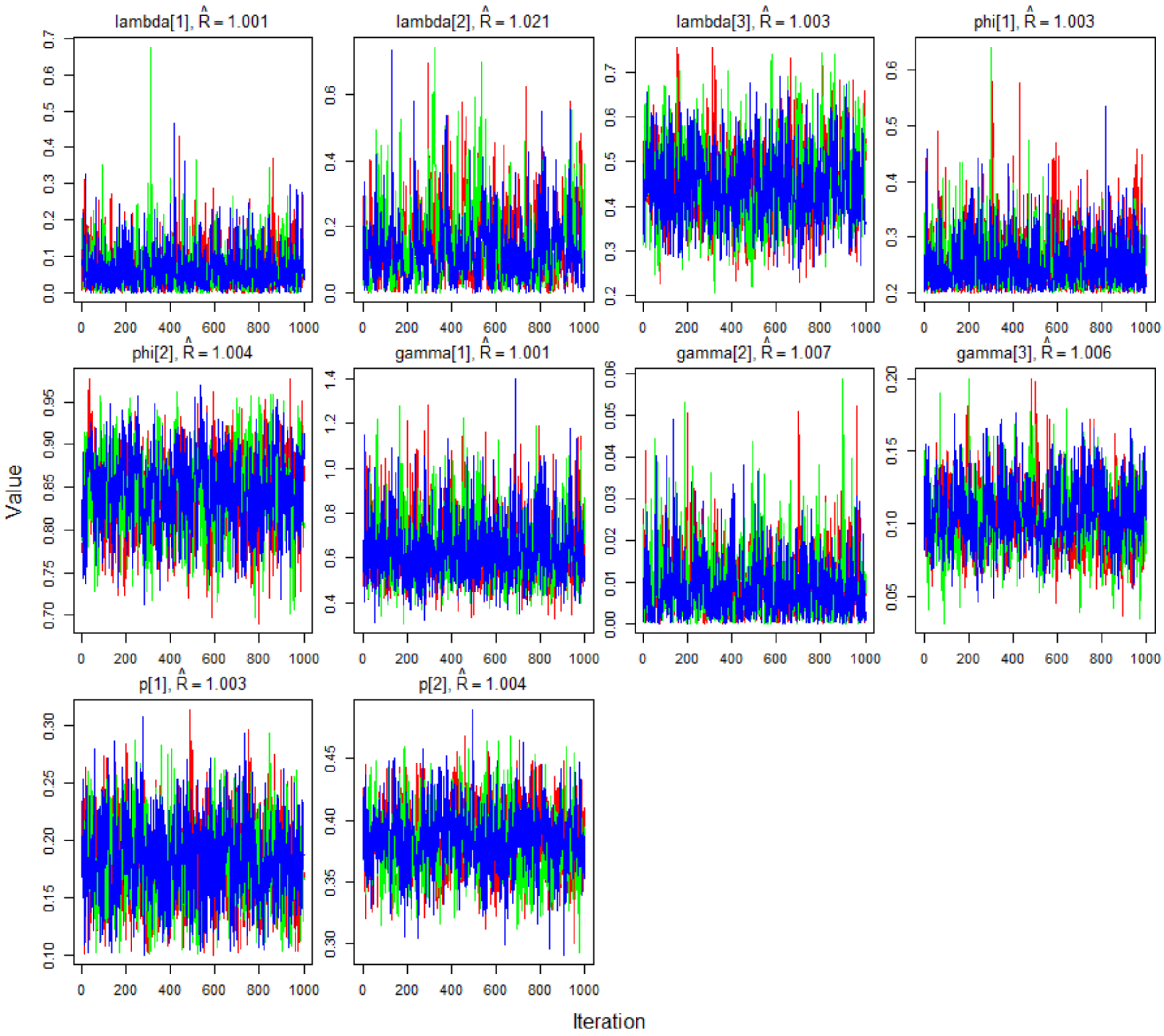

Figure S22. Traceplots of initial abundance (calves = lambda[1], yearlings = lambda[2], and adults = lambda[3]), apparent survival (calves = phi[1], and adults = phi[2]), apparent recruitment (calves = gamma[1], yearlings = gamma [2], and adults = gamma[3]), and detection probability (calves = rho[1], and adults = rho[2]) for a multistate DM model to evaluate moose population dynamics in New Hampshire and Vermont from 2014–2019.
